# Adipocyte death promotes hepatic infiltration of S100A8^+^ macrophages and steatotic liver disease progression in mice

**DOI:** 10.1172/JCI190635

**Published:** 2025-11-03

**Authors:** Yukun Guan, Yeonsoo Kim, Yang Wang, Ye Eun Cho, Xiaogang Xiang, Seung-Jin Kim, Tiantian Yao, Dechun Feng, Seonghwan Hwang, Bin Gao

**Affiliations:** 1 Laboratory of Liver Diseases, National Institute on Alcohol Abuse and Alcoholism (NIAAA), NIH, Bethesda, Maryland, USA.; 2 College of Pharmacy and Research Institute for Drug Development, Pusan National University, Busan, South Korea.

**Keywords:** Gastroenterology, Hepatology, Adipose tissue, Macrophages

## Abstract

Both adipocytes and hepatocytes have the capacity to store fat, but the factor(s) that determine fat distribution between these cell types remain unknown. In mice fed a high-fat diet, fat initially accumulates predominantly in adipocytes, while hepatic fat accumulation mainly emerges after the onset of epididymal adipocyte death that results in elevated free fatty acids to promote lipid accumulation in hepatocytes. However, it remains unclear whether other signals after adipocyte death are required to direct and/or promote hepatocytes to store fat and subsequently trigger metabolic dysfunction–associated steatotic liver disease (MASLD, formerly known as nonalcoholic fatty liver disease). Using genetically modified mouse models combined with bulk and single-cell RNA-Seq analysis, we demonstrated that visceral adipocyte death induced an accumulation of S100A8^+^ macrophages in the liver, which was partially induced by fatty acids and apoptotic adipocyte–derived extracellular vesicles. Macrophage-specific deletion of the *S100a8* gene reduced hepatic fat accumulation and MASLD severity in mice. Mechanistically, S100A8^+^ macrophages suppressed cellular communication network factor 3 (CCN3), a negative regulator of CD36, thereby enhancing CD36 expression in hepatocytes. In conclusion, adipocyte death promotes hepatic infiltration of S100A8^+^ macrophages, which drive hepatocyte lipid storage and subsequently promote MASLD progression through CD36 upregulation, partially mediated by CCN3 suppression.

## Abstract 2

Macrophages promote fat redistribution from adipocytes to hepatocytes and development of steatotic liver disease

## Graphical abstract

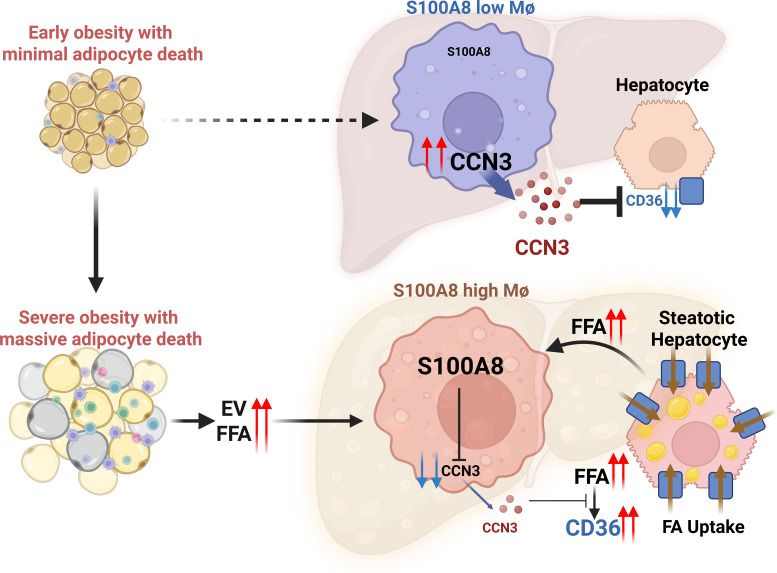

## Introduction

The recent prevalence of metabolic disease has rapidly increased the incidence of metabolic dysfunction–associated steatotic liver disease (MASLD), which includes a spectrum of liver disorders that range from steatosis to metabolic dysfunction–associated steatohepatitis (MASH), cirrhosis, and hepatocellular carcinoma (1–3). Steatosis, the excessive accumulation of lipids in the liver, affects approximately 30% of the adult population worldwide (4, 5) and is considered the primary condition of MASH development (5), which is characterized by liver injury, inflammation, and fibrosis in addition to fat accumulation in the liver (1–3).

The molecular mechanisms underlying obesity-associated steatosis have been extensively investigated, including elevated de novo lipogenesis, suppressed β-oxidation, enhanced fatty acid uptake, and interplay with other organs, such as gut and adipose tissue (6, 7). Notably, in mice fed a high-fat diet (HFD), lipid accumulation occurs primarily in adipose tissue during the early stages, and marked hepatic steatosis (lipid accumulation in hepatocytes) emerges later, coinciding with elevated free fatty acid (FFA) levels that promote lipid storage in hepatocytes (6, 7). However, it remains unknown whether additional signals are required to drive lipid storage in hepatocytes during obesity.

Obesity is associated with increased adipocyte death in inflamed adipose tissue (8, 9). Adipose tissue remodeling during obesity induces cellular stress, leading to adipocyte death and inflammation (10, 11). Several studies have implicated adipocyte death as a contributing factor to hepatic steatosis. For example, inhibition of adipocyte apoptosis via deletion of proapoptotic genes, such *Fas* and *Bid*, ameliorated HFD-induced steatosis in mice (12, 13). In the present study, we observed that adipocyte death and the shrinkage of epididymal adipose tissue coincided with the onset of hepatic fat accumulation and liver enlargement after 3 months of HFD feeding, suggesting a redistribution of lipids from adipose tissue to the liver. However, the molecular mechanism linking adipocyte death to lipid redistribution into hepatocytes remains poorly defined. To address how adipocyte death generates signals directing hepatocytes to store fat during obesity development, we employed a mouse model with adipocyte-specific overexpression of B cell leukemia/lymphoma 2 (BCL2), an antiapoptotic protein essential for attenuating apoptosis (14), whose expression is decreased in the adipose tissues of obese individuals (15). Adipocyte-specific overexpression of the *Bcl2* gene mitigated HFD-induced steatosis and MASLD progression in mice, accompanied by notable alterations in the hepatic infiltration of macrophages, particularly a population of S100A8^+^ macrophages identified via single-cell RNA-Seq (scRNA-Seq) analysis.

S100A8 (also known as myeloid-related protein-8, MRP8) is a calcium-binding protein primarily expressed by immune cells, such as neutrophils and macrophages (16, 17). S100A8 forms a heterodimer with S100A9 (also known as MRP14) and functions as a damage-associated molecular pattern (DAMP) that activates innate immunity (18). S100A8/S100A9 levels are increased in the serum of patients with MASLD, suggesting their contribution to MASLD pathogenesis (19). Experimental models have shown that neutrophil-derived S100A8 contributes to disease progression in MASLD and other conditions (20–22), but conclusive evidence for the in vivo functions of S100A8 has been limited due to lack of *S100a8*-KO mice (global *S100a8*-KO mice are embryonic lethal) (23). In macrophages, S100A8/A9 promotes inflammatory responses via pathways involving TLR4, NF-κB, RAGE, and p38 MAPK (24–26). Single-cell transcriptomic studies have linked S100A8^+^ macrophages to liver fibrosis (27), and S100A8-mediated pyroptotic macrophage death has been shown to activate hepatic stellate cells and promote liver fibrosis (28). These studies have suggested the possibility that S100A8^+^ macrophages modulate the inflammatory and fibrotic processes related to the pathogenesis of liver disease. However, the role of S100A8^+^ macrophages in hepatic lipid accumulation, particularly in the context of adipocyte death–induced lipid redistribution, remains unknown. To address this, we generated macrophage-specific *S100a8*-KO mice and observed a significant reduction in hepatic steatosis, accompanied by decreased expression of the fatty acid transporter CD36. This approach circumvents the developmental limitations of global *S100a8* deletion and provides direct evidence that S100A8^+^ macrophages facilitate lipid redistribution during MASLD progression. To our knowledge, this is the first study to demonstrate a functional role for S100A8^+^ macrophages in regulating interorgan fat redistribution between the liver and adipose tissue.

Cellular communication network factor 3 (CCN3), an extracellular matrix–associated protein, plays diverse roles in tissue remodeling and disease pathogenesis (29, 30). Although adipocyte-derived CCN3 has been implicated in promoting adiposity (31), CCN3 also exhibits antiinflammatory and antifibrotic functions in the vasculature, kidney, and liver (32–36). Myeloid-specific deletion of *Ccn3* has been shown to exacerbate hepatic lipid accumulation, injury, and inflammation, in part via upregulation of CD36 (37). However, the specific myeloid subpopulation involved and the direct impact of CCN3 on hepatocyte CD36 expression were not elucidated.

In this study, we demonstrated that S100A8^+^ macrophages promote hepatic fat accumulation by suppressing CCN3. Moreover, recombinant CCN3 directly downregulated CD36, with an inhibition of its upstream regulator, PPARγ. These findings support a model in which adipocyte death increases hepatic S100A8^+^ macrophage infiltration, which in turn promotes steatosis by repressing CCN3 and upregulating CD36 expression in hepatocytes.

## Results

### Temporal correlation between epididymal adipocyte death and hepatic steatosis after HFD feeding.

**Figure 1:**
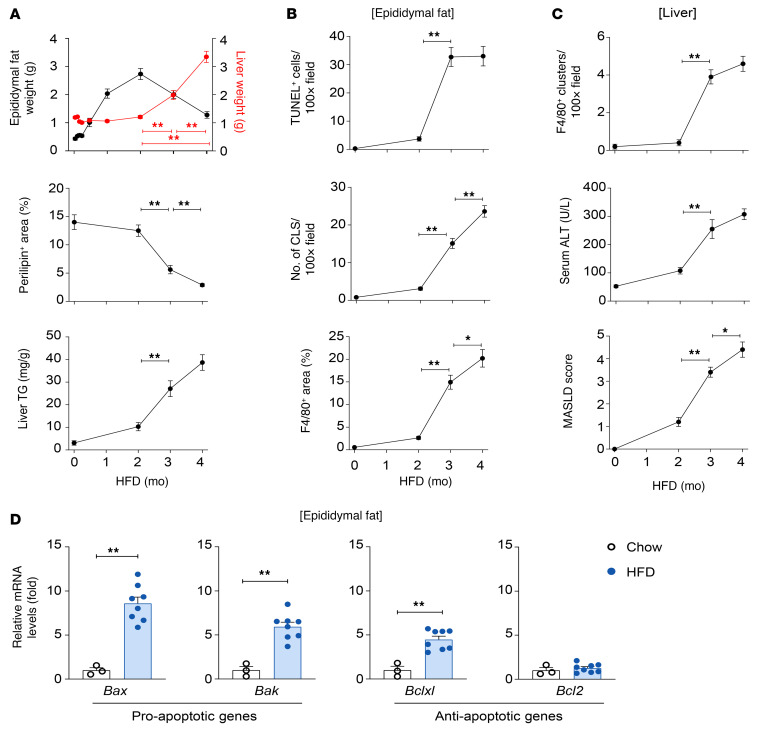
Adipocyte death is correlated with liver fat accumulation and MASLD severity during high-fat diet feeding in mice. (**A**–**C**) C57BL/6J mice (male) were fed a high-fat diet (HFD) for the specified durations. (**A**) Temporal changes in epididymal fat weight (black) and liver weight (red) (top). Area positive for immunofluorescence of perilipin in epididymal fat (middle). Hepatic triglyceride content (bottom). (**B**) Number of TUNEL-positive cells in epididymal fat (top). Number of crown-like structures in epididymal fat (middle). F4/80-positive area in epididymal fat (bottom). (**C**) Number of F4/80-positive clusters in the liver (top). Serum alanine aminotransferase levels (middle). MASLD score (bottom). Representative images for quantification and analysis are included in Supplemental Figures 1 and 2. (**D**) C57BL/6J mice (male) were fed an HFD or chow diet for 3 months. Epididymal fat mRNA levels were assessed by reverse transcription quantitative PCR (RT-qPCR) analysis. Values represent the mean ± SEM. Statistical evaluation was performed using Student’s *t* test or 1-way ANOVA with Tukey’s post hoc test for multiple comparisons (*P < 0.05; **P < 0.01).

To examine the temporal relationship between pathological changes in the adipose tissue and liver during MASLD progression, we analyzed organ weights and pathological markers in HFD-fed mice. Mice showed a continuous increase in body weight and subcutaneous fat weight over 4 months of HFD feeding (Supplemental Figure 1A; supplemental material available online with this article; https://doi.org/10.1172/JCI190635DS1), whereas epididymal fat weight peaked at 2 months and decreased thereafter (Figure 1A). Interestingly, the reduction in epididymal fat weight coincided with an increase in liver weight, which remained unchanged until 2 months of HFD feeding and increased thereafter (Figure 1A). The reduction in epididymal fat weight was associated with a corresponding decrease in the expression of perilipin (a lipid droplet membrane protein), and the increase in liver weight corresponded to elevated levels of liver triglycerides (Figure 1A and Supplemental Figure 1B). Perilipin is expressed on the surfaces of adipocyte lipid droplets (38), and its loss is a recognized hallmark of dead adipocytes (39).

To verify whether the reduction in epididymal fat is linked to adipocyte death, we conducted TUNEL, assessed crown-like structures, and performed F4/80 macrophage staining. Our data revealed a significant increase in TUNEL-positive cells in epididymal fat after 3 months of HFD feeding compared with 2 months. Additionally, both crown-like structure formation and the F4/80-positive area in epididymal fat were notably elevated at 3 months (Figure 1B and Supplemental Figure 1B), indicating an increase of dead cells and inflammation in epididymal fat.

Hepatic F4/80-positive clusters, serum alanine aminotransferase (ALT), and MASLD scores were significantly higher at 3 months compared with 2 months (Figure 1C and Supplemental Figure 2A), indicating that steatosis became more pronounced at 3 months. Furthermore, hepatic mRNA levels of inflammatory and fibrogenic genes increased at 3 months and remained consistently elevated thereafter (Supplemental Figure 2B).

It is well established that female mice are more resistant to the development of obesity and steatosis in part due to estrogen signaling (40). However, the temporal relationship between adipocyte death/inflammation and liver steatosis has not been thoroughly explored in the context of sex differences. To address this, we subjected female mice to 4 months of HFD feeding. In contrast with male mice, female mice exhibited marked resistance to adipose crown-like structure formation and macrophage infiltration, and their periovarian adipose tissue continued to expand throughout the 4-month period (Supplemental Figure 3). The number of crown-like structures in 4-month HFD-fed female mice was around 8 per 100× field (Supplemental Figure 3C), which was much lower than that (~25 per 100× field) in 4-month HFD-fed male mice (Figure 1B). This suggests that female adipocytes are more resistant to cell death compared with those in males. Correspondingly, the liver weight of HFD-fed female mice increased gradually over time but remained below 1.3 g, substantially lower than the approximately 3.5 g observed in male counterparts, as described in Figure 1. These findings indicate that the resistance of female mice to HFD-induced hepatic steatosis may be linked to their reduced adipocyte death and associated inflammatory responses.

To explore mechanisms underlying adipocyte death after HFD feeding, we assessed the expression of proapoptotic and antiapoptotic genes. As shown in Figure 1D, 3 months of HFD feeding in mice significantly upregulated *Bax* and *Bak*, key proapoptotic factors, in epididymal fat. Interestingly, *Bcl-xL*, an antiapoptotic gene, was also upregulated, potentially as a compensatory mechanism to counteract apoptosis. In contrast, *Bcl2*, another major antiapoptotic factor, showed no significant upregulation in epididymal adipose tissues after HFD feeding. Taken together, these findings suggest that epididymal fat reduction and adipocyte death align temporally with the onset of hepatic steatosis during prolonged HFD feeding in mice.

### Blockade of adipocyte death prevents fat redistribution from adipocytes to hepatocytes and ameliorates MASLD in HFD-fed mice.

As mentioned above, 3 months of HFD feeding triggered a compensatory upregulation of *Bcl-xL*, whereas the expression of *Bcl2*, another antiapoptotic factor, was not induced by HFD feeding. This led us to hypothesize that targeted overexpression of the *Bcl2* gene in adipocytes could prevent adipocyte death, reduce lipid redistribution to hepatocytes, and ameliorate MASLD progression. To test this hypothesis, we developed an adipocyte-specific transgenic mouse model (termed *Bcl2*^AdTG^ mice) engineered to overexpress *Bcl2* exclusively in adipocytes.

**Figure 2:**
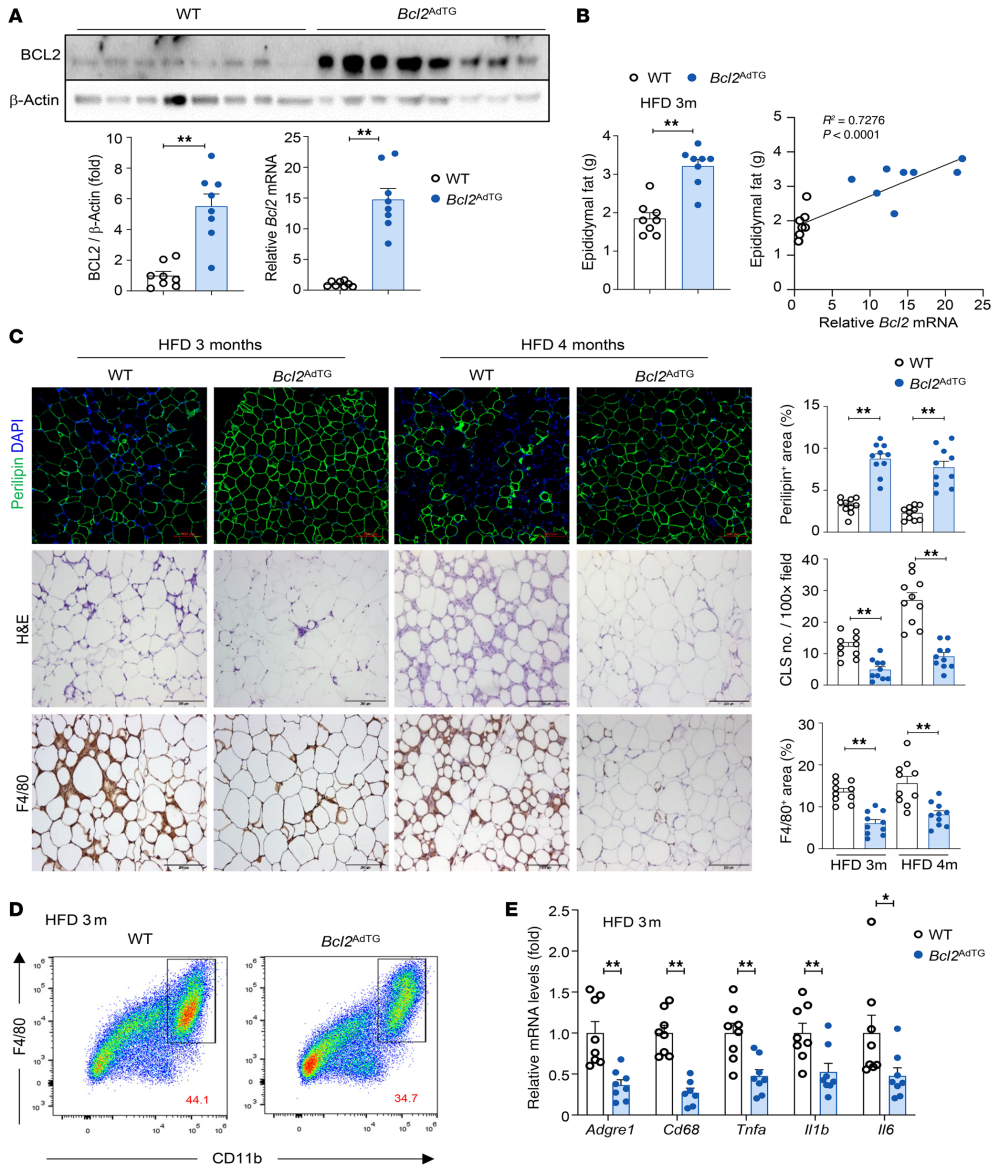
Overexpression of the *Bcl2* gene in adipocytes reduces high-fat diet–induced adipocyte death in mice. Male *Bcl2*^AdTG^ mice and WT littermates were fed a high-fat diet (HFD) for 3 or 4 months, and epididymal fat tissues were collected for analyses. (**A**) Immunoblot analysis of BCL2 (top) and the quantification of BCL2 protein normalized to β-actin (bottom left). RT-qPCR analysis of *Bcl2* (bottom right). (**B**) Epididymal fat weight (left). The correlation between epididymal fat weight and *Bcl2* mRNA levels (right). Solid line indicates linear regression. (**C**) Perilipin staining (top), H&E staining (middle), and F4/80 staining (bottom) of epididymal fat tissues. Scale bars: 200 μm. (**D**) Flow cytometry analysis of F4/80 and CD11b in the stromal vascular fraction from epididymal fat tissues of mice fed an HFD for 3 months. The values in red represent the percentage of CD11b^+^F4/80^+^ cells. (**E**) RT-qPCR analysis of inflammatory genes in epididymal fat tissues of 3-month HFD-fed mice. Values represent mean ± SEM. Statistical evaluation was performed using Student’s *t* test (*P < 0.05; **P < 0.01).

*Bcl2*^AdTG^ mice displayed markedly elevated levels of both *Bcl2* mRNA and BCL2 protein in epididymal fat compared with WT mice after 3 months of HFD feeding (Figure 2A). Remarkably, epididymal fat weight was significantly higher in *Bcl2*^AdTG^ mice than WT mice after 3 and 4 months of HFD feeding (Figure 2B and Supplemental Figure 4A). Neither body weight nor subcutaneous fat weight was different between WT mice and *Bcl2*^AdTG^ mice on an HFD (Supplemental Figure 4A). A positive correlation was observed between epididymal fat weight and *Bcl2* mRNA levels in *Bcl2*^AdTG^ mice (Figure 2B). The loss of perilipin expression in epididymal fat observed after 3 and 4 months of HFD feeding was markedly attenuated in *Bcl2*^AdTG^ mice (Figure 2C), highlighting the protective role of BCL2 in preventing HFD-induced adipocyte death. This finding was further confirmed by the reduction in TUNEL^+^ cells in the epididymal fat of *Bcl2*^AdTG^ mice after 4 months of HFD feeding compared with WT mice (Supplemental Figure 5A). Moreover, the reduction in adipocyte death due to BCL2 overexpression alleviated HFD-induced inflammation and fibrosis in epididymal fat, as demonstrated by a decrease in crown-like structure formation, F4/80^+^ areas, and Sirius red^+^ areas (Figure 2C and Supplemental Figure 5B). In contrast with epididymal fat, crown-like structure formation and the loss of perilipin expression were not observed in the subcutaneous fat of WT mice after 4 months of HFD feeding, and these changes remained unaffected by BCL2 overexpression (Supplemental Figure 4B). In epididymal fat, the F4/80^+^CD11b^+^ cell population within the stromal vascular fraction was notably reduced in *Bcl2*^AdTG^ mice compared with WT mice (Figure 2D). This macrophage reduction corresponded to a reduced expression of inflammatory genes in epididymal fat of *Bcl2*^AdTG^ mice (Figure 2E).

**Figure 3:**
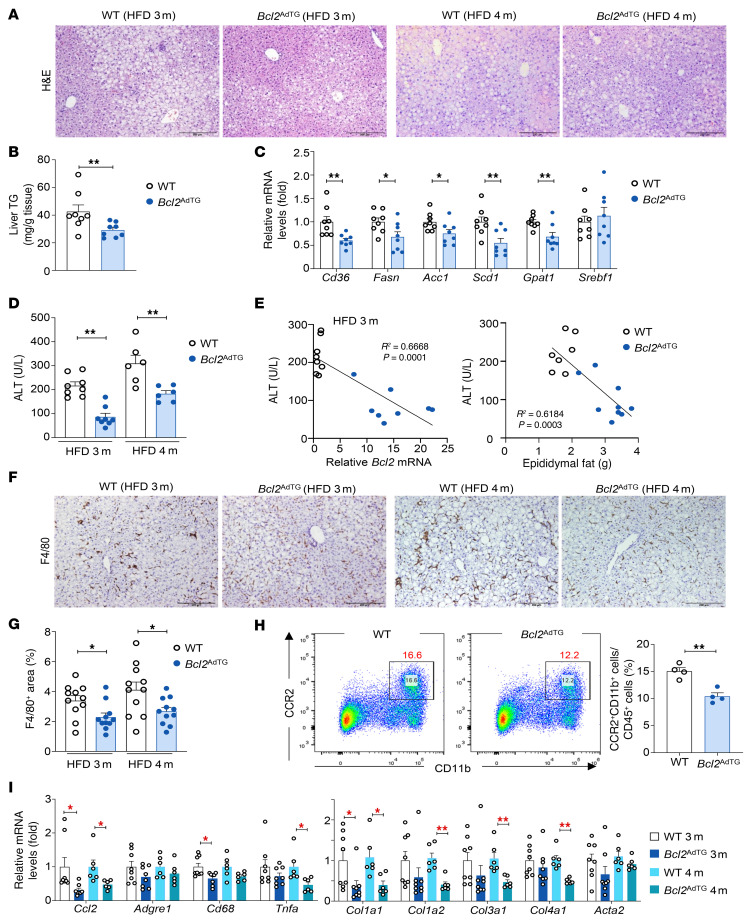
Overexpression of the *Bcl2* gene in adipocytes reduces high-fat diet–induced liver steatosis and MASLD in mice. Male *Bcl2*^AdTG^ mice and WT littermates were fed a high-fat diet (HFD) for 3 or 4 months. (**A**) H&E staining of the paraffin-embedded liver tissues. (**B**) Liver triglyceride levels. (**C**) RT-qPCR analysis of genes involved in fatty acid uptake and lipogenesis. *Apob* was used as a reference gene. (**D**) Serum alanine aminotransferase (ALT) levels. (**E**) The correlation between serum ALT levels and epididymal fat *Bcl2* mRNA levels (left) and epididymal fat weight (right). Solid lines stand for linear regression. (**F** and **G**) IHC analysis of F4/80 in the liver. Scale bars: 200 μm. (**H**) Liver mononuclear cells were subjected to flow cytometry analysis. The values in red represent the percentage of CD11b^+^CCR2^+^ cells among CD45^+^ cells. (**I**) RT-qPCR analysis of inflammatory and fibrotic genes in the liver. Values represent mean ± SEM. Statistical evaluation was performed using Student’s *t* test (**B**–**D**, **G**, and **H**) or 1-way ANOVA with Tukey’s post hoc test for multiple comparisons (**I**). *P < 0.05; **P < 0.01.

We next tested whether adipocyte-specific overexpression of BCL2 affects fat redistribution from adipocytes to hepatocytes during HFD feeding. H&E staining revealed reduced lipid deposition in hepatocytes from *Bcl2*^AdTG^ mice compared with WT mice after HFD feeding (Figure 3A). This reduction in hepatic lipid content was corroborated by lower liver triglyceride levels in *Bcl2*^AdTG^ mice (Figure 3B). Additionally, the expression of genes associated with fatty acid uptake (*Cd36*) and lipogenesis was markedly downregulated in *Bcl2*^AdTG^ mice after 3 months of HFD feeding (Figure 3C).

Next, we examined whether blockade of adipocyte death in *Bcl2*^AdTG^ mice ameliorates liver injury after HFD feeding. As illustrated in Figure 3D, serum ALT levels were lower in *Bcl2*^AdTG^ mice than WT mice after 3 and 4 months of HFD feeding. In addition, a negative correlation was observed between serum ALT and epididymal fat *Bcl2* mRNA levels (Figure 3E). Furthermore, epididymal fat weight, which was higher in *Bcl2*^AdTG^ mice, was negatively correlated with ALT levels (Figure 3E).

F4/80 staining revealed a decrease in macrophage aggregates (Figure 3, F and G), suggesting a reduction in HFD-associated liver inflammation, which was verified by flow cytometric analysis showing a decreased infiltration of CCR2^+^CD11b^+^ macrophages in the liver of *Bcl2*^AdTG^ mice (Figure 3H). Attenuated inflammation was further supported by the downregulation of monocyte chemoattractant CCL2 and macrophage marker CD68 (Figure 3I). In addition, BCL2 overexpression in adipocytes downregulated hepatic fibrogenic genes (Figure 3I) in HFD-fed mice. Notably, the reduction in inflammatory and fibrotic expression due to BCL2 overexpression was observed exclusively in HFD-fed mice but not in chow-fed mice (Supplemental Figure 6).

The role of adipocyte death in fat redistribution from adipocytes to hepatocytes and MASLD progression was further assessed in mice subjected to 1 year of HFD feeding, which induced marked inflammation and fibrosis in the liver. After a year of HFD feeding, *Bcl2*^AdTG^ mice showed greater epididymal fat weight compared with WT mice, and subcutaneous fat weight and body weight were similar between the 2 genotypes (Supplemental Figure 7A). Furthermore, compared with WT mice, *Bcl2*^AdTG^ mice exhibited a decrease in epididymal fat inflammation and a reduction in liver injury, inflammation, and fibrosis (Supplemental Figure 7, B–F).

**Figure 4:**
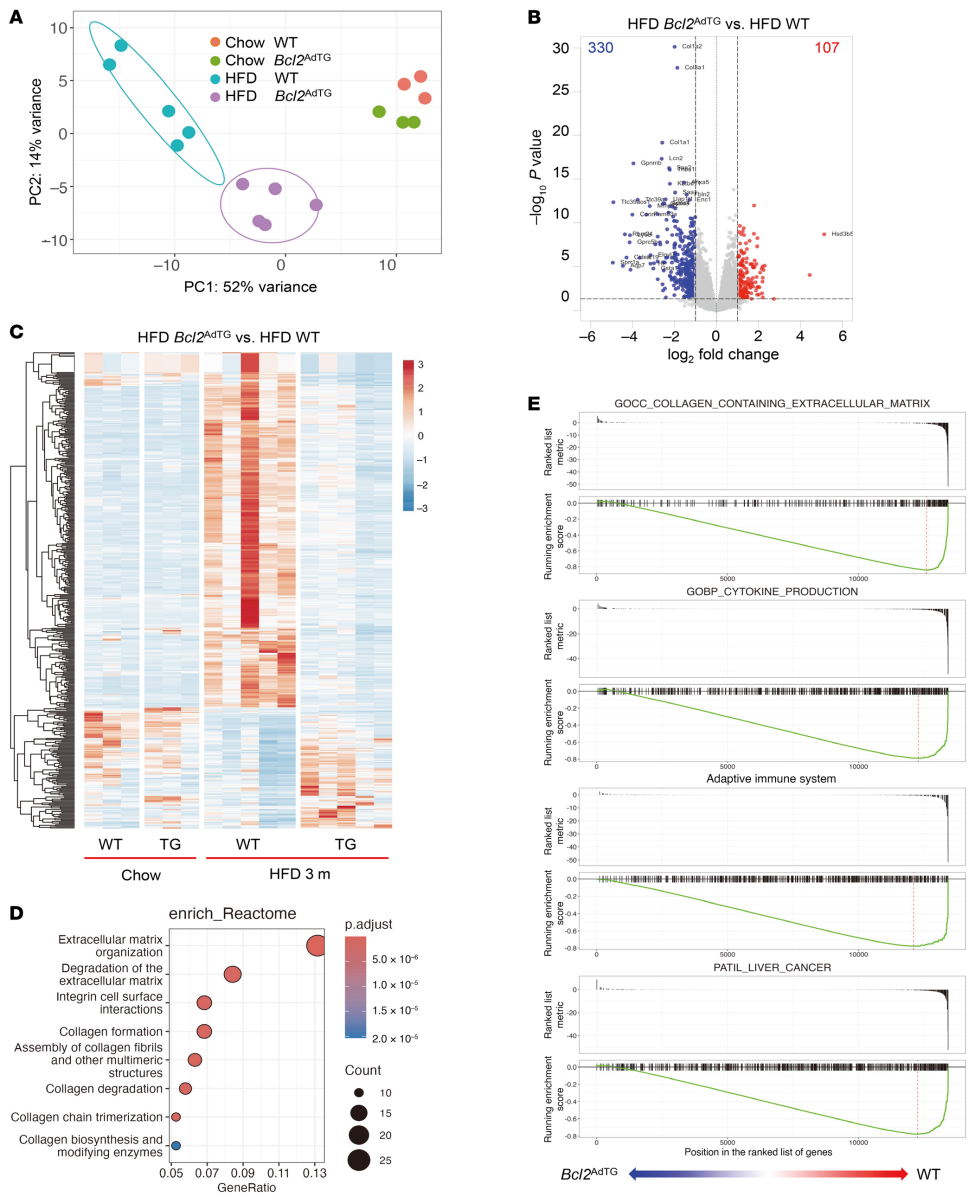
RNA-Seq analysis shows that BCL2 overexpression in adipocytes reverses gene changes in the liver of WT mice fed a high-fat diet. Male *Bcl2*^AdTG^ mice and WT littermates were fed a chow diet or high-fat diet (HFD) for 3 months, and liver tissues were subjected to RNA-Seq analysis. (**A**) Principal component analysis plot shows different gene expression pattern in chow- and HFD-fed *Bcl2*^AdTG^ mice and WT littermates. (**B**) Volcano plot of differentially expressed genes in the livers of HFD-fed *Bcl2*^AdTG^ and WT mice. The *x* axis represents the log_2_ fold change (*Bcl2*^AdTG^ versus WT), and the *y* axis represents the −log10 *P* value. Blue dots indicate downregulated genes (log_2_[fold change] < −1, *P* < 0.05); red dots indicate upregulated genes (log_2_[fold change] > 1, *P* < 0.05). (**C**) Heatmap of differentially expressed genes in the livers of *Bcl2*^AdTG^ and WT mice (|log_2_[fold change]| > 1, *P* < 0.05). (**D**) Reactome pathway analysis of differentially expressed genes in the livers of *Bcl2*^AdTG^ and WT mice (|log_2_[fold change]| > 1, *P* < 0.05). (**E**) Gene set enrichment analysis of collagen- and inflammation-related pathways in the livers of HFD-fed *Bcl2*^AdTG^ versus WT mice.

To further investigate the impact of BCL2 overexpression in adipocytes on liver gene expression, we performed RNA-Seq analysis on liver tissues from *Bcl2*^AdTG^ mice and WT littermates fed either a chow diet or HFD for 3 months. Principal component analysis of the RNA-Seq data showed a distinct clustering of gene expression profiles between chow- and HFD-fed mice, and between *Bcl2*^AdTG^ and WT mice, indicating that *Bcl2*^AdTG^ markedly altered the transcriptional response to HFD feeding (Figure 4A). Differential expression analysis revealed substantial changes in gene expression in the livers of HFD-fed *Bcl2*^AdTG^ mice compared with HFD-fed WT mice, with 107 genes being significantly upregulated and 330 genes downregulated (Figure 4B). Notably, the substantial changes in gene expression observed in the livers of HFD-fed WT mice were largely reversed in HFD-fed *Bcl2*^AdTG^ mice, whose gene expression patterns more closely resembled those of chow-fed mice (Figure 4C). Furthermore, Reactome analysis highlighted that the pathways related to extracellular matrix organization and collagen synthesis, which were upregulated in HFD-fed WT mice, were notably reversed in HFD-fed *Bcl2*^AdTG^ mice (Figure 4D and Supplemental Figure 8A). Gene set enrichment analysis showed that genes involved in cytokine production, immune response, and liver cancer were downregulated in HFD-fed *Bcl2*^AdTG^ mice compared with HFD-fed WT mice (Figure 4E and Supplemental Figure 8, A–D). Collectively, our data suggest a protective role of BCL2 overexpression in adipose tissues against hepatic inflammation and fibrosis.

### HFD feeding induces an enrichment of liver S100A8^+^ macrophages in WT mice, which is reversed in Bcl2^AdTG^ mice.

**Figure 5:**
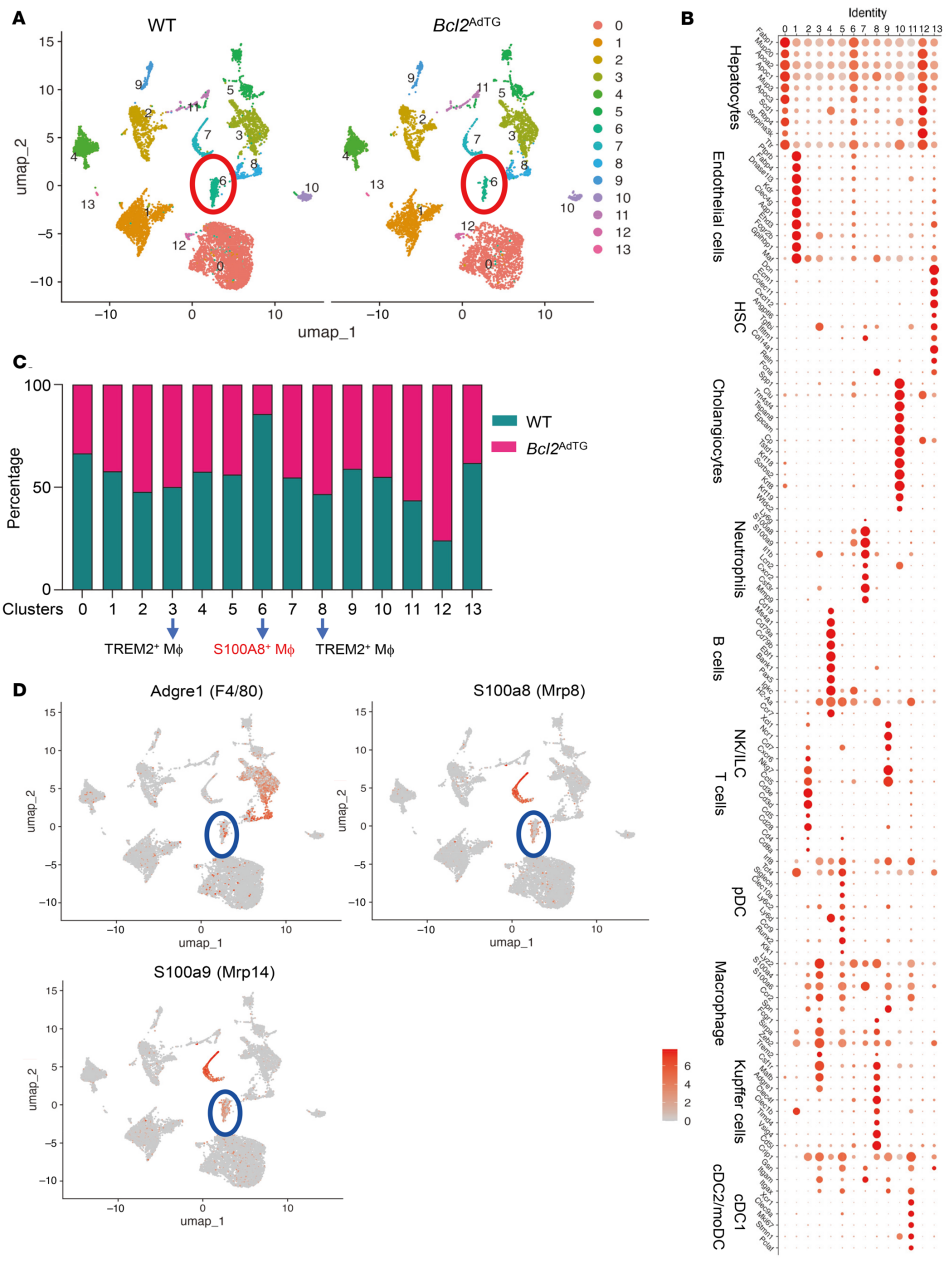
Single-cell RNA-Seq reveals an increase in S100A8^+^ macrophages in the liver of WT mice compared with *Bcl2*^AdTG^ mice. Male *Bcl2*^AdTG^ mice and WT littermates were fed a high-fat diet for 4 months, and single cells were isolated from the livers and subjected to single-cell RNA (scRNA) sequencing analysis. (**A**) scRNA-Seq data of liver cells were analyzed, integrated, and clustered using Seurat v5. Uniform manifold approximation and projection (UMAP) plots displaying all liver cells from *Bcl2*^AdTG^ mice and WT littermates. (**B**) Dot plots of the signature genes defining the specific cell types of each cluster. (**C**) The percentage of cells in each cluster is compared between *Bcl2*^AdTG^ and WT mice. (**D**) Feature plots for the gene expression of *Adgre1* (F4/80), *S100a8* (Mrp8), and *S100a9* (Mrp14) among all the cells. MΦ, macrophages.

To gain deeper insights into the cellular mechanisms by which BCL2 overexpression in adipocytes modulates liver inflammation and fibrosis, we performed scRNA-Seq on liver tissues from *Bcl2*^AdTG^ and WT mice after 4 months of HFD feeding. Our analysis identified 19,134 feature genes expressed across 17,031 cells from WT and *Bcl2*^AdTG^ mouse livers (Figure 5A). Unsupervised clustering of liver cells, performed using 30 principal components at a resolution of 0.1, revealed 14 distinct cell populations (Figure 5A). Liver cell types were annotated based on marker genes (Figure 5B), identifying hepatocytes, endothelial cells, hepatic stellate cells, cholangiocytes, neutrophils, B cells, NK/innate lymphoid cells, T cells, plasmacytoid DCs, macrophages, Kupffer cells, conventional DC type 1 (cDC1), and a mixed population of cDC2/monocyte-derived DCs. Notably, macrophages constituted a major fraction of the immune cell population, and we identified a distinct subset of macrophages expressing *S100a8* (cluster 6, F4/80^+^S100A8^+^), which were lower in the livers of HFD-fed *Bcl2*^AdTG^ mice (118 cells) compared with HFD-fed WT mice (704 cells) (Figure 5, A–D, and Supplemental Figure 9). In contrast, the TREM2^+^ macrophage population (clusters 3 and 8), which is associated with MASLD pathogenesis (41–43), remained unchanged between the 2 groups, with *Bcl2*^AdTG^ mice having 1,171 TREM2^+^ macrophages and WT mice having 1,142 TREM2^+^ macrophages (Figure 5, A–C, and Supplemental Figure 9).

**Figure 6:**
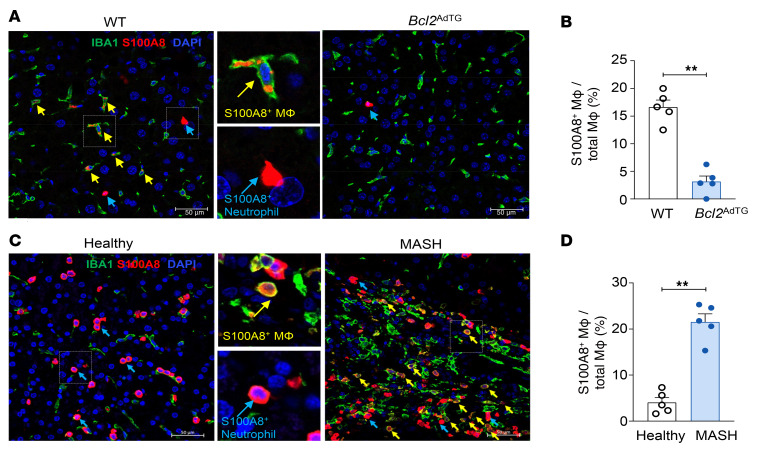
Hepatic S100A8^+^ macrophages are decreased in *Bcl2*^AdTG^ mice and increased in patients with metabolic dysfunction–associated steatohepatitis. (**A**) Male *Bcl2*^AdTG^ mice and WT littermates were fed a high-fat diet for 4 months. IHC analysis of IBA1 and S100A8 in the livers of WT and *Bcl2*^AdTG^ mice. Yellow arrows indicate S100A8^+^ MΦ, and cyan arrows indicate S100A8^+^ neutrophils. Scale bars: 50 μm. (**B**) Percentage of S100A8^+^ MΦ out of total MΦ per field. (**C**) IHC analysis of IBA1 and S100A8 in the livers of healthy individuals and patients with metabolic dysfunction–associated steatohepatitis (MASH). Yellow arrows indicate S100A8^+^ MΦ, and cyan arrows indicate S100A8^+^ neutrophils. (**D**) Percentage of S100A8^+^ MΦ out of total MΦ per field. Scale bars: 50 μm. Values represent mean ± SEM. Statistical evaluation was performed by Student’s *t* test (**P < 0.01). MΦ, macrophages.

The reduction in S100A8^+^ macrophages in *Bcl2*^AdTG^ mice was further corroborated by IHC analysis of ionized calcium-binding adapter molecule 1 (IBA1) (a macrophage marker) and S100A8. Liver sections from HFD-fed *Bcl2*^AdTG^ mice demonstrated a significant reduction in S100A8^+^IBA1^+^ macrophages compared with HFD-fed WT mice (Figure 6, A and B), indicating that HFD-induced adipocyte death induces the increase of hepatic S100A8^+^ macrophages. Consistently, S100A8^+^IBA1^+^ macrophages were significantly increased in liver samples from patients with MASH compared with healthy controls (Figure 6, C and D), reinforcing the relevance of these findings to human disease and implicating S100A8^+^ macrophages as a link between adipocyte death and the pathogenesis of MASLD in patients.

The hepatic S100A8^+^ macrophage population, further validated by flow cytometric analysis using *S100a8-*Cre-ires/GFP mice, was markedly elevated after HFD feeding (Supplemental Figure 10). Consistently, IHC analysis of hepatic S100A8^+^IBA1^+^ macrophages in mice fed an HFD for 1, 2, 3, or 4 months revealed that the number of S100A8^+^IBA1^+^ macrophages progressively increased in proportion to HFD feeding duration (Supplemental Figure 11). Notably, a marked accumulation of S100A8^+^IBA1^+^ macrophages was observed after 4 months of HFD feeding, when adipocyte death is remarkable.

### FFAs released by apoptotic adipocytes induce the elevation of S100A8^+^ macrophages.

**Figure 7:**
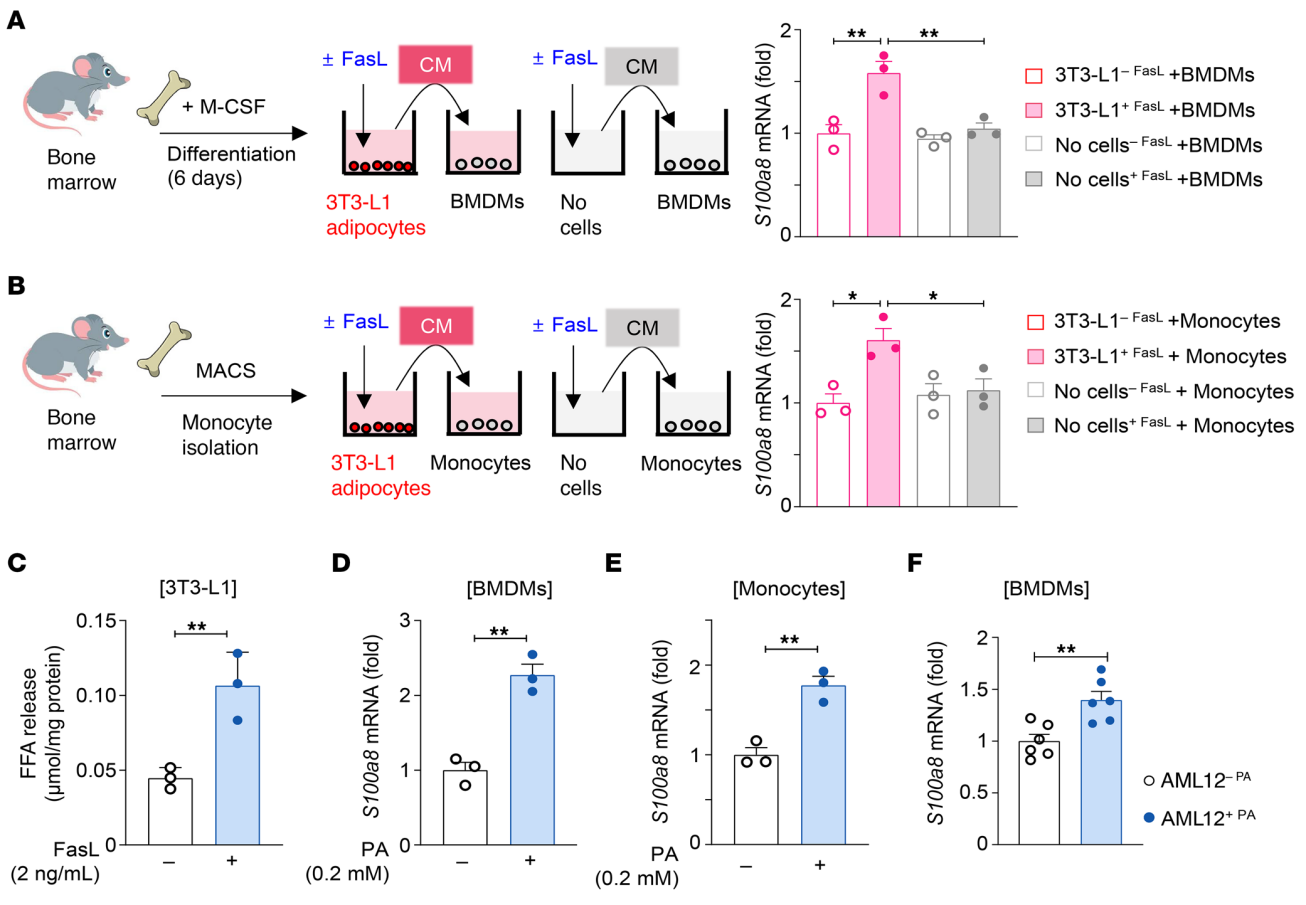
Soluble factors released by apoptotic adipocytes and hepatocytes induce S100A8^+^ macrophages. (**A** and **B**) 3T3-L1 preadipocytes were differentiated into adipocytes and treated with FasL (2 ng/mL) or vehicle for 12 hours to prepare the conditioned media (CM). A control CM was prepared by adding FasL or vehicle to the culture media without 3T3-L1 cells. Mouse BM-derived macrophages (BMDMs) (**A**) or monocytes (**B**) were incubated with either the CM from 3T3-L1 cultures or with the control CM for 24 hours. RNA extracted from monocytes was subjected to RT-qPCR analysis. (**C**) Levels of free fatty acids in the culture supernatant of 3T3-L1 cells treated with vehicle or FasL (2 ng/mL). (**D** and **E**) BM-derived macrophages (**D**) and monocytes (**E**) were treated with palmitic acid (PA, 0.2 mM) or vehicle for 24 hours. RNA was subjected to RT-qPCR analysis. (**F**) AML12 cells were treated with PA (0.6 mM) or vehicle for 6 hours, after which the media were removed. The PA- or vehicle-treated cells were then further cultured in fresh media for an additional 18 hours to obtain the CM. Afterward, BMDMs were treated with the CM for 24 hours. *S100a8* mRNA expression was analyzed by RT-qPCR (*n* = 6; data combined from 2 independent experiments). Values represent mean ± SEM from 3 independent experiments. Statistical evaluation was performed by Student’s *t* test (**C**–**F**) or 1-way ANOVA with Tukey’s post hoc test for multiple comparisons (**A** and **B**). *P < 0.05; **P < 0.01.

We then sought to determine whether adipocyte apoptosis induces S100A8^+^ macrophage elevation. To this end, we first tested whether conditioned media from apoptotic adipocytes can directly induce S100A8 expression in macrophages in vitro by differentiating 3T3-L1 preadipocytes into adipocytes and then treating them with FasL, which induces adipocyte apoptosis (13). Exposure to the conditioned media obtained from FasL-treated, apoptotic 3T3-L1 cells increased *S100a8* mRNA levels in bone marrow-derived macrophages (BMDMs), suggesting that adipocyte death can enhance S100A8 expression in macrophages (Figure 7A). Exposure of macrophages to FasL-containing media without 3T3-L1 cells did not increase *S100a8* mRNA levels, ruling out the possibility that FasL itself induces S100A8 (Figure 7A). Similarly, conditioned media obtained from FasL-treated apoptotic adipocytes elevated *S100a8* mRNA levels in monocytes (Figure 7B). Adipocyte apoptosis caused by FasL was accompanied by a release of FFAs (Figure 7C) (44), leading us to hypothesize that FFAs released from apoptotic adipocytes upregulate *S100a8* mRNA levels in macrophages. Given that treatment with 0.3 mM palmitic acid (PA) induces mild cytotoxicity in BMDMs (45), we tested the effect of PA concentrations below 0.3 mM on the viability of BMDMs. As illustrated in Supplemental Figure 12A, cell counting kit-8 assays revealed that 0.2 mM PA did not affect BMDM viability. Thus, we selected 0.2 mM PA and found that treatment with 0.2 mM PA elevated the transcript levels of *S100a8* in BMDMs and monocytes (Figure 7, D and E). In addition to PA, C18:1 fatty acids, such as oleic acid and elaidic acid, function as major components of adipocyte lipid droplets, and their hepatic levels are elevated in patients with MASLD (46, 47). Thus, we also investigated the effect of oleic acid (C18:1 cis-9), elaidic acid (C18:1 trans-9), and their combination with PA on *S100a8* mRNA levels in BMDMs. As Supplemental Figure 12B shows, although combined treatment with PA, oleic acid (C18:1 cis-9), and elaidic acid (C18:1 trans-9) significantly upregulated *S100a8* mRNA, treatment with either oleic acid or elaidic acid alone failed to induce *S100a8* mRNA expression.

In addition to FFAs, dying adipocytes also release a variety of bioactive components, including extracellular vesicles (EVs) and damage-associated molecular patterns (DAMPs), such as high mobility group box 1 (HMGB1). To investigate whether EVs may also contribute to *S100a8* induction after adipocyte death, we treated BMDMs with EVs isolated from FasL-treated adipocytes. EVs derived from FasL-treated adipocytes significantly induced *S100a8* expression in BMDMs (Supplemental Figure 12C), suggesting that apoptotic adipocyte–derived EVs are able to promote *S100a8* upregulation in macrophages. Treatment with HMGB1 did not alter *S100a8* mRNA levels in BMDMs (Supplemental Figure 12D). Taken together, our findings indicate that adipocyte apoptosis increases the S100A8^+^ macrophage population through FFA release in conjunction with other factors, such as EVs.

Mice fed an HFD exhibit not only adipocyte death but also hepatocyte injury, albeit to a lesser extent. Therefore, we investigated whether upregulation of *S100a8* in macrophages is locally induced by nearby lipid-laden hepatocytes undergoing lipotoxic stress. To address this, we cultured BMDMs in conditioned media from hepatocytes treated with or without PA. Treatment with 0.6 mM PA significantly reduced the viability of AML12 hepatocytes (Supplemental Figure 13), indicating the induction of lipotoxicity. In addition, conditioned media obtained from PA-treated, lipotoxic AML12 hepatocytes upregulated *S100a8* in BMDMs (Figure 7F), suggesting that lipid-laden, lipotoxic hepatocytes could trigger *S100a8* induction in macrophages, presumably by releasing soluble factors, such as FFAs, similar to injured adipocytes.

### S100A8^+^ macrophages increase the expression of CD36 in hepatocytes.

**Figure 8:**
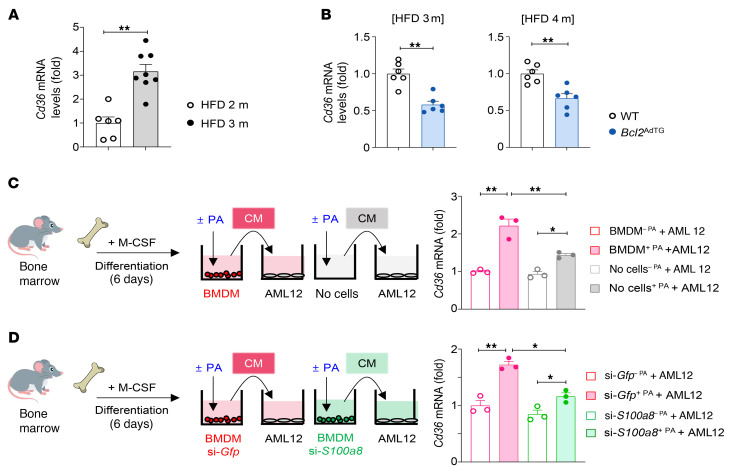
S100A8^+^ macrophages contribute to adipocyte death–associated *Cd36* expression in hepatocytes. (**A**) Male C57BL/6J mice were fed a high-fat diet (HFD) for 2 and 3 months, and mouse livers were subjected to RT-qPCR analysis of *Cd36*. (**B**) RT-qPCR analysis of *Cd36* was performed in the livers of 3-month HFD-fed *Bcl2*^AdTG^ (left), 4-month HFD-fed *Bcl2*^AdTG^ (right), and their WT littermates. (**C**) Mouse BM macrophages were treated with palmitic acid (PA, 0.2 mM) or vehicle for 24 hours to prepare conditioned media (CM). A control CM was prepared by adding PA or vehicle to the culture media without macrophages. AML12 cells were incubated with either the CM obtained from macrophage cultures or with the control CM for 24 hours. RNA extracted from AML12 cells was subjected to RT-qPCR analysis. (**D**) Mouse BM macrophages were isolated and electroporated with siRNAs against *Gfp* or *S100a8*. Then, the macrophages were treated with PA (0.2 mM) or vehicle for 24 hours to obtain the CM. Afterward, AML12 cells were treated with the CM for 24 hours. *Cd36* mRNA expression was analyzed by RT-qPCR. Values represent mean ± SEM. Statistical evaluation was performed by Student’s *t* test (**A** and **B**) or 1-way ANOVA with Tukey’s post hoc test for multiple comparisons (**C** and **D**). *P < 0.05; **P < 0.01.

We next investigated how S100A8^+^ macrophages, increased by adipocyte apoptosis, promote lipid accumulation in hepatocytes. Given that CD36, a key FFA transporter, was downregulated in HFD-fed *Bcl2*^AdTG^ mice compared with HFD-fed WT mice as illustrated in Figure 3C, we hypothesized that S100A8*^+^* macrophages promote CD36 expression in hepatocytes and a reduction of S100A8^+^ macrophages contributes to the downregulation of CD36 in HFD-fed *Bcl2*^AdTG^ mice. As illustrated in Figure 8A, *Cd36* mRNA levels were higher in the livers of mice fed an HFD for 3 months compared with 2 months, indicating that *Cd36* upregulation correlates with increased adipocyte death and hepatic fat accumulation. In contrast, BCL2 overexpression, which mitigated adipocyte death, reduced *Cd36* mRNA levels in the liver of HFD-fed mice (Figure 8B). These results indicate that adipocyte death may induce *Cd36* expression, thereby promoting fatty acid uptake and lipotoxicity during MASLD progression.

Previous studies have reported that PPARγ transactivates *Cd36* (48) and that PPARγ is implicated in MASLD development in both mice and humans (49). To better understand the mechanism underlying the upregulation of CD36 by adipocyte apoptosis, we analyzed the expression of PPARγ and multiple downstream target genes in the livers of *Bcl2*^AdTG^ and WT mice. HFD feeding that induced adipocyte death led to the upregulation of a number of PPARγ target genes (e.g., *Pparg*, *Cd36*, *Cidec*, *Serpine1*, *Ucp2*, *Plin2*, *Fabp4*) in WT mice. Notably, this upregulation was attenuated in *Bcl2*^AdTG^ transgenic mice (Supplemental Figure 14), indicating that suppression of adipocyte apoptosis inhibits hepatic activation of the PPARγ/CD36 axis.

To further test whether S100A8^+^ macrophages contribute to adipocyte death–induced hepatic fat accumulation, we cocultured AML12 hepatocytes with PA-treated macrophages (PA treatment increases S100A8 expression). Conditioned media obtained from PA-treated macrophages significantly elevated *Cd36* expression in AML12 cells (Figure 8C). To rule out the possibility that PA itself included in the conditioned media upregulates *Cd36* in AML12 cells, we compared the effects of conditioned media from PA-treated macrophages with PA itself. Although PA alone elevated *Cd36* transcripts in AML12 cells, conditioned media from PA-treated macrophages caused a much greater increase in *Cd36* mRNA (Figure 8C). Moreover, we conducted an additional experiment to rule out the possibility that residual PA in the conditioned media could influence CD36 expression. In this experiment, BMDMs were treated with PA for 6 hours, after which the PA-containing media were replaced with fresh media, and the PA-treated cells were cultured for 18 hours to collect conditioned media. AML12 cells were then treated with these conditioned media, which were devoid of PA. This approach ensured that any direct effects of PA were eliminated. Notably, CD36 expression remained elevated in response to the conditioned media even after PA removal, further supporting that PA in the conditioned media was not required for CD36 induction in hepatocytes (Supplemental Figure 15). We performed similar experiments using BM-derived monocytes. Consistently, conditioned media obtained from PA-treated monocytes promoted *Cd36* expression in AML12 cells more effectively than PA alone (Supplemental Figure 16). However, mRNA levels of lipogenic genes, such as *Srebf1*, *Fasn*, *Acc1*, and *Scd1*, did not significantly change after coculture (Supplemental Figure 17). We then tested whether S100A8^+^ macrophages are the specific macrophage subset that facilitates *Cd36* induction in hepatocytes by comparing the effects of conditioned media from WT and *S100a8*-silenced BMDMs. Conditioned media from *S100a8*-silenced BMDMs showed a reduced ability to upregulate *Cd36* in AML12 cells compared with controls (Figure 8D).

In Figure 6C, we show that MASH livers from patients exhibited an elevation of the S100A8^+^ macrophage population. To explore the potential link of these S100A8^+^ macrophages and CD36 expression in hepatocytes in MASH livers from patients, we performed coimmunostaining with antibodies against CD36, Heppar1 (a hepatocyte marker), and IBA1 (a macrophage marker), and found that CD36 expression was induced in both hepatocytes and macrophages (Supplemental Figure 18). These results suggest that hepatocyte CD36 expression is associated with the predominance of S100A8^+^ macrophages and MASLD progression in humans.

In addition, we investigated whether S100A8^+^ macrophages affect hepatocyte death or inflammation. Notably, conditioned media from PA-treated BMDMs did not significantly induce cell death or inflammatory gene expression (Supplemental Figure 19), suggesting that an exposure to the macrophage-derived conditioned media alone is not strong enough to induce hepatocyte death or inflammation. Taken together, these results suggest that PA-treated S100A8^+^ macrophages promote *Cd36* expression in hepatocytes, which may in turn contribute to fat accumulation in hepatocytes.

### Conditional deletion of S100a8 in macrophages reduces steatosis in HFD-fed mice.

**Figure 9:**
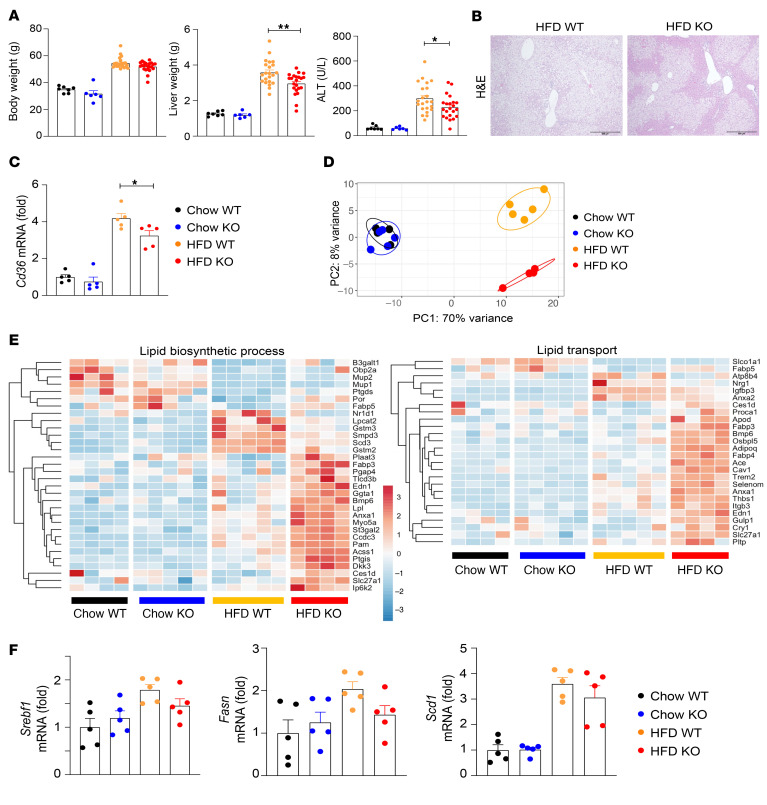
Macrophage-specific deletion of *S100a8* attenuates steatosis development with a downregulation of *Cd36* in the liver of mice fed a high-fat diet. Macrophage-specific *S100a8*-KO (*S100a8^fl/flCx3cr1Cre^*) mice and WT (*S100a8^fl/fl^*) littermates were fed a chow diet or a high-fat diet (HFD) for 3 months. (**A**) Body weight, liver weight, and serum alanine aminotransferase (ALT) levels are shown. (**B**) Paraffin-embedded liver tissues were subjected to H&E staining. (**C**) Liver tissues were subjected to RT-qPCR analysis of *Cd36*. (**D** and **E**) Liver tissues analyzed by RNA-Seq, principal component analysis plot (**D**), and heatmaps of genes involved in lipid biosynthesis (**E**) show different gene expression patterns in chow- and HFD-fed *S100a8^fl/flCx3cr1Cre^* and *S100a8^fl/fl^* littermates. (**F**) Fold changes of RNA-Seq counts for *Srebf1*, *Fasn*, and *Scd1* are shown. Statistical evaluation was performed by 1-way ANOVA with Tukey’s post hoc test for multiple comparisons (**A**, **C**, and **F**). *P < 0.05; **P < 0.01. WT: *S100a8^fl/fl^*; KO: *S100a8^fl/flCx3cr1Cre^*.

To further investigate the role of S100A8^+^ macrophages in MASLD progression, we generated macrophage-specific *S100a8*-deficient mice (*S100a8^fl/flCx3cr1Cre^*) and subjected them to HFD feeding for 4 months. Although S100A8 is expressed by both neutrophils and macrophages, IHC analysis confirmed deletion of S100A8 in macrophages but not in neutrophils from *S100a8^fl/flCx3cr1Cre^* mice (Supplemental Figure 20). Loss of *S100a8* in macrophages led to a reduction in liver injury and steatosis, as evidenced by lower serum ALT levels and reduced liver weight compared with WT controls (Figure 9A). The reduction of lipid droplet formation was clearly observed in *S100a8^fl/flCx3cr1Cre^* mice (Figure 9B). Deletion of *S100a8* in macrophages significantly reduced hepatic *Cd36* mRNA levels in HFD-fed mice (Figure 9C), supporting the involvement of CD36 in mediating hepatic fat accumulation by S100A8^+^ macrophages. Next, we examined whether macrophage-specific deletion of *S100a8* also affects adipose tissues. Our data revealed that adipose tissue weight was not altered by *S100a8* deletion (Supplemental Figure 21A), while fibrotic changes in adipose tissues were attenuated in *S100a8^fl/flCx3cr1Cre^* mice (Supplemental Figure 21B).

To determine whether the reduction in hepatic fat accumulation and ALT levels is attributable to a unique property inherent to macrophages, we isolated S100A8^+^ and S100A8^–^ macrophage populations from HFD-fed *S100a8*-Cre-ires/GFP mice using FACS-assisted sorting and analyzed the expression of genes associated with inflammation, fibrosis, and lipid handling. Notably, *Apoe* expression was elevated in S100A8^+^ macrophages, suggesting a potential role in promoting the uptake of cholesterol- and triglyceride-rich lipoproteins (Supplemental Figure 22). In addition, *C1qa*, *C1qb*, and *C1qc* were also upregulated (Supplemental Figure 22). These genes encode subunits of C1q complex, a key component of the classical complement pathway that facilitates efferocytosis, a phagocytic clearance of apoptotic cells, thereby modulating inflammation and tissue homeostasis. In contrast, the expression of inflammatory mediators, such as *Il1b*, *Tnfa*, *Icam1*, *Cxcl1*, *Cxcl2*, *Anxa2*, and *Trem2*, were not significantly different between the 2 macrophage subsets. These findings suggest that S100A8^+^ macrophages possess distinct features related to lipid metabolism and efferocytosis rather than classical proinflammatory activation.

To determine the additional mediators that may attenuate steatosis in *S100a8*-deficient mice, we performed RNA-Seq analysis of liver tissues from *S100a8^fl/flCx3cr1Cre^* and *S100a8^fl/fl^* littermates on either a chow diet or HFD, which revealed distinct gene expression patterns between *S100a8^fl/flCx3cr1Cre^* mice and *S100a8^fl/fl^* littermates on the HFD (Figure 9D and Supplemental Figure 23A). However, genes involved in lipid biosynthesis were not significantly reduced in HFD-fed *S100a8*-deficient mice (Figure 9E and Supplemental Figure 23B). In particular, the master regulator of lipogenesis, *Srebf1*, and its downstream lipogenic genes (e.g., *Fasn*, *Scd1*) were not significantly different between the 2 genotypes (Figure 9F).

Deletion of *S100a8* in macrophages may result in a compensatory increase in other macrophage subsets. Previous studies have identified several populations of monocyte-derived macrophages associated with liver disease, including lipid-associated macrophages (50), nonalcoholic steatohepatitis–associated macrophages (51), and scar-associated macrophages (52). Additionally, we have previously reported a subset of necrotic lesion–associated macrophages involved in concanavalin A-induced liver necrosis lesion resolution (53). The gene signatures associated with these macrophages were also analyzed in the transcriptomic data from HFD-fed WT and HFD-fed *S100a8^fl/flCx3cr1Cre^* mice, and we found that the majority of signature genes associated with these macrophage populations were similarly upregulated in the livers from both WT and *S100a8^fl/flCx3cr1Cre^* mice after HFD feeding (Supplemental Figure 24). This suggests that various disease-associated macrophage populations share overlapping gene signatures and that both HFD-fed WT and HFD-fed *S100a8*-deficient mice recruit inflammatory macrophages to the liver. Several of these genes, such as *Trem2*, *Gpnmb*, *Fabp4*, and *Fabp5*, were more highly expressed in *S100a8*-deficient mice under HFD conditions (Figure 9E and Supplemental Figure 24). Notably, TREM2^+^ macrophages have been demonstrated to play a protective role against MASLD pathogenesis (42), and *Trem2* expression was elevated in the liver of *S100a8*-deficient mice (Figure 9E), suggesting an increased recruitment of the reparative macrophage subset. However, the upregulation of several inflammatory and lipid-associated genes in *S100a8*-deficient mice indicates a complex regulatory mechanism governing macrophage recruitment after *S100a8* deletion.

### Deletion of S100a8 in macrophages upregulates the production of CCN3, which suppresses CD36 expression in hepatocytes.

**Figure 10:**
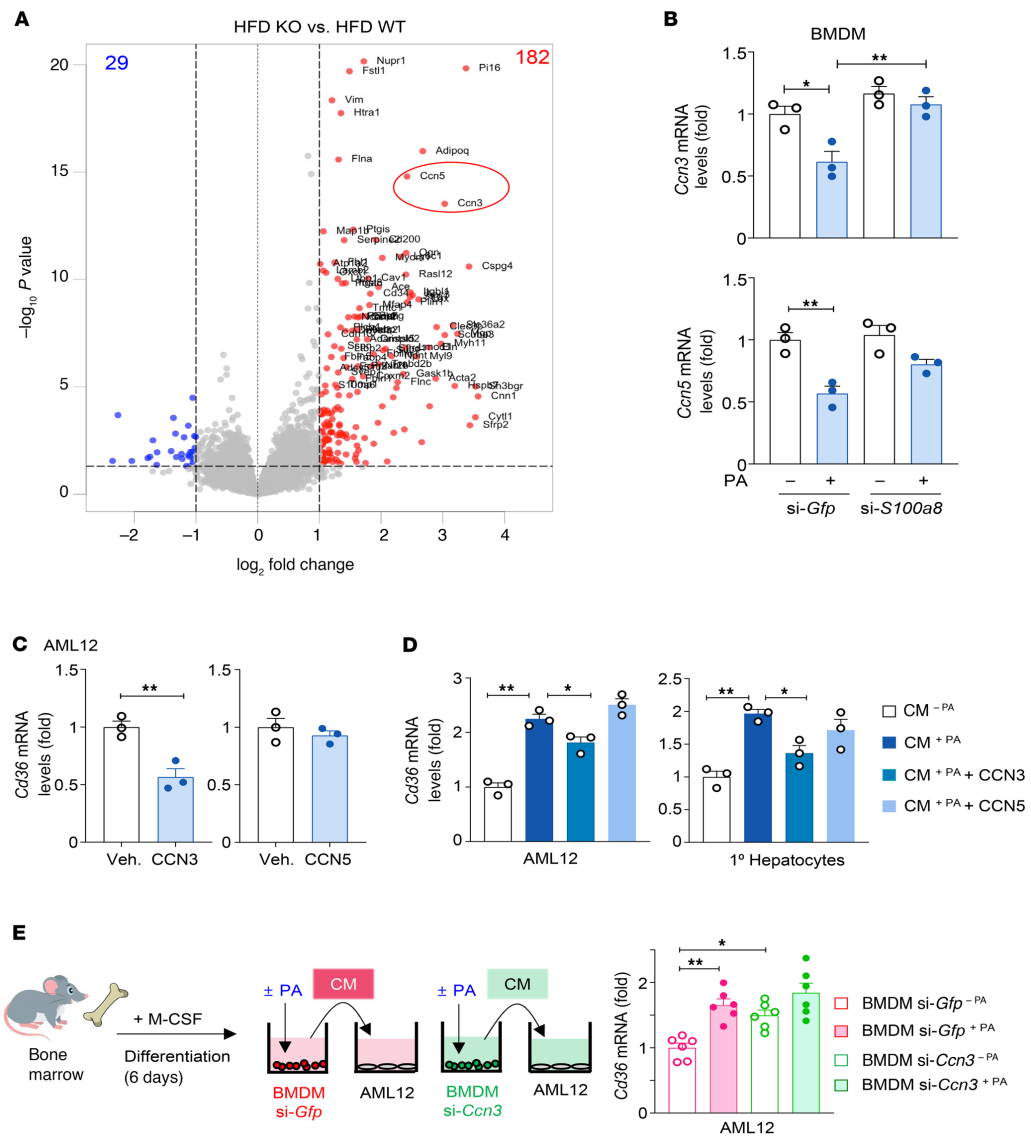
S100A8^+^ macrophages contribute to hepatocyte lipid accumulation by downregulating CCN3. (**A**) Macrophage-specific *S100a8*-KO (*S100a8^fl/flCx3cr1Cre^*) mice and WT (*S100a8^fl/fl^*) littermates were fed a chow diet or high-fat diet (HFD) for 3 months. Liver tissues were analyzed by RNA-Seq, and a volcano plot of differentially expressed genes in the livers of HFD-fed *S100a8^fl/flCx3cr1Cre^* versus *S100a8^fl/fl^* littermates is shown. Blue dots (log_2_[fold change] < –1, *P* < 0.05), red dots (log_2_[fold change] > 1, *P* < 0.05). (**B**) Mouse BM macrophages were isolated and electroporated with siRNAs against *Gfp* or *S100a8*. Then, cells were treated with PA (0.2 mM) or vehicle for 24 hours, and RNA was subjected to RT-qPCR analysis. (**C**) AML12 cells were treated with a recombinant CCN3 or CCN5 (100 ng/mL) for 6 hours, and RNA was subjected to RT-qPCR analysis. (**D**) AML12 cells and primary hepatocytes were cultured in conditioned media (CM) obtained from PA-treated macrophages or vehicle-treated macrophages. Concomitantly, the cells were treated with a recombinant CCN3 or CCN5 for 6 hours, and RNA was subjected to RT-qPCR analysis. (**E**) Mouse BM-derived macrophages were electroporated with siRNAs against *Gfp* or *Ccn3*. Then, cells were treated with PA (0.2 mM) or vehicle for 24 hours, and RNA was subjected to RT-qPCR analysis. Values represent mean ± SEM. Statistical evaluation was performed by Student’s *t* test (**C**) or 1-way ANOVA with Tukey’s post hoc test for multiple comparisons (**B**, **D**, and **E**). *P < 0.05; **P < 0.01. WT: *S100a8^fl/fl^*; KO: *S100a8^fl/flCx3cr1Cre^*.

To elucidate the factors that mediate hepatocyte *Cd36* induction by S100A8^+^ macrophages, we further analyzed our RNA-Seq data and identified a variety of differentially expressed genes, including 29 genes downregulated and 182 genes upregulated in HFD-fed *S100a8^fl/flCx3cr1Cre^* mice compared with their *S100a8^fl/fl^* littermates (Figure 10A). Among the differentially expressed genes, we aimed to identify the soluble factors produced by macrophages that may affect *Cd36* expression in hepatocytes. In this regard, CCN3 and CCN5 were found to be the most highly upregulated soluble factors (Figure 10A).

Previous studies have demonstrated that CCN3 and CCN5 are produced by macrophages (54–56), and myeloid-specific *Ccn3* deletion elevates *Cd36* expression and increases steatosis in the liver, although the regulatory mechanisms were not elucidated (37). In addition, *Cd36* expression was elevated in *Ccn5*-deficient mice fed an HFD (57). Thus, we hypothesized that PA-stimulated S100A8^+^ macrophages downregulate CCN3 and/or CCN5, relieving their inhibitory effect on CD36 and facilitating hepatocyte fat accumulation. To test this hypothesis, we performed several in vitro experiments. First, we found that PA treatment downregulated the expression of *Ccn3* and *Ccn5* in control macrophages, but such reduction was not observed in *S100a8*-KD macrophages (Figure 10B). The S100A8-dependent suppression of CCN3 was further supported by the observation that *Ccn3* mRNA levels were significantly lower in S100A8^+^ macrophages than in S100A8^–^ macrophages (Supplemental Figure 22). Second, treatment with CCN3 reduced *Cd36* expression in AML12 hepatocytes, whereas CCN5 failed to reduce *Cd36* expression (Figure 10C). CCN3 treatment also led to a reduction in FFA uptake in AML12 hepatocytes (Supplemental Figure 25). Third, conditioned media from PA-treated macrophages increased *Cd36* expression, which was reversed by adding CCN3, but not by CCN5, in AML12 cells and primary mouse hepatocytes (Figure 10D). Furthermore, conditioned media from *Ccn3*-silenced BMDMs elevated *Cd36* mRNA levels in the absence of PA, where basal *Ccn3* expression is high and *Cd36* expression is low. In contrast, in the presence of PA, where *Ccn3* expression is already reduced, additional *Ccn3* KD did not significantly enhance *Cd36* expression (Figure 10E). These results indicate that S100A8^+^ macrophages stimulate *Cd36* expression in hepatocytes by reducing the release of CCN3, thereby facilitating lipid accumulation in hepatocytes. Treatment with recombinant CCN3 reduced PPARγ expression in primary hepatocytes (Supplemental Figure 26), suggesting a possibility that CCN3 inhibits CD36 through PPARγ inhibition.

To further investigate the S100A8-dependent regulatory network of PPARγ, we examined whether S100A8^+^ macrophages induce PPARγ target genes beyond CD36. Transcriptomic analysis revealed that PPARγ target genes were mostly upregulated in response to HFD feeding, whereas macrophage-specific deletion of *S100a8* did not downregulate PPARγ target genes in HFD-fed mice (Supplemental Figure 27). These findings suggest that the regulation of PPARγ target genes is complex and not exclusively dependent on PPARγ, requiring the interplay of multiple factors.

## Discussion

**Figure 11:**
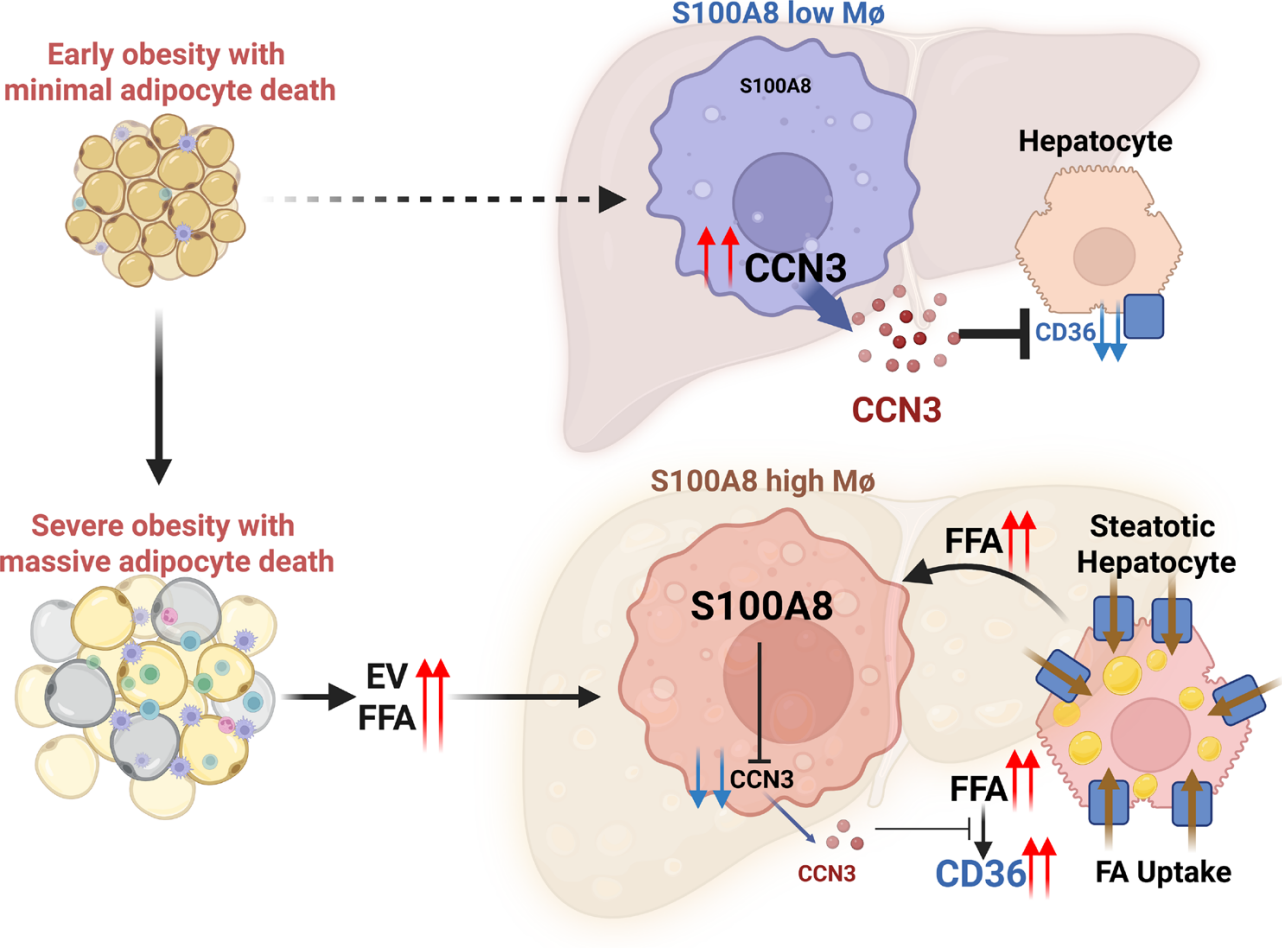
An illustration depicting how adipocyte death triggers hepatic lipid accumulation by activating S100A8^+^ macrophages. Adipocyte death in obesity elevates the S100A8^+^ macrophage population in steatotic liver, and such S100A8 induction is partially mediated via fatty acids and extracellular vesicles released by injured adipocytes and fatty acids from injured steatotic hepatocytes. S100A8^+^ macrophages direct hepatocytes to store lipids by inducing CD36 in hepatocytes, which is in part mediated via the downregulation of CCN3, a macrophage-derived soluble factor that inhibits CD36.

Although mice show immediate increases in body weight and adipose tissue mass upon exposure to an obesogenic HFD, hepatic fat accumulation exhibits a delayed onset. Notably, epididymal adipose atrophy and adipocyte death emerge after 3 months of HFD feeding, which coincides with the onset of hepatic fat accumulation, suggesting that adipocyte death may drive the redistribution of fat from epididymal adipose tissue to the liver. Building upon this observation, our current study provides the evidence linking adipocyte death to steatosis development in mice and identifies the underlying mechanisms. In detail, we have observed that adipocyte death increases the S100A8^+^ macrophage population in steatotic liver, and such S100A8 induction is likely mediated via fatty acids and EVs released by injured adipocytes. S100A8^+^ macrophages direct hepatocytes to store lipids by inducing CD36 in hepatocytes, which is in part mediated via the downregulation of CCN3, a macrophage-derived soluble factor that inhibits CD36 (Figure 11).

Adipocyte death is often observed in mice and humans with obesity (8, 9). With regard to apoptosis, it has been demonstrated that apoptosis-related proteins, such as caspases, are upregulated in the adipose tissues of obese individuals, while antiapoptotic factors, such as BCL2, are downregulated (15). The current study employed a genetically modified mouse model that overexpresses BCL2 in adipocytes and found that blockade of adipocyte death via the overexpression of BCL2 mitigated hepatic lipid accumulation, tissue injury, and inflammation induced by HFD feeding. These results clearly indicate that adipocyte apoptosis contributes to lipid redistribution from adipocytes to hepatocytes and MASLD in mice.

The current study yielded several findings suggesting that liver-infiltrating macrophages mediate adipocyte death–associated fat redistribution from adipocytes to hepatocytes and lipotoxicity during MASLD progression. Preventing adipocyte apoptosis by BCL2 overexpression reduced hepatic macrophage infiltration in HFD-fed mice. We have previously reported that infiltration and activation of macrophages are implicated in liver injury and inflammation caused by acute adipocyte death (58). Expanding on these findings, we identified S100A8^+^ macrophages, in particular, as a potential player in mediating adipocyte death–induced hepatic fat accumulation and MASLD development. Four months of HFD feeding, which induces adipocyte death, substantially elevated the S100A8^+^ macrophage population in the liver of mice. In addition, adipocyte-specific BCL2 overexpression clearly reduced the S100A8^+^ macrophage population in the liver. We also gained in vitro evidence suggesting that adipocyte apoptosis promotes S100A8 expression in macrophages, potentially mediated by FFAs and EVs released by apoptotic adipocytes.

S100A8 is highly expressed in myeloid cells, such as neutrophils and macrophages (59). S100A8 performs both intracellular and extracellular functions (17, 59). Intracellularly, S100A8 is involved in cytoskeleton modulation, which is essential for processes such as cell migration, phagocytosis, and exocytosis. In addition, S100A8 regulates ROS production, as well as the differentiation and proliferation of myeloid cells (60). Extracellularly, S100A8 released by myeloid cells facilitates leukocyte recruitment and stimulates cytokine production by inflammatory cells (17). We previously demonstrated that S100A8 released by neutrophils in adipose tissue contributes to the progression of MASLD (22). Regarding the specific role of S100A8^+^ macrophages in liver diseases, Liu et al. reported that S100A8 promotes liver fibrosis in a carbon tetrachloride–induced mouse model by activating the TLR4/NF-κB signaling pathway in macrophages and inducing NLRP3 inflammasome-dependent pyroptosis (28). However, there was no conclusive in vivo evidence supporting these reported functions of S100A8 due to the lack of *S100a8*-KO mice, given that global deletion of the *S100a8* gene is embryonic lethal (23), and the role of S100A8^+^ macrophages in hepatic fat accumulation and the progression of steatosis remains unclear.

To address the knowledge gap regarding S100A8^+^ macrophage–derived mediators that induce fat accumulation in hepatocytes, we generated macrophage-specific *S100a8*-KO mice to study the role of macrophage S100A8 in promoting steatosis after HFD feeding, and we identified the ability of S100A8^+^ macrophages to alleviate CCN3-induced CD36 suppression as a potential mechanism contributing to hepatocyte fat accumulation. Previous studies have reported that CD36 expression is increased in CCN3-KO mice (37); however, the direct effect of CCN3 on CD36 remains unexplored. In this study, we found that CCN3 treatment reduced *Cd36* mRNA levels in AML12 hepatocytes. Furthermore, PA-treated S100A8^+^ macrophages significantly increased CD36 expression in AML12 cells, which was reversed by concomitant treatment with CCN3. Despite these observations, the precise mechanism by which CCN3 suppresses CD36 remains unclear. Martinerie et al. have reported that *Ccn3* deletion upregulates PPARγ coactivator-1 alpha (PGC-1α) (61). Because PGC-1α serves as a coactivator of PPARγ, a transcriptional regulator of CD36 (48), further studies are needed to determine whether CCN3 inhibits CD36 via suppression of the PPARγ signaling pathway. In addition, CCN3 has been identified as a noncanonical ligand for NOTCH1, capable of activating NOTCH1 signaling (62). Exploring the involvement of these signaling pathways could provide valuable insights into the mechanisms underlying CCN3-mediated downregulation of CD36 and the inhibition of fat accumulation in hepatocytes. The current study provides evidence that elevated expression of CCN3 in the liver of macrophage-specific *S100a8*-KO mice inhibits CD36 expression and suppresses hepatic steatosis. In addition, the decrease in steatosis itself may also secondarily suppress CD36 expression by suppressing PPARγ, a transcriptional activator of CD36. Further investigation into this possibility will be essential to fully elucidate the molecular mechanisms underlying CD36 downregulation and steatosis attenuation in *S100a8*-KO mice.

The current study highlighted S100A8^+^ macrophages as the potential mediator connecting epididymal adipocyte death to hepatic lipid accumulation; however, the complete mechanistic links remain to be fully established. Our in vitro studies demonstrated that S100A8 expression was elevated in macrophages by treatment with PA and in the EVs and conditioned media obtained from apoptotic adipocytes. Previous studies have identified transcription factors, including C/EBP and activator protein 1 (AP-1), as upstream regulators of S100A8 expression (63). Thus, it would be interesting to test whether PA or EVs from apoptotic adipocytes upregulate S100A8 expression by regulating C/EBP and AP-1. In addition, there are several possibilities by which FFAs increase the S100A8^+^ macrophage population in the liver. First, adipocyte death selectively enhances the recruitment of monocytes/macrophages with high S100A8 expression to the liver; second, FFAs promote S100A8 expression in circulating monocytes prior to their migration into the liver, thereby increasing the overall population of S100A8^+^ monocytes in the circulation; third, FFAs directly upregulate S100A8 expression in liver-resident macrophages. Detailed investigations incorporating markers that distinguish monocyte-derived macrophages, resident macrophages, and other macrophage subsets will be essential to accurately identify the specific origins of S100A8^+^ macrophages in the liver and their role in the pathogenesis of hepatic steatosis.

Although the current study primarily focused on the role of PAs in promoting adipocyte death–associated induction of S100A8 in macrophages, it is likely that various factors released from dying adipocytes, such as EVs and DAMPs, also contribute to the regulation of S100A8 expression. Indeed, we also found that the treatment of macrophages with EVs derived from dying adipocytes significantly upregulated S100A8 expression in macrophages. In contrast, treatment with recombinant HMGB1 did not increase S100A8 levels, suggesting that HMGB1 alone is insufficient. Nevertheless, the involvement of other DAMPs, such as heat shock proteins or extracellular DNA, in modulating S100A8 expression cannot be excluded.

### Clinical relevance of the current study.

The traditional “two-hit” theory of MASLD pathogenesis posits a sequential model in which hepatic fat accumulation, lipotoxicity, and inflammation act as the primary drivers of disease progression. However, the limited success of therapeutic approaches targeting individual components of this cascade has prompted a shift toward the “multiple-hit” theory (64). This updated concept emphasizes that various factors such as gut microbiome, adipose tissue dysfunction, dietary influences, and genetic predispositions collectively contribute to MASLD development. In this context, our study focuses on interorgan crosstalk and highlights the critical role of adipocyte death and S100A8^+^ macrophages in mediating lipid redistribution from adipose tissue to the liver during MASLD development in mice. Although we were unable to directly test this notion in patients due to the difficulty of obtaining paired liver and adipose tissues from the same patients, several lines of evidence support a link between adipocyte death and steatosis/MASLD in patients via the activation of S100A8^+^ macrophages. First, adipose tissues from obese individuals exhibit pronounced adipocyte death, enhanced expression of caspase-3, and reduced expression of antiapoptotic factors such as BCL2 (15). Second, in the current study, we detected an increased population of S100A8^+^ macrophages in MASLD livers from patients, which was accompanied by upregulated hepatocyte CD36 expression in these patients. Moreover, a recent study by Ojeda et al. reported that individuals with hepatic steatosis exhibit elevated levels of PA included in VLDLs produced by hepatocytes, reflecting PA accumulation in the liver (65). Given that PA induces S100A8 expression as revealed by the current study, this finding further supports the clinical relevance of S100A8^+^ macrophage induction in steatosis. Collectively, these findings underscore a pathogenic axis linking adipocyte death, PA accumulation, S100A8^+^ macrophage activation, and CD36-mediated hepatic lipid uptake. The current study highlights the need for future clinical investigations to validate this axis and its therapeutic potential in patients with MASLD. Finally, we also acknowledge that the physiological and pathological differences between mice and humans may limit the translation of our findings to humans. For example, significant anatomical differences in adipose tissue exist between mice and humans. In addition, mouse MASLD models do not fully replicate the pathogenesis of MASLD in humans. Although we observed an increase in S100A8^+^ macrophages in human MASLD, similar to mice, interspecies differences in immune responses that are unidentified could hinder our understanding of the interactions between adipocytes and hepatocytes in humans. Further experiments focused on the interspecies differences are essential to address these limitations and clarify the mechanistic pathways involved in patients with MASLD. Moreover, future clinical studies exploring sex differences in adipocyte death and MASLD in patients would provide valuable insights and further enhance the translational relevance of our findings into clinical practice and treatment.

## Methods

Further information can be found in the Supplemental Methods.

### Sex as a biological variable.

Most animal experiments in this study were conducted using male mice, as female mice are relatively resistant to HFD-induced obesity. However, to compare adipose tissue characteristics between sexes under HFD conditions, an additional experiment was performed using female mice, as shown in Supplemental Figure 3.

### Mice.

C57BL/6J, *Adipoq*-Cre (Jax 028020), *S100a8*-Cre-ires/GFP (Jax 021614), and *Cx3cr1*-Cre (Jax 025524) mice were obtained from The Jackson Laboratory. Cre-inducible *Bcl2*-transgenic mice were described previously (66) and were used to generate adipocyte-specific *Bcl2*-transgenic mice (*Bcl2*^AdTG^) by crossing with *Adipoq*-Cre mice. *S100a8^fl/fl^* mice were described previously (67) and were used to generate macrophage-specific *S100a8*-deficient mice (*S100a8^fl/flCx3cr1Cre^*) by crossing with *Cx3cr1*-Cre mice. For HFD feeding studies, mice aged 8 weeks were placed on an HFD (60% kcal fat, D12492; Research Diets) or a chow diet (10% kcal fat) as a control. All animals received humane care in accordance with the *Guide for the Care and Use of Laboratory Animals* (National Academies Press, 2011), and all animal experiments were approved by the NIAAA IACUC (LLD-BG-1) and Pusan National University IACUC (PNU-2025-0575).

### Bulk RNA-Seq.

Total RNA was extracted from whole livers using TRIzol (Alkali Scientific). Liver RNA-Seq was performed using Illumina HiSeq. Sequence reads were analyzed on the NIH HPC Biowulf cluster (http://hpc.nih.gov) using the updated applications of Trimmomatic, Hisat2, and FeatureCounts. Gene raw counts were normalized, and differential expression analysis was performed with the R package DESeq2. R packages clusterProfiler and pheatmap were used to perform pathway analysis and create the heatmap plots.

### ScRNA-Seq.

The liver samples were homogenized with a gentleMACS Dissociator (Miltenyi Biotec), filtered through 70 μm cell strainers (Falcon 352350), and then centrifuged at 400*g* through a Percoll density gradient (40% and 70%). The cells were loaded in the lanes using 10x Genomics Chromium Next GEM Single Cell 3′ Kit v3.1 with a single capture lane per sample targeting recovery of 8,000 cells. All subsequent steps of library preparation and quality control were performed according to the 10x Genomics Chromium Single Cell 3′ Reagent Kits User Guide. Sequencing was performed on an Illumina NovaSeq S4. The FASTQ data files were aligned to the 10x Genomics mouse reference sequence (refdata-gex-mm10-2020-A) using 10x Genomics Cell Ranger on the NIH HPC Biowulf cluster. The Seurat package was used to further analyze the scRNA-Seq data (68). After removing dying cells with high mitochondria genes, doublets, and cells with low quality, unique molecular identifier counts for each gene were normalized, and highly variable genes were identified via the FindVariableFeatures function. The data were integrated with the IntegrateData function utilizing the RPCA method of the Seurat package. The first 30 principal components and the resolution 0.1 were used for clustering via FindNeighbors and FindClusters functions. Differential gene expression was assessed using the FindMarker function. The UMAP plots, dot plot, feature plots, and heatmaps were generated by R.

### Statistics.

Data are expressed as mean ± SEM and were analyzed using GraphPad Prism software (version 7.0a). To compare values obtained from 2 groups, Student’s 2-tailed *t* test was performed. Data from multiple groups were compared with 1-way ANOVA followed by Tukey’s post hoc test. *P* values of less than 0.05 were considered significant.

### Study approval.

The NIAAA IACUC approved all animal procedures conducted at the NIAAA under protocol number LLD-BG-1. The Pusan National University IACUC approved all animal procedures conducted at Pusan National University under PNU-2025-0575.

### Data availability.

Bulk RNA and scRNA-Seq data are deposited in the NCBI’s Gene Expression Omnibus with the identifiers GSE285614 and GSE285615, respectively. Values for all data points in graphs are reported in the Supporting Data Values file.

## Author contributions

YG was listed first because they designed and conducted mouse model experiments, performed bulk and scRNA-Seq analysis, and wrote the paper. YK performed in vitro experiments, analyzed the data, and wrote the paper. YW, XX, SJK, and TY helped with mouse model experiments. YEC performed in vitro experiments. DF conducted immunofluorescence staining and flow cytometry experiments. SH designed and conducted mouse model experiments as a postdoctoral fellow at the NIAAA from 2017 to 2021, performed in vitro experiments in his own laboratory at Pusan National University from 2021 to 2025, supervised the project, and wrote the paper. BG supervised the whole project and wrote the paper. All authors approved the final manuscript.

## Funding support

This work is the result of NIH funding, in whole or in part, and is subject to the NIH Public Access Policy. Through acceptance of this federal funding, the NIH has been given a right to make the work publicly available in PubMed Central.

- Intramural program of NIAAA, NIH (AA000369 and AA000368) (to BG).
- National Research Foundation of Korea grant (Ministry of Science and Information-Communication Technology), 2022R1C1C1003563, to SH).

## Supplementary Material

Supplemental data

Unedited blot and gel images

Supporting data values

## Version 1 11/03/2025

Electronic publication
