# Supplementary material for: Adipocyte death promotes hepatic infiltration of S100A8+ macrophages and steatotic liver disease progression in mice
Source: J Clin Invest. 2025 Nov 3;135(21):e190635. doi: 10.1172/JCI190635 (PMC12578405; doi:10.1172/JCI190635)
Supplement: Supplemental data [file jci-135-190635-s212.pdf]

<u>**Supplementary Information**</u>

Adipocyte death promotes hepatic infiltration of S100A8$^+$ macrophages and steatotic liver disease progression in mice.

Yukun Guan,[1,#] Yeonsoo Kim,[2 #] Yang Wang,[1] Ye Eun Cho,[2] Xiaogang Xiang,[1] Seung-Jin Kim,[1] Tiantian Yao,[1] Dechun Feng,[1] Seonghwan Hwang,[1, 2] Bin Gao[1]

[1]Laboratory of Liver Diseases, National Institute on Alcohol Abuse and Alcoholism, National Institutes of Health, Bethesda, MD, USA; [2]College of Pharmacy and Research Institute for Drug Development, Pusan National University, Busan, Republic of Korea

Supplementary Methods

Cell culture

AML12 mouse hepatocyte cell line (ATCC, Manassas, VA) was cultured in a 1:1 mixture of DMEM and Ham's F12 medium with 0.005 mg/mL insulin, 0.005 mg/mL transferrin, 5 ng/mL selenium, 40 ng/mL dexamethasone, 10% fetal bovine serum (FBS), and 1% penicillin-streptomycin. 3T3-L1 preadipocyte cell line was obtained from ATCC (Manassas, VA). The cells were maintained in DMEM supplemented with 10% FBS, 1% penicillin-streptomycin at 37 ℃ in a humidified atmosphere with 5% CO_2. Differentiation into adipocytes was induced by adding 3-isobutyl-1-methylxanthine (0.5 mM), dexamethasone (0.25 μM), insulin (1 μg/mL), and rosiglitazone (2 μM) in the culture media as previously described (1).

Measurement of hepatic triglyceride content

Approximately 50 mg of the frozen liver tissue was used for determination of triglyceride content with Triglyceride Colorimetric Assay Kit (Cayman Chemical, Ann Arbor, MI), according to the assay protocol.

Measurement of serum ALT

Serum ALT levels were determined from the blood drawn from the mouse retro-orbital plexus using Catalyst Dx Chemistry Analyzer (IDEXX Laboratories, Westbrook, ME) according to the manufacturer's instructions.

Isolation of mouse bone marrow-derived monocytes

Mouse bone marrow was collected from femur and tibia and passed through a 70 µm cell strainer in PBS, and the cell suspension was centrifuged at 300×g for 5 min. The resulting pellet was resuspended in ACK lysing buffer (BioWhittaker, Walkersville, MD). After incubation for 2 min on ice, cells were washed in PBS and centrifuged at 300×g for 5 min. The pellet including leukocytes was subjected to monocyte isolation by using Monocyte Isolation Kit (Miltenyi Biotec, San Diego, CA) according to the manufacturer's instructions.

Isolation and differentiation of mouse bone marrow-derived macrophages

Mouse bone marrow was collected from femur and tibia and passed through a 70 µm cell strainer in PBS, and the cell suspension was centrifuged at 300×g for 5 min. The resulting pellet was resuspended in ACK lysing buffer (BioWhittaker, Walkersville, MD). After incubation for 2 min on ice, cells were washed in PBS and centrifuged at 300×g for 5 min. The cell pellet was resuspended in RPMI1640 media containing 10% FBS, 1% penicillin-streptomycin, and M-CSF (25 ng/mL) and cultured for additional 6 days for differentiation into macrophages.

Flow cytometry and fluorescence-activated cell sorting (FACS)

Stromal vascular fraction of epididymal adipose tissue was obtained from wild-type and *Bcl2*^{AdTg} mice fed an HFD for 3 months as previously described (2).

For isolation of immune cells from the liver, liver tissues were passed through a 70 µm cell strainer in PBS, and the cell suspension was centrifuged at 30×g for 5 minutes to pellet hepatocytes. The supernatant, which was enriched in non-parenchymal cells, was centrifuged at 300×g for 10 minutes. The pellet was resuspended in 10 mL of 40% Percoll (GE Healthcare, Pittsburgh, PA) and centrifuged at 500×g for 15 min. The resulting

leukocyte pellet was resuspended in 2 mL of ACK lysing buffer (BioWhittaker, Walkersville, MD). After incubation for 2 min on ice, the cells were washed in PBS.

Single-cell suspension was stained with Zombie Yellow Kit for live/dead staining (BioLegend, San Diego, CA), and then incubated with antibodies against CD45 (eBioscience, #69-0451-82), CD11b (eBioscience, #47-0112-82), CD3 (eBioscience, # 48-0032-82), F4/80 (eBioscience, #12-4801-82), Ly-6C (eBioscience, #45-5932-82), Ly-6G (eBioscience, #17-9668-82), CX3CR1(Biolegend, #149016) and CCR2 (Biolegend, #150621) for 30 min at 4℃ in the dark.

For cell sorting, immune cells from HFD-fed *S100a8*-Cre-ires/GFP mice were stained with Zombie Yellow Kit for live/dead staining (BioLegend, San Diego, CA), and then incubated with antibodies against CD11b (eBioscience, #47-0112-82), Ly-6G (eBioscience, #17-9668-82), for 30 min at 4 °C in the dark. Then, cells were further sorted with BD FACSMelody to isolate both $CD11b^+Ly6G^-GFP^+$ and $CD11b^+Ly6G^-GFP^-$ liver macrophages.

Histological and immunohistochemical analysis

Formalin-fixed liver and adipose tissue samples were processed, and 4-μm-thick paraffin sections were subjected to various histological analyses. The 4-μm sections were further stained with hematoxylin and eosin (H&E) and Sirius Red (Sigma, St. Louis, MO). TUNEL staining was performed with an ApopTag® Peroxidase *In Situ* Apoptosis Detection Kit (Millipore, Burlington, MA). For immunohistochemistry, after heat-induced epitope retrieval, paraffin-embedded sections were incubated in 3% H_2O_2 and blocked in 3% normal serum buffer. Sections were incubated with primary antibodies overnight at 4°C. Vectastain Elite ABC Staining Kit and DAB Peroxidase Substrate Kit (Vector Laboratories, Burlingame, CA) were used to visualize the staining according to the manufacturer's instructions. Primary antibody specific to F4/80 was obtained from Cell Signaling Technology (Danvers, MA). For immunofluorescence staining, after an overnight incubation with the primary antibody at 4°C, samples were incubated with the secondary antibody for 1 h at room temperature. Primary antibodies against Perilipin-1 and S100A8 were obtained from Cell Signaling Technology (Danvers, MA). Primary antibody against

IBA1 was purchased from MilliporeSigma (St. Louis, MO). Goat anti-rabbit IgGs - Alexa Fluor 488 (#4412) and 555 (#4413) were obtained from Cell Signaling Technology (Danvers, MA). Nuclear staining was performed by incubation with 4’,6-diamidino-2-phenylindole for 5 min. Images were acquired using either a BX41 microscope for bright field images (Olympus, Bethlehem, PA) or a LSM710 confocal microscope (Zeiss, Thornwood, NY) for fluorescent images. Positive cells and positive areas in 10 randomly selected high-power fields were analyzed.

For immunofluorescence staining of human samples, formalin-fixed paraffin-embedded healthy and NASH patient liver samples were obtained from the Department of Surgery at Johns Hopkins Hospital (supported by the NIAAA, R24AA025017). After heat-induced epitope retrieval in antigen retrieval buffer (10 mM Tris-HCl and 1 mM EDTA with 10% glycerol [pH 9]) sections were stained with primary antibodies and visualized by LSM 900 Confocal Microscope (Zeiss, Thornwood, NY). Multiplex immunofluorescence staining with more than three markers was performed as previously described (3). Acquired images were processed and analyzed using ImageJ FIJI (4).

Primary antibodies used in the staining:

Antibody	Vendor	Cat. No.	Dilution
F4/80	Cell Signaling Technology	70076	1:200
Perilipin-1	Cell Signaling Technology	9349	1:200
IBA1	Sigma	MABN92	1:200
HepPar1	Novus Biologicals	NBP2-45272	1:200
CD36	Novus Biologicals	NB400-144	1:200
S100A8	Cell Signaling Technology	33254	1:200

Immunoblot analysis

Liver and adipose tissues were homogenized in RIPA buffer containing a cocktail of protease inhibitors (Santa Cruz Biotechnology, Dallas, TX) according to the manufacturer's instructions. Protein extracts were loaded onto 4-12% Bis-Tris protein gels (Bio-Rad, Hercules, CA) and transferred onto nitrocellulose membranes (Thermo Fisher,

Waltham, MA). Protein bands were visualized with Pierce ECL Western Blotting Substrate (Thermo Fisher, Waltham, MA). The antibodies against BCL2 (#4223) and PPARγ (#2435) were purchased from Cell Signaling Technology (Danvers, MA). The antibody against β-actin (#ab8227) was purchased from Abcam (Cambridge, MA). Anti-rabbit IgG linked with horseradish peroxidase (#7074) was obtained from Cell Signaling Technology (Danvers, MA).

Total RNA isolation and RT-qPCR

Total RNA was purified from liver tissues or cell cultures using TRIzol reagents (Thermo Fisher, Waltham, MA) according to the manufacturer's instructions. One microgram of RNA was reverse-transcribed into cDNA using a High-Capacity cDNA Reverse Transcription Kit (Thermo Fisher). The expression levels of mRNA were measured by RT-qPCR with an ABI7500 RT-PCR system (Applied Biosystems, Foster City, CA). *Gapdh* or *Apob* was used as the invariant control. The $2^{-\Delta\Delta Ct}$ method was used to calculate the level of mRNA. The primer sequences used for PCR reactions are listed in Supplementary Table S1.

Conjugation of fatty acids with bovine serum albumin (BSA)

Fatty acid–free BSA was purchased from GenDepot (Baker, TX). Palmitic acid, oleic acid, and elaidic acid were obtained from Sigma-Aldrich (St. Louis, MO). Fatty acid stock solutions (75 mM in ethanol) and BSA solution (5% [w/v] in culture medium) were preheated to 60 °C. The fatty acid solution was then added dropwise to the 5% BSA solution to yield a final concentration of 5 mM fatty acid and 5% BSA (0.8 mM), corresponding to a 6:1 molar ratio of fatty acid to BSA. The mixture was sonicated until clear to ensure complete solubilization.

Measurement of free fatty acids

Free fatty acids in the culture supernatant were quantified as previously reported (5). Briefly, differentiated 3T3-L1 cells were treated with 2 ng/mL FasL or vehicle for 12 h, washed with PBS, and subsequently cultured for an additional 1 h. The culture supernatant was then analyzed for free fatty acid content using the LabAssay NEFA Assay Kit (WAKO, Neuss, Germany) according to the manufacturer's instructions.

Measurement of free fatty acid uptake

Free fatty acid uptake was assessed using a Fatty Acid Uptake Assay kit (Dojindo, Kumamoto, Japan) following the manufacturer's protocol. AML12 cells were treated with recombinant mouse CCN3 protein (R&D Systems, Minneapolis, MN) or vehicle for 24 h. After treatment, cells were incubated with the fatty acid uptake probe for 15 min. Fluorescence was then measured using a microplate reader (Tecan, Mannedorf, Switzerland) at 485/535 nm (excitation/emission) wavelengths.

Electroporation of siRNAs

Bone marrow-derived macrophages were electroporated with siRNAs targeting the mouse *S100a8* gene (no. 4392420, assay no. s201956, Thermo Fisher), mouse *Ccn3* gene (no. 4390771, assay no. s201735, Thermo Fisher), or the green fluorescence protein (Bioneer, Daejeon, Korea) as a negative control. Electroporation was performed using ECM830 (BTX, Shelton, CT).

Measurement of cell viability

Cell viability was measured using a CCK-8 assay kit (Dojindo, Kumamoto, Japan) according to the manufacturer's instructions. Briefly, bone marrow-derived macrophages or AML12 cells were treated with PA for 24 h. After the treatment period, the cells were further incubated with CCK-8 reagent for 90 min, and the absorbance was measured at 450 nm using a microplate reader (Tecan, Mannedorf, Switzerland).

Isolation of extracellular vesicles

Extracellular vesicles (EVs) were isolated as previously described (6). Briefly, after centrifugation of culture medium at 3,000×g for 15 min at 4°C, the supernatant fraction including EVs was incubated with an appropriate volume of ExoQuick-TC exosome precipitation solution (System Biosciences, Palo Alto, CA) and subjected to EV isolation procedure according to the manufacturer's protocol. The concentration of EVs was measured by using the Pierce BCA protein assay kit (Thermo Fisher, Waltham, MA) and EXOCET Exosome Quantitation Kit (System Biosciences, Palo Alto, CA).

References

1. Zebisch K, et al. Protocol for effective differentiation of 3T3-L1 cells to adipocytes. *Anal Biochem.* 2012;425(1):88-90.
2. Bowles AC, et al. Isolation and Flow Cytometric Analysis of the Stromal Vascular Fraction Isolated from Mouse Adipose Tissue. *Methods Mol Biol.* 2018;1773:1-9.
3. Ma J, et al. Distinct histopathological phenotypes of severe alcoholic hepatitis suggest different mechanisms driving liver injury and failure. *J Clin Invest.* 2022;132(14).
4. Schindelin J, et al. Fiji: an open-source platform for biological-image analysis. *Nat Methods.* 2012;9(7):676-682.
5. Rapold RA, et al. Fas activates lipolysis in a Ca2+-CaMKII-dependent manner in 3T3-L1 adipocytes. *J Lipid Res.* 2013;54(1):63-70.
6. Hwang S, et al. Interleukin-22 Ameliorates Neutrophil-Driven Nonalcoholic Steatohepatitis Through Multiple Targets. *Hepatology.* 2020;72(2):412-429.

Supplementary Table S1. Primer sequences for RT-qPCR

Species	Gene	Forward (5'-3')	Reverse (5'-3')
Mouse	*Acc1*	TGGACAGACTGATCGCAGAGAAAG	TGGAGAGCCCCACACACA
Mouse	*Acta2*	TCCTGACGCTGAAGTATCCGATA	GGTGCCAGATCTTTTCCATGTC
Mouse	*Adgre1*	CTTTGGCTATGGGCTTCCAGTC	GCAAGGAGGACAGAGTTTATCGTG
Mouse	*Anxa1*	ATGTATCCTCGGATGTTGCTGC	TGAGCATTGGTCCTCTTGGTA
Mouse	*Apob*	CGTGGGCTCCAGCATTCTA	TCACCAGTCATTTCTGCCTTTG
Mouse	*Apoe*	CTCCCAAGTCACACAAGAACTG	CCAGCTCCTTTTTGTAAGCCTTT
Mouse	*Bak*	CAACCCCGAGATGGACAACTT	CGTAGCGCCGGTTAATATCAT
Mouse	*Bax*	TGAAGACAGGGGCCTTTTTG	AATTCGCCGGAGACACTCG
Mouse	*Bcl2*	GTCGCTACCGTCGTGACTTC	CAGACATGCACCTACCCAGC
Mouse	*Bclxl*	GACAAGGAGATGCAGGTATTGG	TCCCGTAGAGATCCACAAAAGT
Mouse	*C1qa*	TTCGGCAGAACCCAATGACG	TGGTATGGACTCTCCTGGTTG
Mouse	*C1qb*	CGTCGGCCCTAAGGGTACT	GGGGCTGTTGATGGTCCTC
Mouse	*C1qc*	GGACGGGCATGATGGACTC	TTCTGTTTGTATCGGCCCTCC
Mouse	*Ccl2*	TCTGGACCCATTCCTTCTTGG	TCAGCCAGATGCAGTTAACGC
Mouse	*Cd36*	CCTGCAAATGTCAGAGGAAA	GCGACATGATTAATGGCACA
Mouse	*Cd68*	TGTCTGATCTTGCTAGGACCG	GAGAGTAACGGCCTTTTTGTGA
Mouse	*Ccn3*	GGCCTCTCAGCTCATGGTTT	AGGTGGCTTGGTCTTCAGTG
Mouse	*Ccn5*	TGTGTGACCAGGCAGTGATGCA	CAGGCTGTGCTCCAGTTTGGAC
Mouse	*Col1a1*	TAGGCCATTGTGTATGCAGC	ACATGTTCAGCTTTGTGGACC
Mouse	*Col1a2*	GGTGAGCCTGGTCAAACGG	ACTGTGTCCTTTCACGCCTTT
Mouse	*Col3a1*	TAGGACTGACCAAGGTGGCT	GGAACCTGGTTTCTTCTCACC
Mouse	*Col4a1*	CACATTTTCCACAGCCAGAG	GTCTGGCTTCTGCTGCTCTT
Mouse	*Cxcl1*	ACTGCACCCAAACCGAAGTC	TGGGGACACCTTTTAGCATCTT
Mouse	*Cxcl2*	TCCAGGTCAGTTAGCCTTGC	CGGTCAAAAAGTTTGCCTTG
Mouse	*Fabp4*	GGGGATTTGGTCACCATCCG	TTGTGGTCGACTTTCCATCCC

Mouse	*Fasn*	GCTGCGGAAACTTCAGGAAAT	AGAGACGTGTCACTCCTGGACTT
Mouse	*Gapdh*	AGCAGCCGCATCTTCTTGTGCAGTG	GGCCTTGACTGTGCCGTTGAATTT
Mouse	*Gpat1*	ACAGTTGGCACAATAGACGTTT	CCTTCCATTTCAGTGTTGCAGA
Mouse	*Icam1*	CAATTTCTCATGCCGCACAG	AGCTGGAAGATCGAAAGTCCG
Mouse	*Ifnb*	CCCTATGGAGATGACGGAGA	CCCAGTGCTGGAGAAATTGT
Mouse	*Ifng*	TAGCCAAGACTGTGATTGCGG	AGACATCTCCTCCCATCAGCAG
Mouse	*Il1b*	TCGCTCAGGGTCACAAGAAA	CATCAGAGGCAAGGAGGAAAAC
Mouse	*Il6*	ACAAGTCGGAGGCTTAATTACACAT	TTGCCATTGCACAACTCTTTTC
Mouse	*S100a8*	CAAGGAAATCACCATGCCCTCTA	ACCATCGCAAGGAACTCCTCGA
Mouse	*S100a9*	TGGGCTTACACTGCTCTTACC	GGTTATGCTGCGCTCCATCT
Mouse	*Scd1*	CCGGAGACCCCTTAGATCGA	TAGCCTGTAAAAGATTTCTGCAAACC
Mouse	*Srebf1*	GGAGCCATGGATTGCACATT	GGCCCGGGAAGTCACTGT
Mouse	*Tgfb1*	CAACCCAGGTCCTTCCTAAA	GGAGAGCCCTGGATACCAAC
Mouse	*Tnfa*	AGGCTGCCCCGACTACGT	GACTTTCTCCTGGTATGAGATAGCAAA
Mouse	*Trem2*	CTGGAACCGTCACCATCACTC	CGAAACTCGATGACTCCTCGG

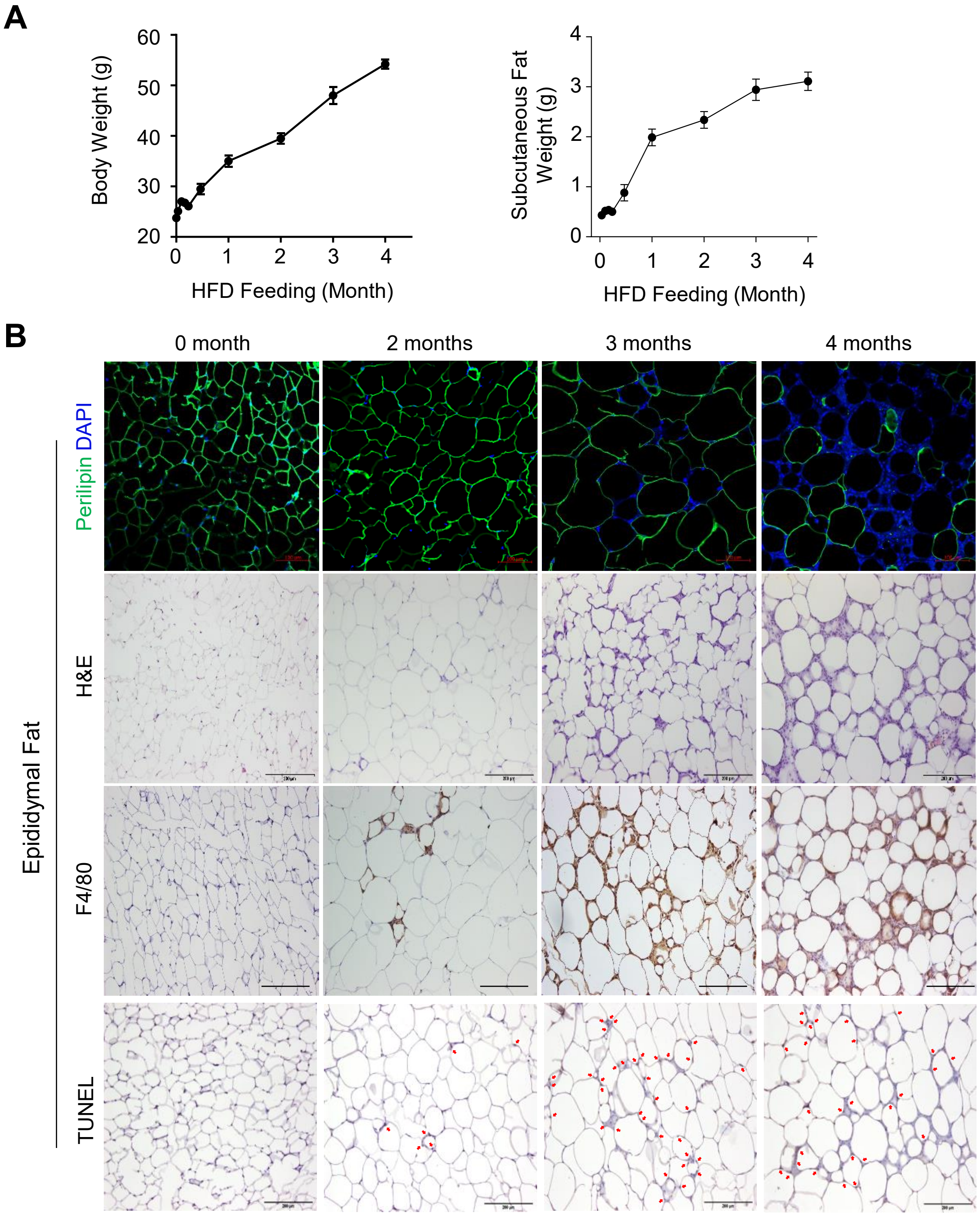

Supplementary Figure S1. Temporal changes in the adipose tissues of mice fed an HFD. Male C57BL6/J mice were fed an HFD for the specified durations. Epididymal and subcutaneous fat tissues were collected and processed for further analyses. (A) Temporal changes in body weight and subcutaneous fat weight. Values represent mean ± SEM. (B) Paraffin-embedded epididymal fat tissues were subjected to perilipin staining, H&E staining, F4/80 staining, and TUNEL staining. Scale bars indicate 100 μm (perilipin images) and 200 μm (H&E, F4/80, TUNEL images). Red arrows indicate TUNEL-positive cells.

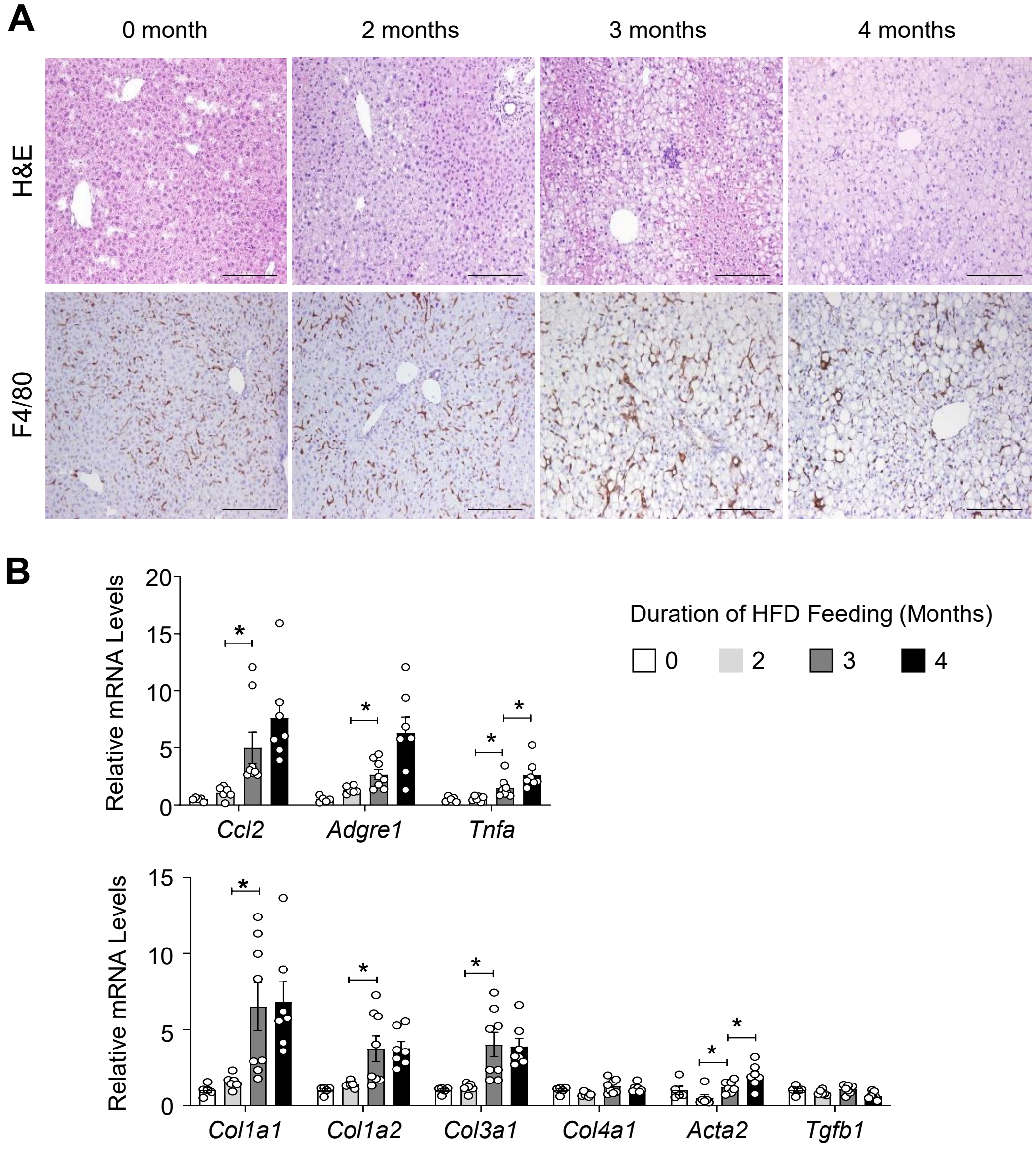

Supplementary Figure S2. Temporal changes in the livers of mice fed an HFD. Male C57BL6/J mice were fed an HFD for the specified durations. The livers of mice were obtained and subjected to further analysis. (A) Paraffin-embedded liver tissues were subjected to H&E staining and F4/80 staining. Scale bars indicate 200 µm. (B) RT-qPCR analysis of genes involved in inflammation (top) and fibrosis (bottom). *Apob* was used as a reference gene. Values represent mean ± SEM. Statistical evaluation was performed by Student's t-test (*p<0.05).

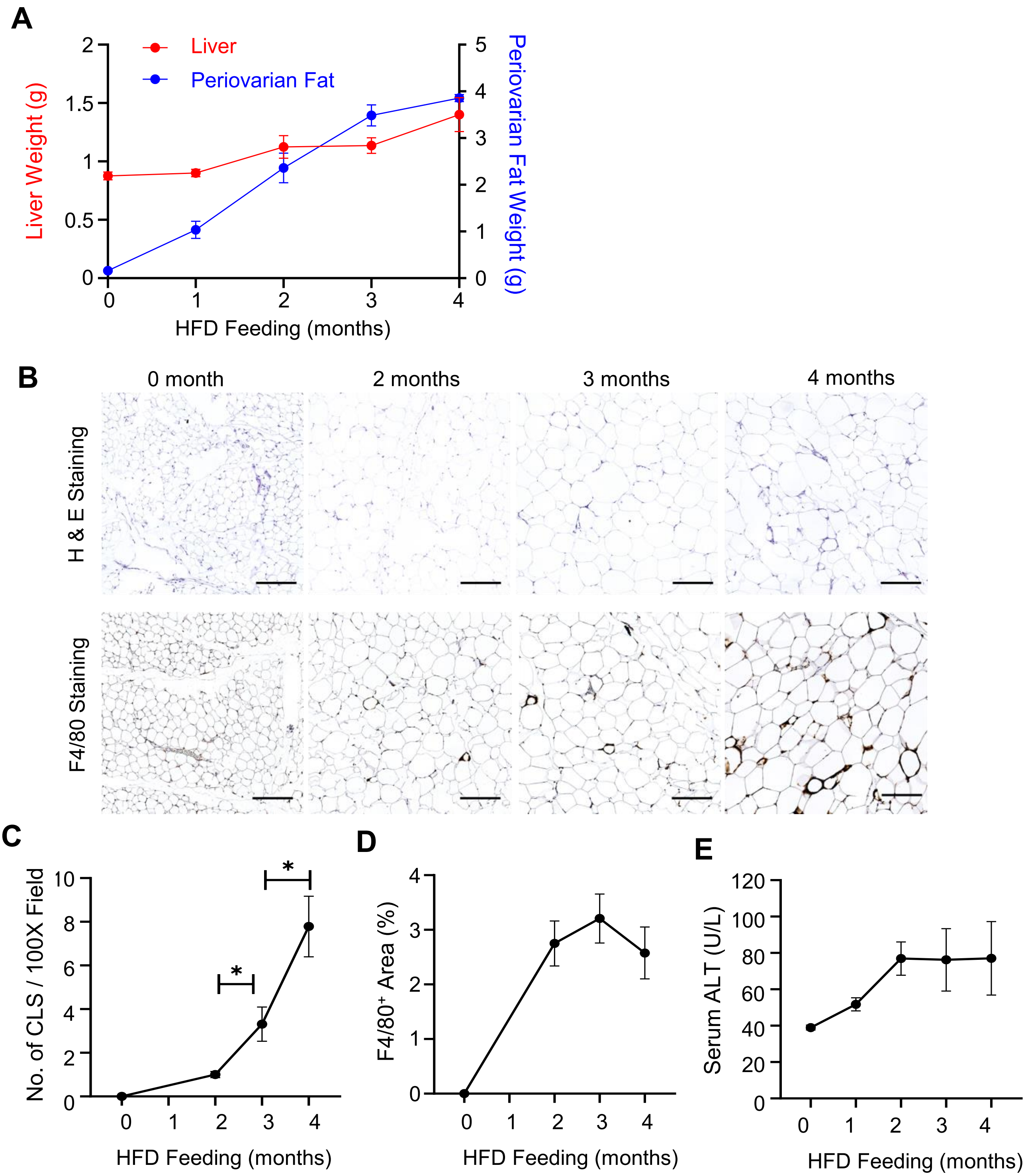

Supplementary Figure S3. Characterization of female mice following 4-month HFD feeding. Female C57BL/6J mice (n=6–7/group) were fed an HFD for up to 4 months. (A) Time-course analysis of periovarian fat weight and liver weight during HFD feeding. (B) Paraffin-embedded adipose tissue sections were analyzed by hematoxylin and eosin (H&E) staining and immunohistochemical staining for F4/80. Scale bars indicate 200 µm. (C–D) Quantification of crown-like structures (panel C) and F4/80-positive area (panel D) is shown. (E) Serum ALT levels. Data are presented as mean ± SEM. Statistical significance was determined using one-way ANOVA followed by Tukey's post hoc test for multiple comparisons (*$p < 0.05$).

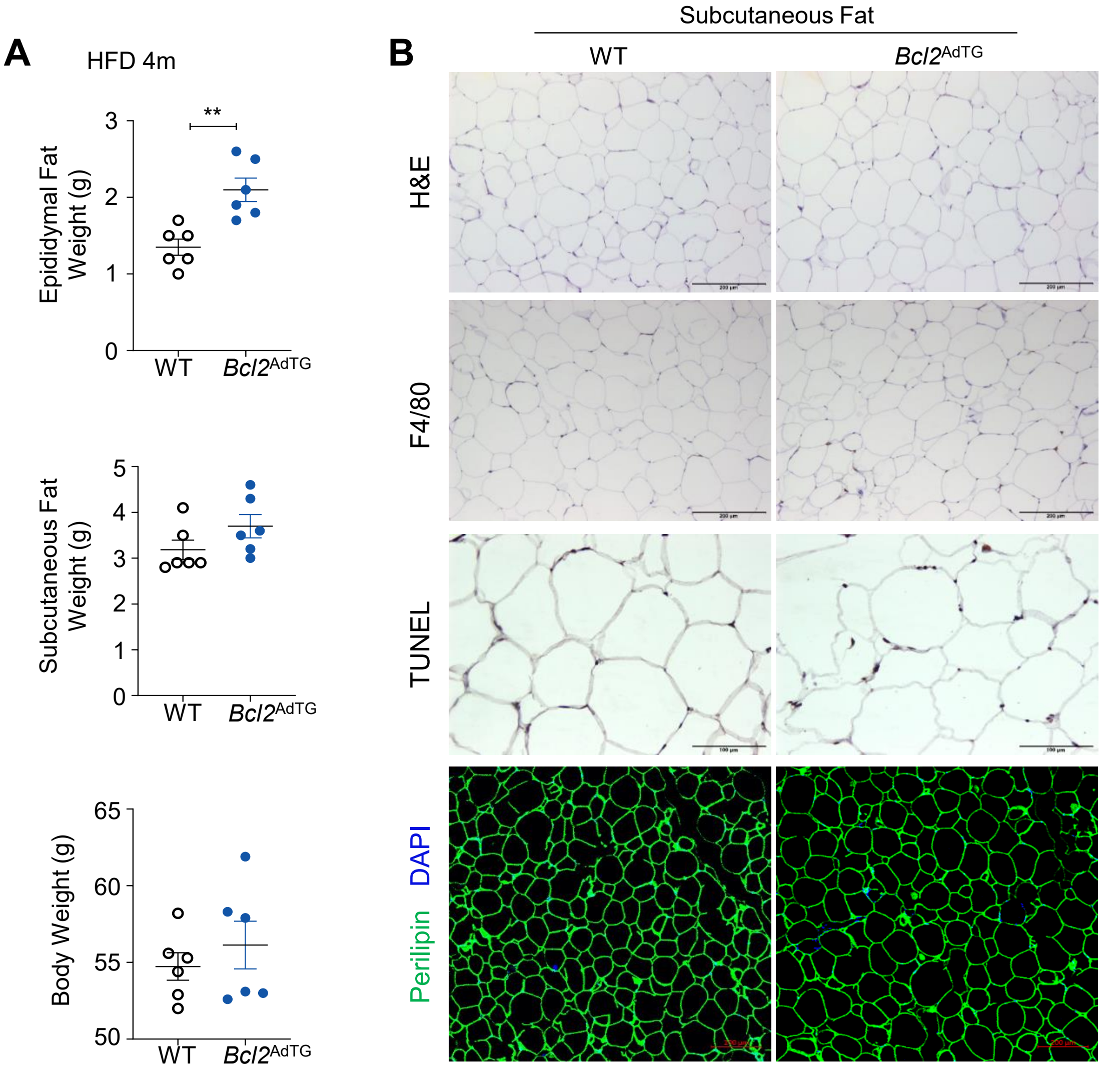

Supplementary Figure S4. Analysis of adipose tissues in HFD-fed *Bcl2*$^{\mathbf{AdTG}}$ mice. Male *Bcl2*^{AdTG} mice and WT littermates were fed an HFD for 4 months, and adipose tissues were collected for further analysis. (A) Epididymal fat weight, subcutaneous fat weight, and body weight. Values represent mean ± SEM. Statistical evaluation was performed by Student's t-test (**p<0.01). (B) Paraffin-embedded subcutaneous fat tissues were subjected to H&E, F4/80, TUNEL, and perilipin staining. Scale bars indicate 200 μm (H&E and F4/80) and 100 μm (TUNEL and perilipin).

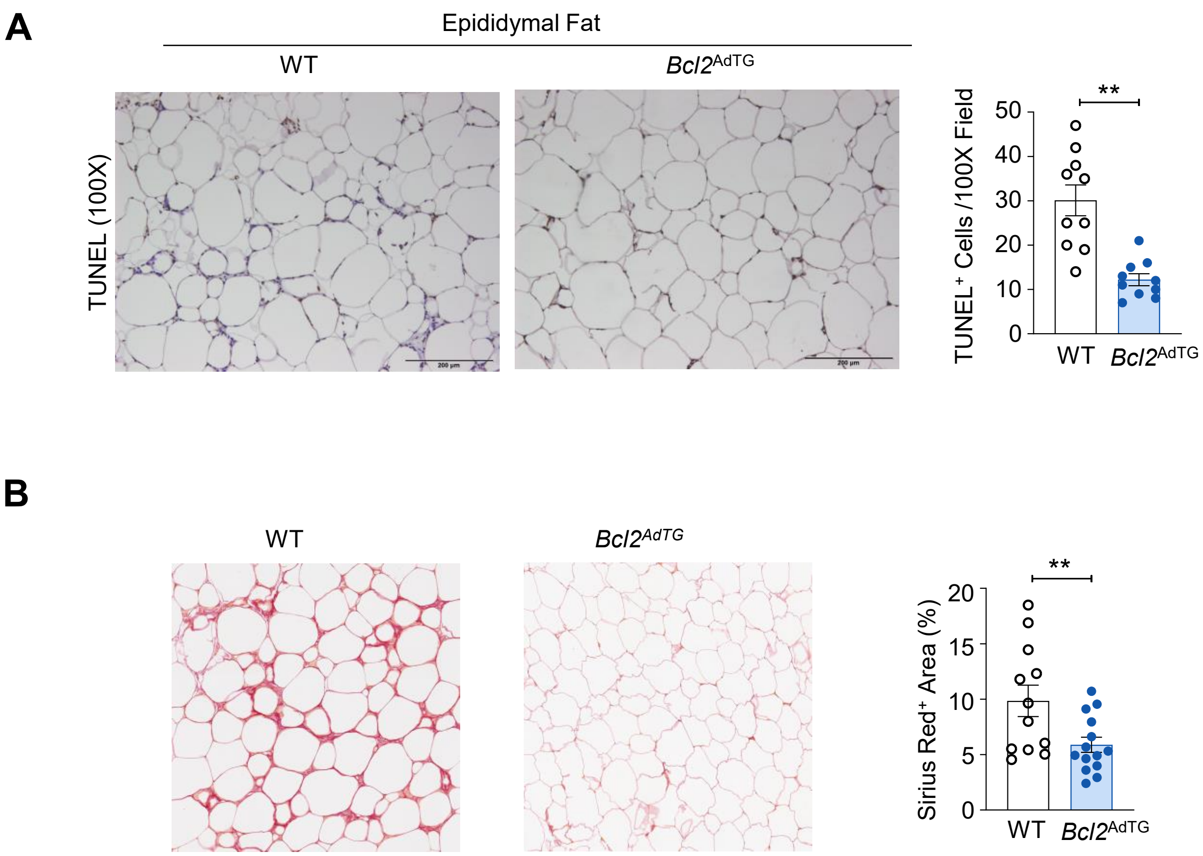

Supplementary Figure S5. Attenuation of adipose tissue fibrosis by adipocyte-specific overexpression of *Bcl2*. *Bcl2*^{AdTG} mice and wild-type littermate controls were fed an HFD for 4 months. (A) Paraffin-embedded epididymal adipose tissue sections were subjected to TUNEL staining. Scale bars indicate 200 μm. (B) Paraffin-embedded epididymal fat tissues were subjected to Sirius Red staining. Data are presented as mean ± SEM. Statistical significance was determined using Student's t-test (**p*<0.05, ***p*<0.01).

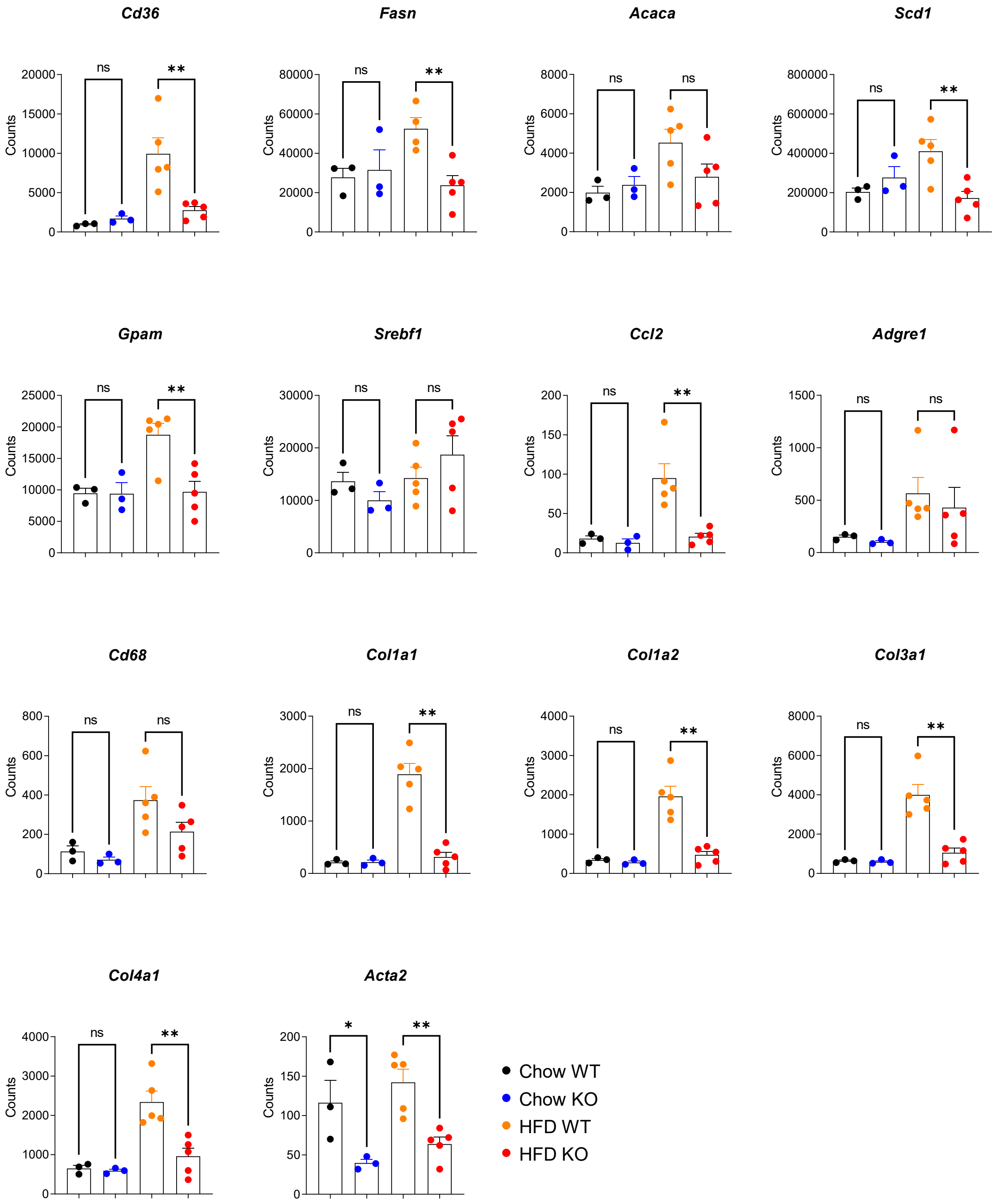

Supplementary Figure S6. *Bcl2* overexpression in adipocytes reverses HFD-induced lipogenic, inflammatory, and fibrotic gene expression in the liver. RNA sequencing was performed on liver tissues from chow- and HFD-fed wild-type (WT) and $Bcl2^{AdTG}$ mice. Gene count data are shown for representative lipogenic genes (e.g., *Fasn*, *Scd1*), fatty acid transporter *Cd36*, inflammatory genes (e.g., *Ccl2*), and fibrotic markers (e.g., *Col1a1*). Data are presented as mean ± SEM. Statistical significance was determined using Student's t-test (**p<0.01).

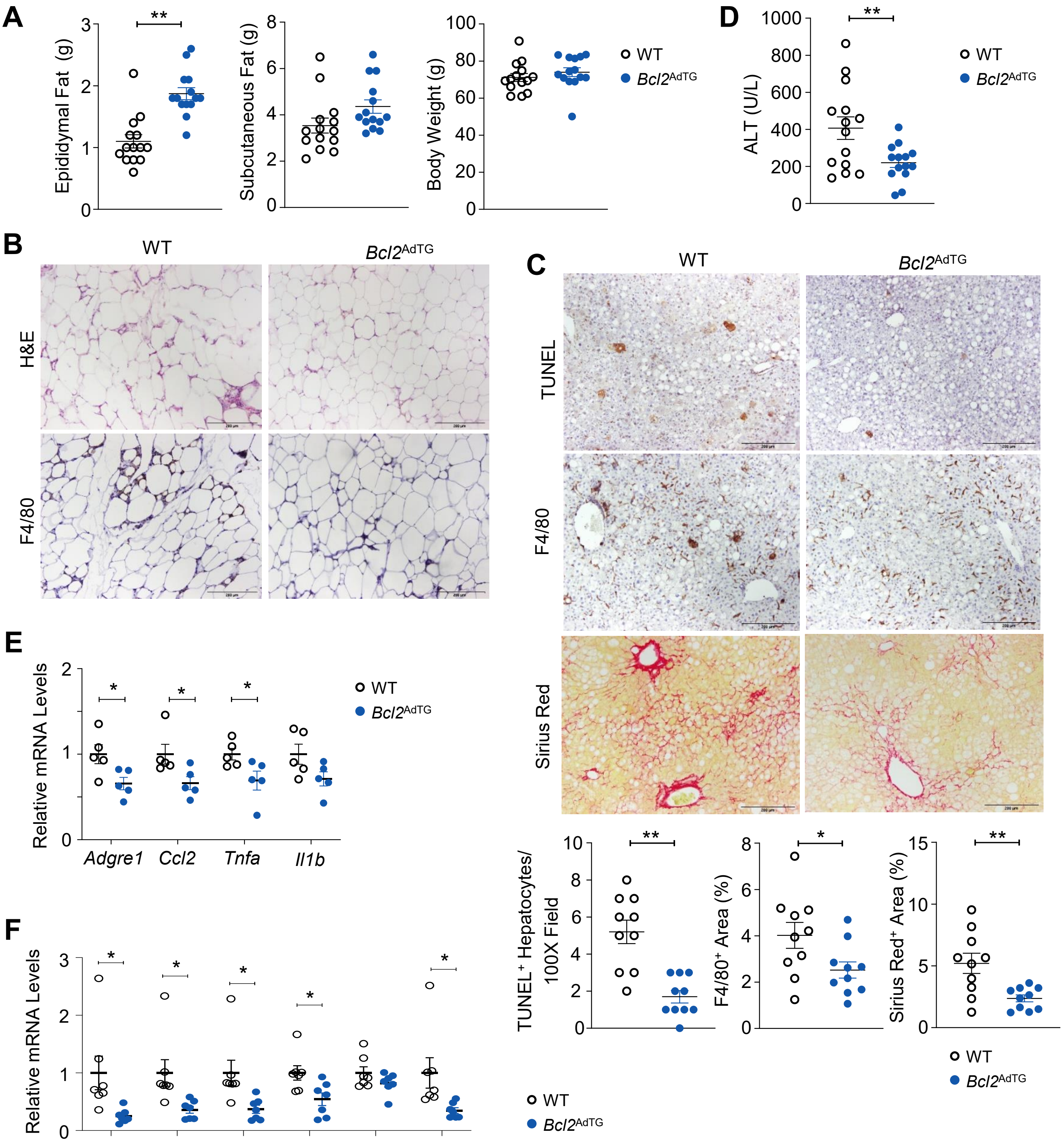

Supplementary Figure S7. Overexpression of the *Bcl2* gene in adipocytes attenuates MASLD in mice fed an HFD for 1 year. *Bcl2*^{AdTG} and WT littermates were fed an HFD for 1 year. (A) Weights of epididymal fat, subcutaneous fat, and total body were measured. (B) H&E staining (top) and F4/80 staining (bottom) of epididymal fat. (C) TUNEL staining (left), F4/80 staining (center), and Sirius Red staining (right) of the liver. Scale bars indicate 200 µm (D) Serum ALT levels. (E-F) RT-qPCR analysis of genes in the liver. Values represent mean ± SEM. Statistical evaluation was performed by Student's t-test (*$p<0.05$; **$p<0.01$).

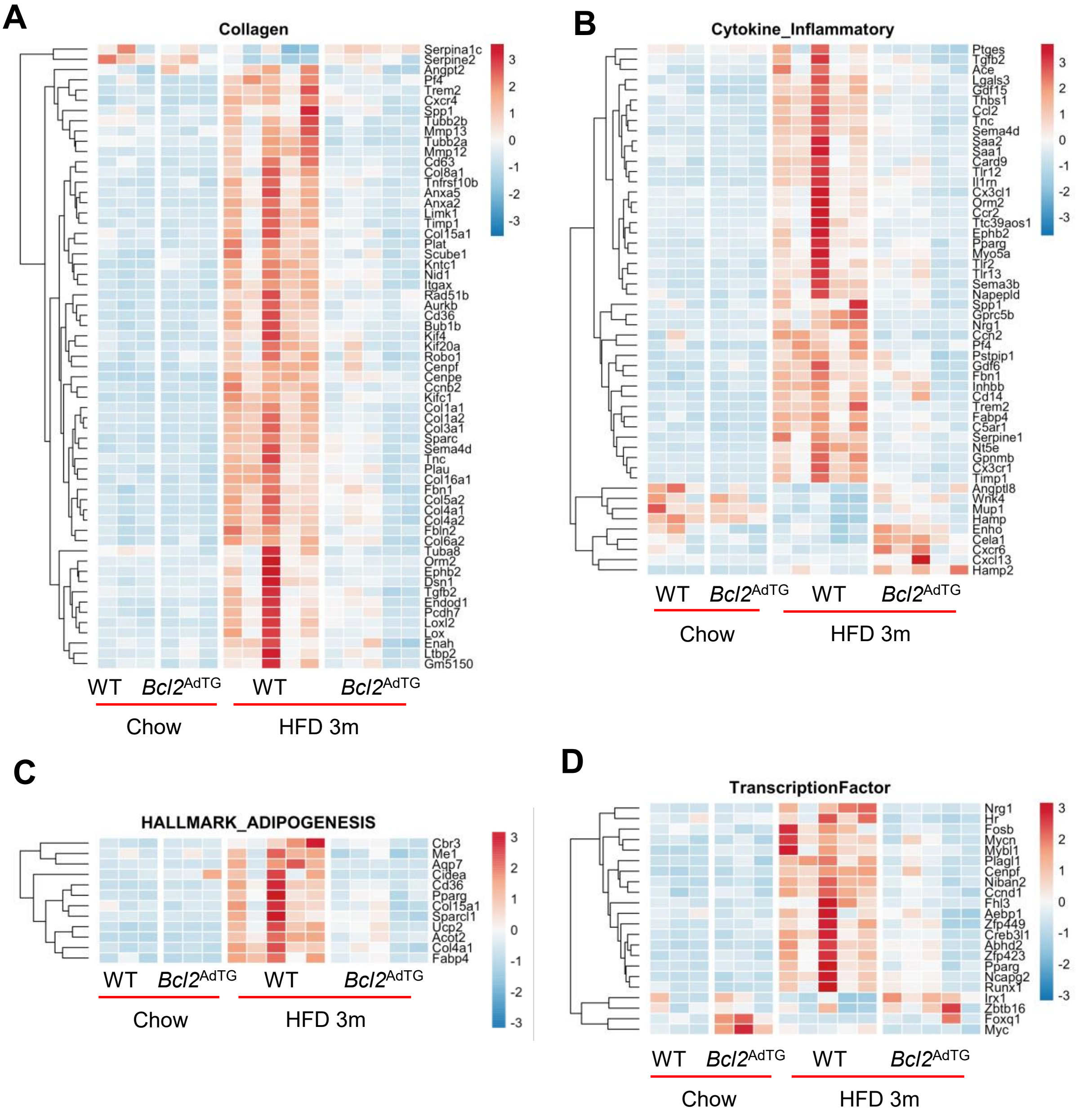

Supplementary Figure S8. RNA-seq analysis shows *Bcl2* overexpression in adipocytes reverses gene changes in the livers of HFD-fed WT mice. (A-D) Heatmaps of collagen-related genes, cytokine and inflammatory genes, adipogenesis-related genes, and transcription factors in the liver of chow diet- and HFD-fed *Bcl2*^{AdTG} and WT mice.

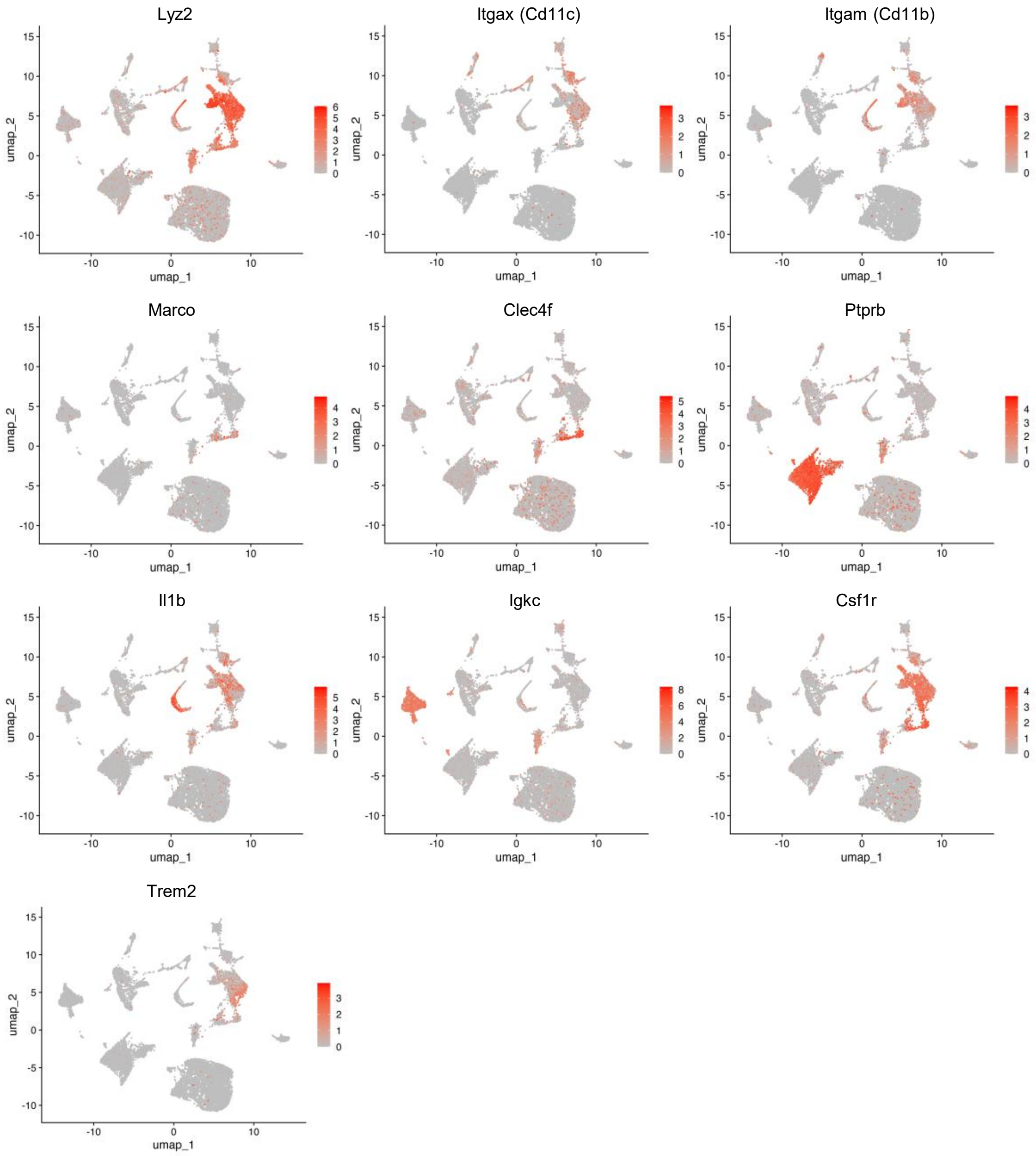

Supplementary Figure S9. Single-cell RNA sequencing reveals signature genes in the population of S100A8⁺ macrophages in the liver of HFD-fed *Bcl2*^{AdTG} and WT mice. Feature plots showing the expression of macrophage signature genes across all cells.

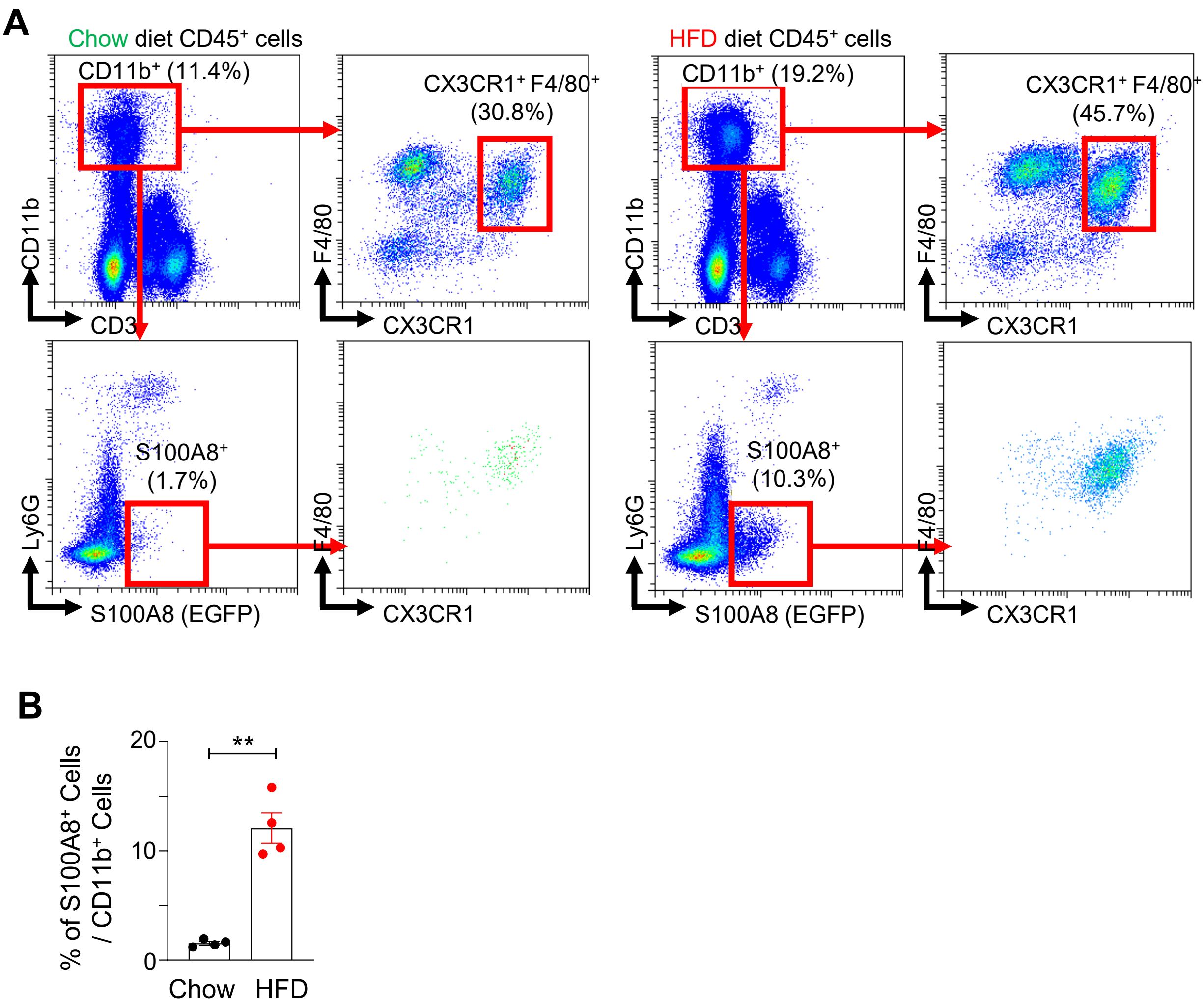

Supplementary Figure S10. HFD feeding increases S100A8^{+} macrophages in the liver of HFD-fed mice. *S100a8*-Cre-ires/GFP mice were fed a chow or HFD for 4 months. (A) Flow cytometry of liver mononuclear cells revealed the enrichment of S100A8 (EGFP)$^{+}$ cells in the liver of HFD-fed mice, which were identified as CX3CR1^{+}F4/80^{+} macrophages. (B) Statistical analysis of the percentage of S100A8^{+} (EGFP^{+}) cells within CD11b^{+} cells. Values represent mean ± SEM. Statistical evaluation was performed by Student's t-test (**$p < 0.01$).

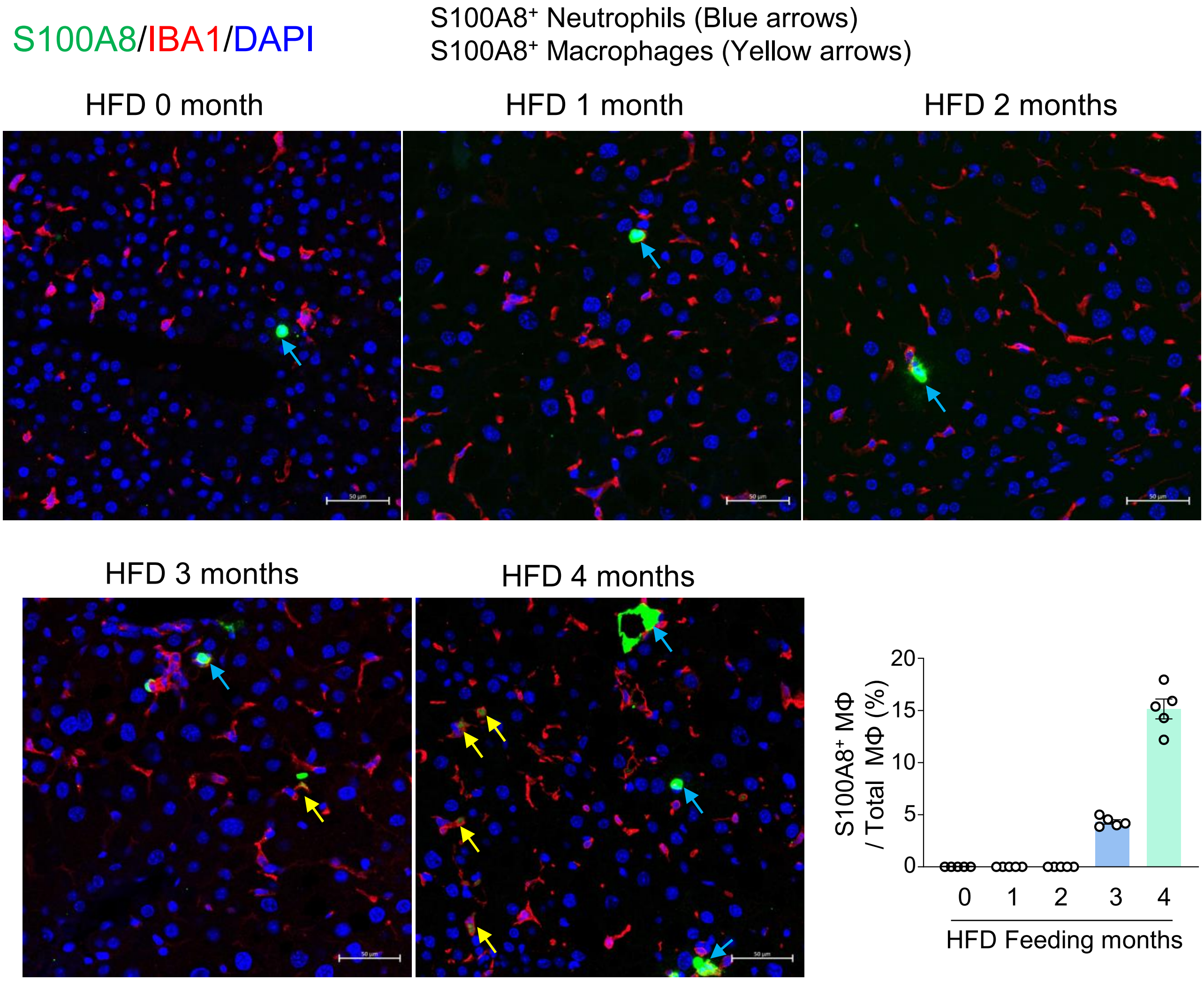

Supplementary Figure S11. HFD feeding increases hepatic S100A8$^+$ macrophages in mice. Male C57BL/6J mice were fed an HFD for up to 4 months (n= 4-5/group). Paraffin-embedded liver sections were analyzed by immunohistochemistry for IBA1 and S100A8. Yellow arrows indicate S100A8$^+$ macrophages, and blue arrows indicate S100A8$^+$ neutrophils. The percentage of S100A8$^+$ macrophages among total macrophages was quantified per field. Scale bars indicate 50 μm. Values represent mean ± SEM. MΦ, macrophages.

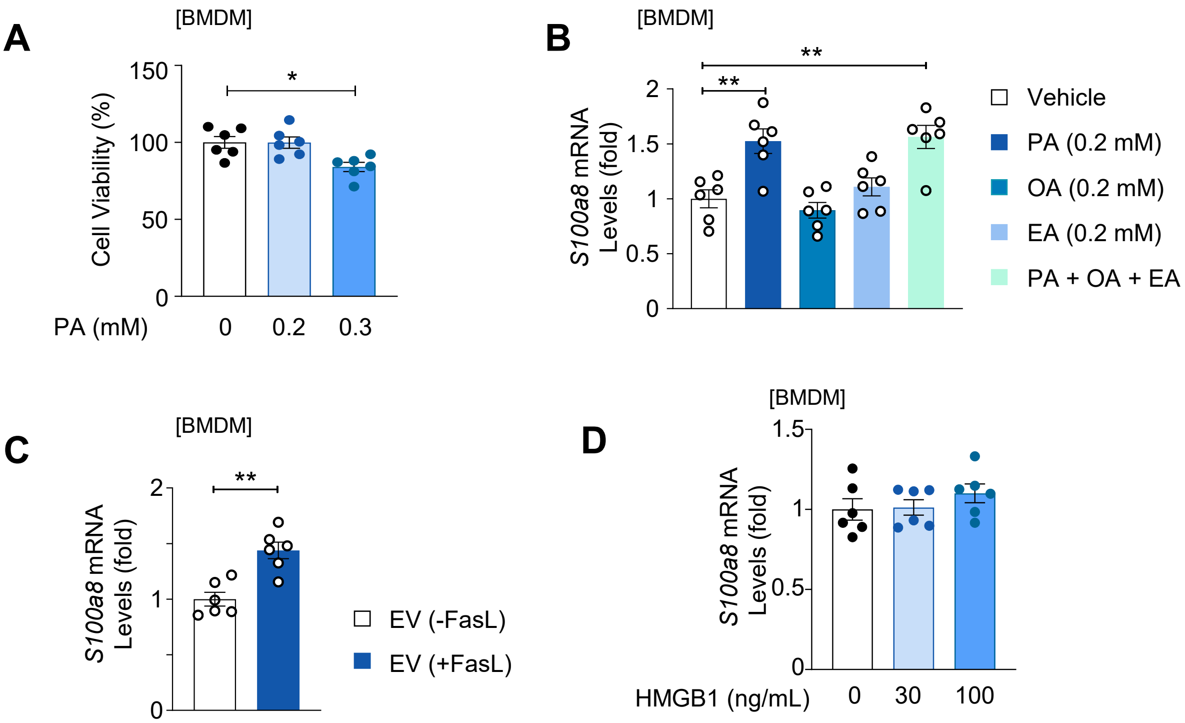

Supplementary Figure S12. Effect of free fatty acids, extracellular vesicles, and HMGB1 on the cell viability and *S100A8* mRNA expression in macrophages. (A) Bone marrow-derived macrophages (BMDMs) were treated with PA for 24 h. Cell viability was assessed using the CCK-8 assay (n=6; data combined from two independent experiments). (B) BMDMs were treated with palmitic acid (PA), oleic acid (OA), and/or elaidic acid (EA) for 24 h. *S100a8* mRNA levels were analyzed by RT-qPCR (n=6; data represent the combined results of two independent experiments). (C) Bone marrow-derived macrophages (BMDMs) were treated extracellular vesicles (EVs) collected from 3T3-L1 cells treated with FasL (2 ng/mL) or vehicle for 24 h. *S100a8* mRNA levels were analyzed by RT-qPCR (n=6; data represent the combined results of two independent experiments). (D) BMDMs were treated with HMGB1 (30 or 100 µM) for 24 h. *S100a8* mRNA levels were measured by RT-qPCR (n=6; data combined from two independent experiments). Values represent mean ± SEM. Statistical evaluation was performed using Student's t-test (panel C) or one-way ANOVA with Tukey's post hoc test for multiple comparisons (panel A, B and D). (*p < 0.05, **p < 0.01).

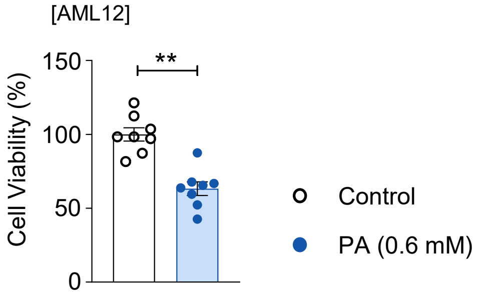

Supplementary Figure S13. Effect of palmitic acid on the cell viability of AML12 hepatocytes. AML12 cells were treated with PA (0.6 mM) for 24 h. Cell viability was assessed using the CCK-8 assay (n=8; data combined from two independent experiments). Values represent mean ± SEM. Statistical evaluation was performed using Student's t-test (**p < 0.01).

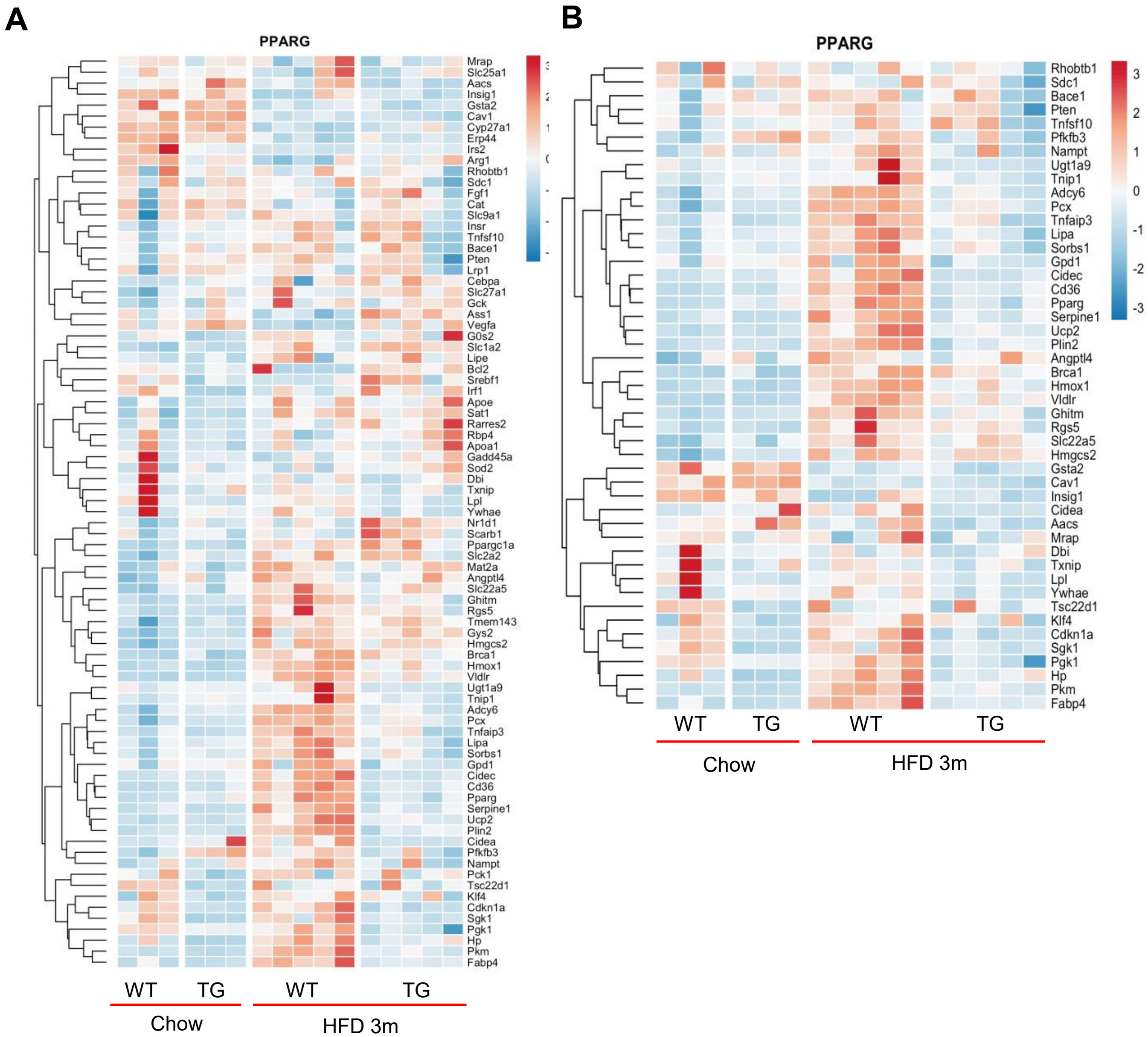

Supplementary Figure S14. RNA-seq analysis shows reduced expression of selected hepatic PPARγ target genes in the liver of HFD-fed *Bcl2^AdTG^* mice. Heatmaps of hepatic PPARγ target genes in the liver of chow diet- and HFD-fed *Bcl2*^{AdTG} and WT mice. (A) The expression of the entire PPARγ target genes. (B) The selected PPARγ target genes that are downregulated by *Bcl2* overexpression.

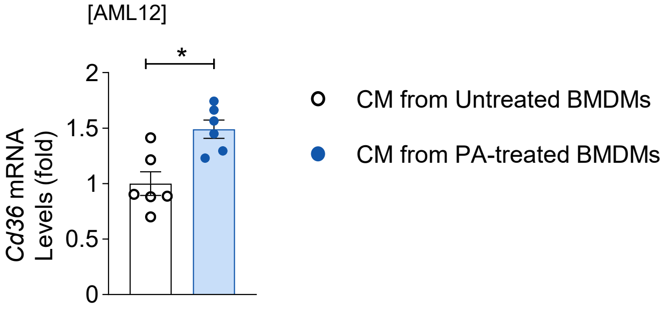

Supplementary Figure S15. Removal of palmitic acid from the conditioned media does not affect the ability of the conditioned media to induce CD36 in hepatocytes. Bone marrow-derived macrophages (BMDMs) were treated with palmitic acid (PA, 0.2 mM) or vehicle for 6 h, after which the media were removed. The PA- or vehicle-treated cells were then further cultured in fresh media for an additional 18 h to obtain the conditioned media (CM). Afterwards, AML12 cells were treated with the CM collected from BMDMs treated with PA (0.2 mM) or vehicle for 24 h. *Cd36* mRNA levels were analyzed by RT-qPCR (n=6; data represent the combined results of two independent experiments). Statistical evaluation was performed by Student's t-test (*p<0.05, **p<0.01).

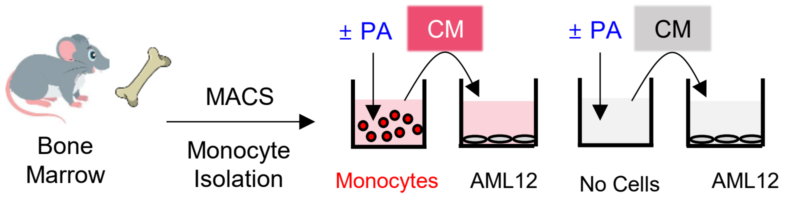

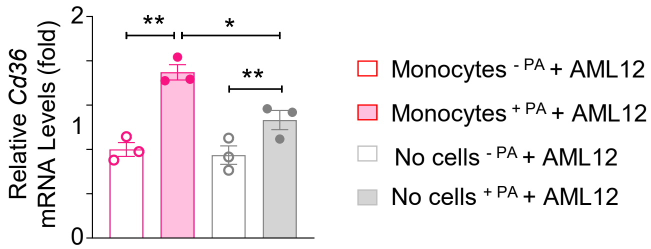

Supplementary Figure S16. Conditioned media from PA-treated monocytes promote *Cd36* induction in AML12 cells. Monocytes isolated from mouse bone marrow by magnetic-activated cell sorting (MACS) were treated with palmitic acid (PA, 0.2 mM) or vehicle for 24 h to prepare conditioned media (CM). A control CM was prepared by adding PA or vehicle to the culture media without monocytes. AML12 cells were incubated with either the CM obtained from monocyte cultures or with the control CM for 24 h. RNA was extracted from AML12 cells and subjected to the RT-qPCR analysis of *Cd36*. *Gapdh* was used as a reference gene. Values represent mean ± SEM. Statistical evaluation was performed using one-way ANOVA with Tukey's post hoc test for multiple comparisons. (**p<0.01).

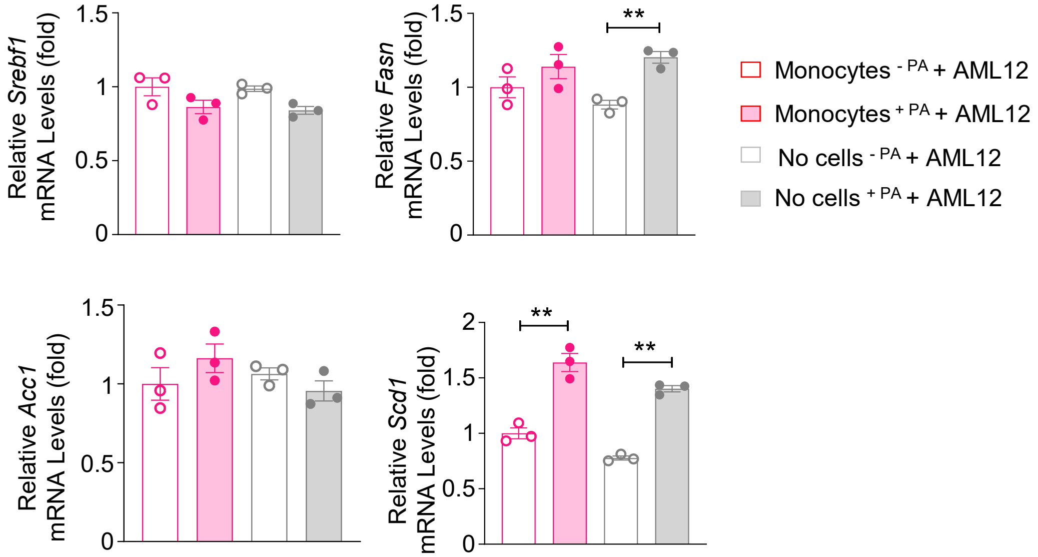

Supplementary Figure S17. Expression of lipogenic genes in AML12 cells exposed to PA-treated monocytes. Monocytes isolated from mouse bone marrow by magnetic-activated cell sorting (MACS) were treated with PA (0.2 mM) or vehicle for 24 h to prepare conditioned media (CM). A control CM was prepared by adding PA or vehicle to the culture media without monocytes. AML12 cells were incubated with either the CM obtained from monocyte cultures or with the control CM for 24 h. RNA was extracted from AML12 cells and subjected to the RT-qPCR analysis of lipogenic genes. *Gapdh* was used as a reference gene. Values represent mean ± SEM. Statistical evaluation was performed using one-way ANOVA with Tukey's post hoc test for multiple comparisons. (**p<0.01).

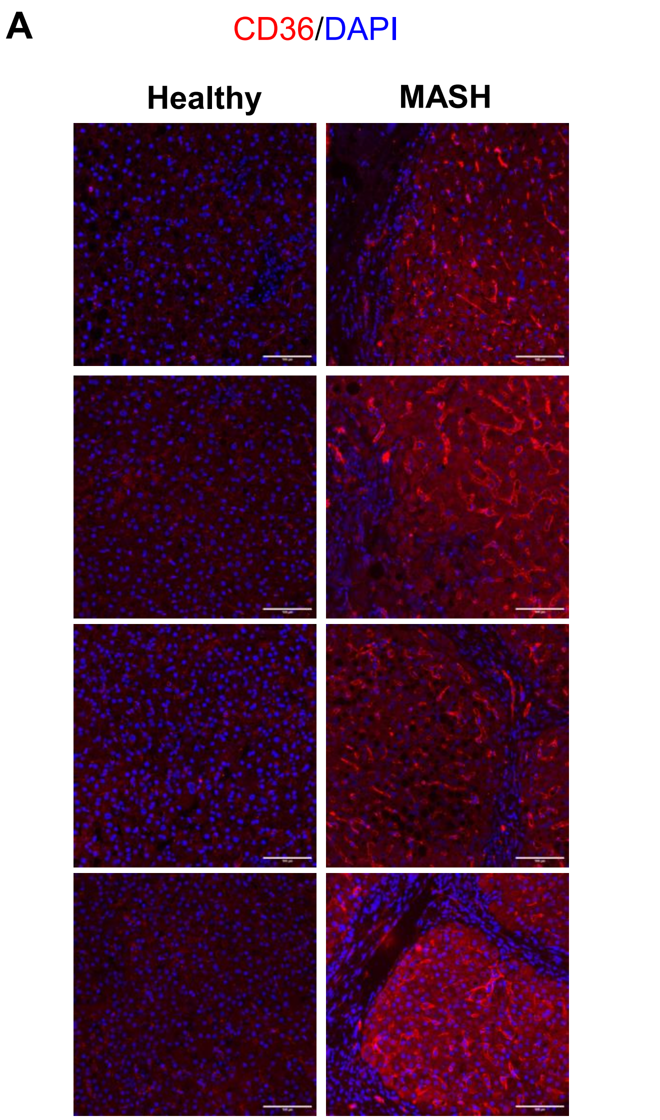

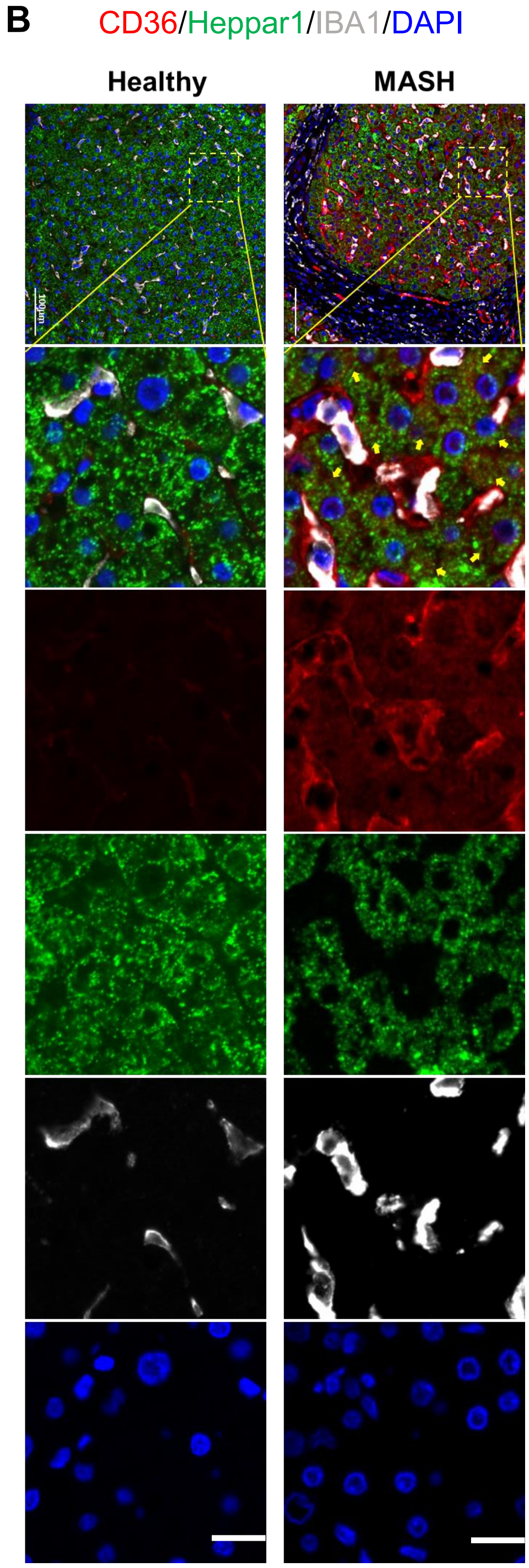

Supplementary Figure S18. The increase of hepatocyte CD36 expression in MASH. Liver tissues from patients with MASH and healthy controls were subjected to immunofluorescence staining for CD36. (A) Representative images from 4 healthy human livers and 4 patient MASH livers show that patients with MASH have higher expression of CD36. (B) The type of cells expressing CD36 was assessed by co-staining of CD36, Heppar1, and IBA1. Representative images are shown. Scale bars: 100 μm. Yellow arrows indicate $CD36^{+}Heppar1^{+}$ hepatocytes.

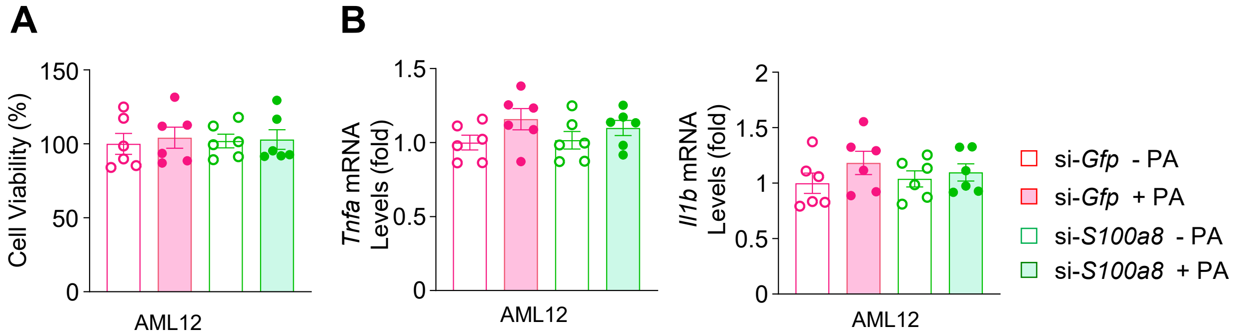

Supplementary Figure S19. Effects of the conditioned media derived from S100A8^{+} macrophages on cell viability and inflammatory gene expression in AML12 cells. (A) Bone marrow-derived macrophages (BMDMs) were transfected with siRNA targeting *Gfp* or *S100a8,* followed by treatment with PA (0.2 mM) or vehicle for 24 h. Conditioned media were then collected and used to culture AML12 cells for 24 h. Cell viability was assessed using CCK-8 assays (n=6; data combined from two independent experiments). (B) AML12 cells were cultured for 24 h in the conditioned media described in panel A. Transcript levels of *Tnfa* and *Il1b* were measured by RT-qPCR (n=6; data combined from two independent experiments).

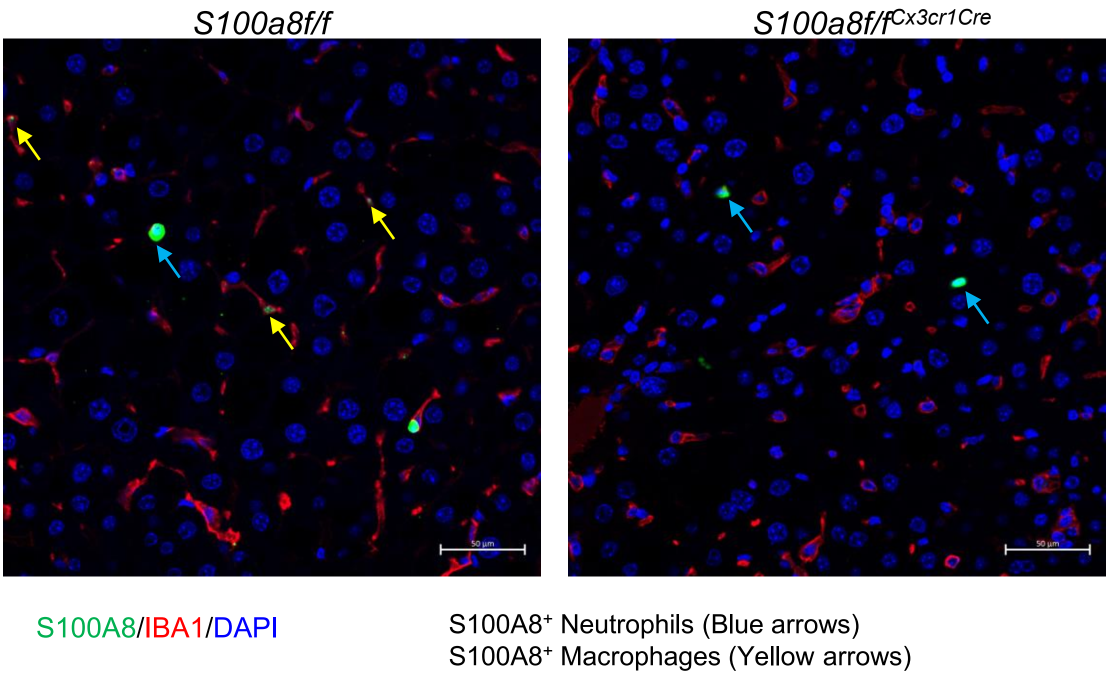

Supplementary Figure S20. Immunohistochemical staining of S100A8 confirms deletion of S100A8 in macrophages but not in neutrophils from *S100a8f/f^{Cx3cr1Cre}* mice. *S100a8f/f^{Cx3cr1Cre}* and *S100a8f/f* littermates were fed an HFD for 3 months (n=5/group). Paraffin-embedded liver sections were analyzed by immunohistochemistry for IBA1 and S100A8. Yellow arrows indicate S100A8^{+} macrophages, and blue arrows indicate S100A8^{+} neutrophils. Scale bars indicate 50 μm.

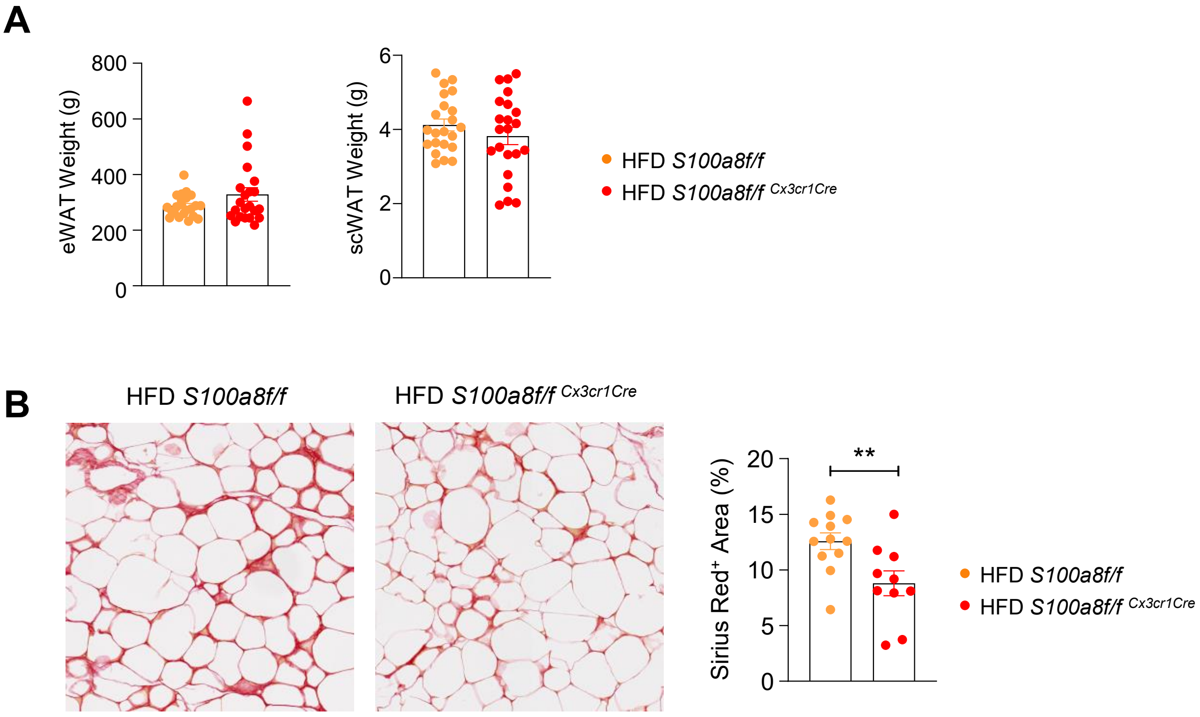

Supplementary Figure S21. Decrease in the adipose tissue fibrosis by macrophage-specific deletion of *S100a8*. *S100a8f/f*^{Cx3cr1Cre} mice and *S100a8f/f* littermate controls were fed an HFD for 4 months. (A) Epididymal fat weight and subcutaneous fat weight. Values represent mean ± SEM. (B) Paraffin-embedded epididymal adipose tissues were subjected to Sirius Red staining. Areas positive for Sirius Red were quantified. **p<0.01. Statistical evaluation was performed by Student's t-test.

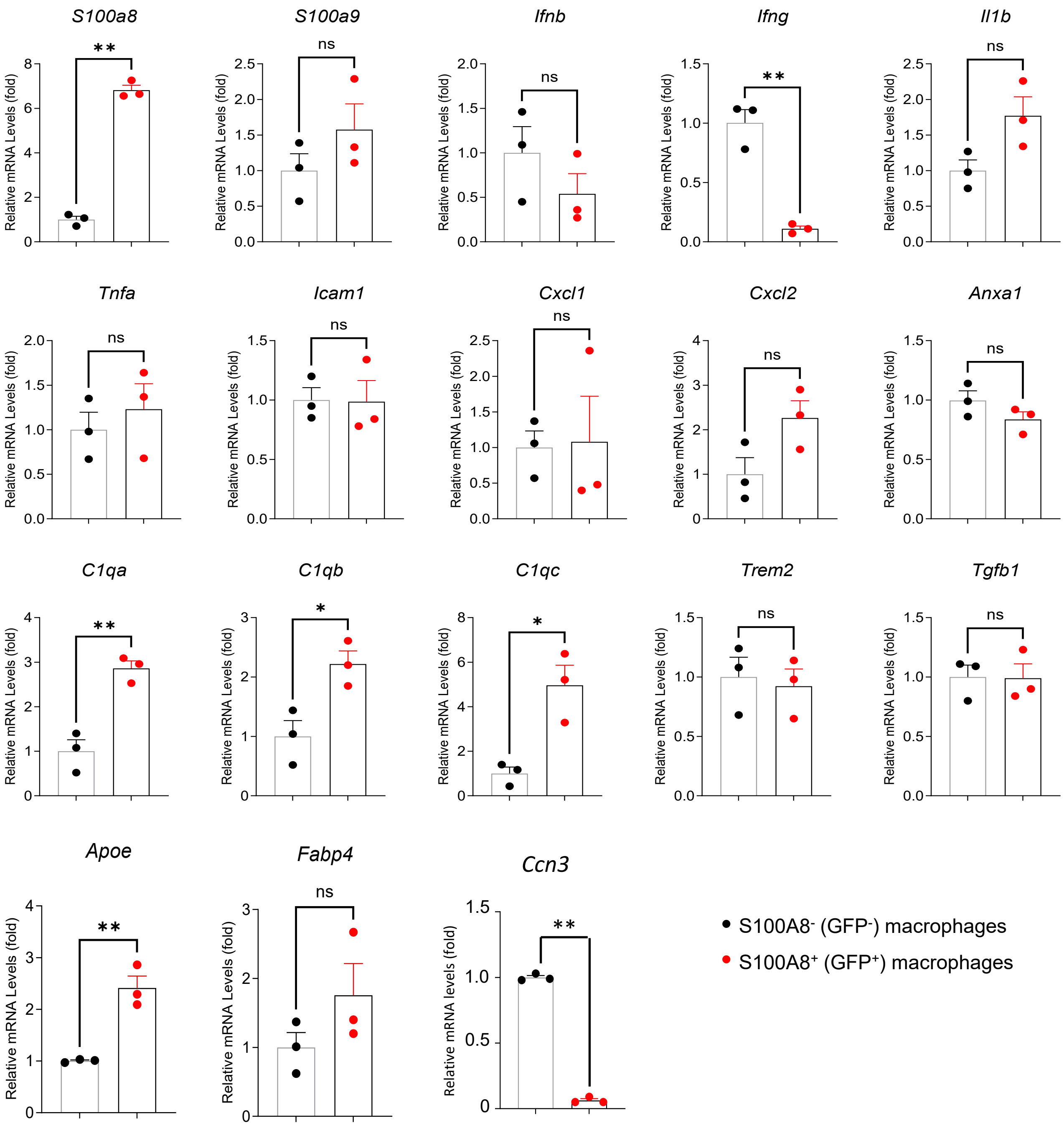

Supplementary Figure S22. Characterization of S100A8⁺ hepatic macrophages isolated from HFD-fed *S100a8*-Cre-ires/GFP mice. *S100a8*-Cre-ires/GFP mice were fed an HFD for 3 months to induce metabolic stress and hepatic macrophage activation. GFP^{+} macrophages expressing S100A8 and GFP^{-} macrophages were sorted from liver tissues by flow cytometry. RT-qPCR analyses were performed on sorted GFP^{+} and GFP^{-} macrophages to assess the expression of inflammatory, lipid-handling genes and *Ccn3* gene. Values represent mean ± SEM. Statistical evaluation was performed by Student's t-test (*p<0.05, **p<0.01).

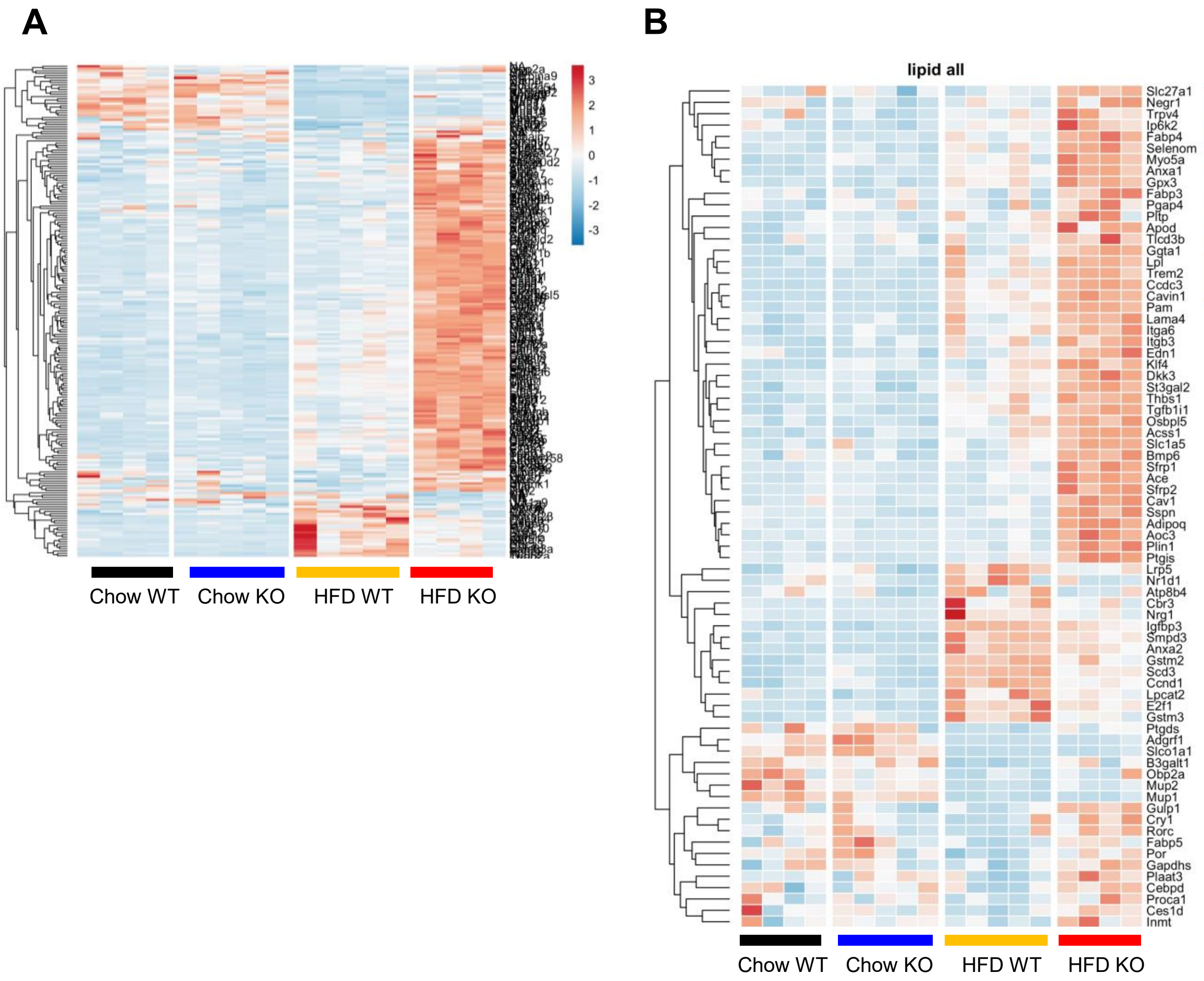

Supplementary Figure S23. RNA-seq analysis of macrophage-specific *S100a8*-deficient mice. *S100a8f/f*^{Cx3cr1Cre} and *S100a8f/f* littermates were fed a chow diet or HFD for 3 months. Liver tissue was analyzed by RNA sequencing. (A) Heatmap of differentially expressed genes between the livers of HFD-fed *S100a8f/f*^{Cx3cr1Cre} and *S100a8f/f* littermates. ($|\log2[\text{fold change}]| > 1$, $p < 0.05$). (B) Heatmap of lipid related genes between the livers of HFD-fed *S100a8f/f*^{Cx3cr1Cre} and *S100a8f/f* littermates. ($|\log2[\text{fold change}]| > 1$, $p < 0.05$).

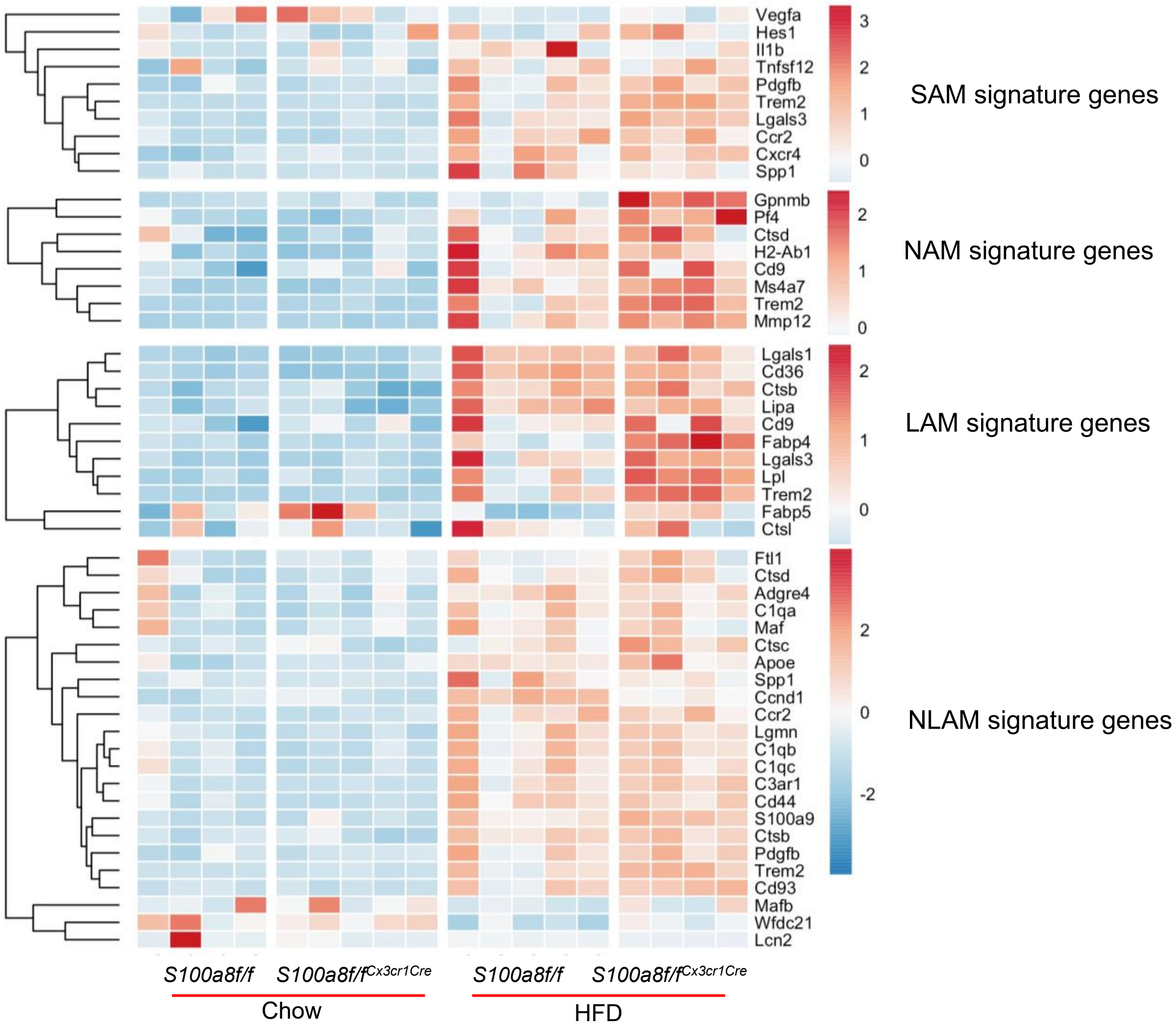

Supplementary Figure S24. RNA-seq analysis reveals compensatory macrophage subpopulation changes following *S100a8* deletion. Heatmaps of signature genes associated with monocyte-derived macrophage populations, including scar-associated macrophages (SAMs), nonalcoholic steatohepatitis–associated macrophages (NAMs), lipid-associated macrophages (LAMs), and necrotic lesion-associated macrophages (NLAMs) in the liver of chow diet- and HFD-fed *Bcl2*^{AdTG} and WT mice.

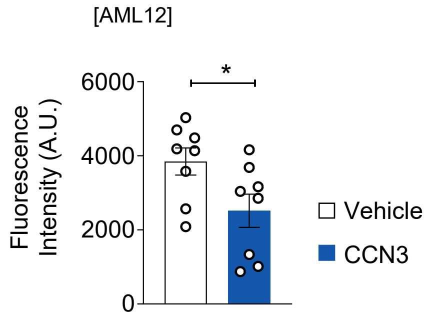

Supplementary Figure S25. Reduced free fatty acid uptake by a CCN3 treatment in AML12 cells. AML12 cells were treated with recombinant mouse CCN3 protein (100 ng/mL) for 24 h. Free fatty acid uptake was analyzed by measuring the fluorescent intensity of a fatty acid probe (n=8; data represent the combined results of two independent experiments). Statistical evaluation was performed by Student's t-test (**p*<0.05).

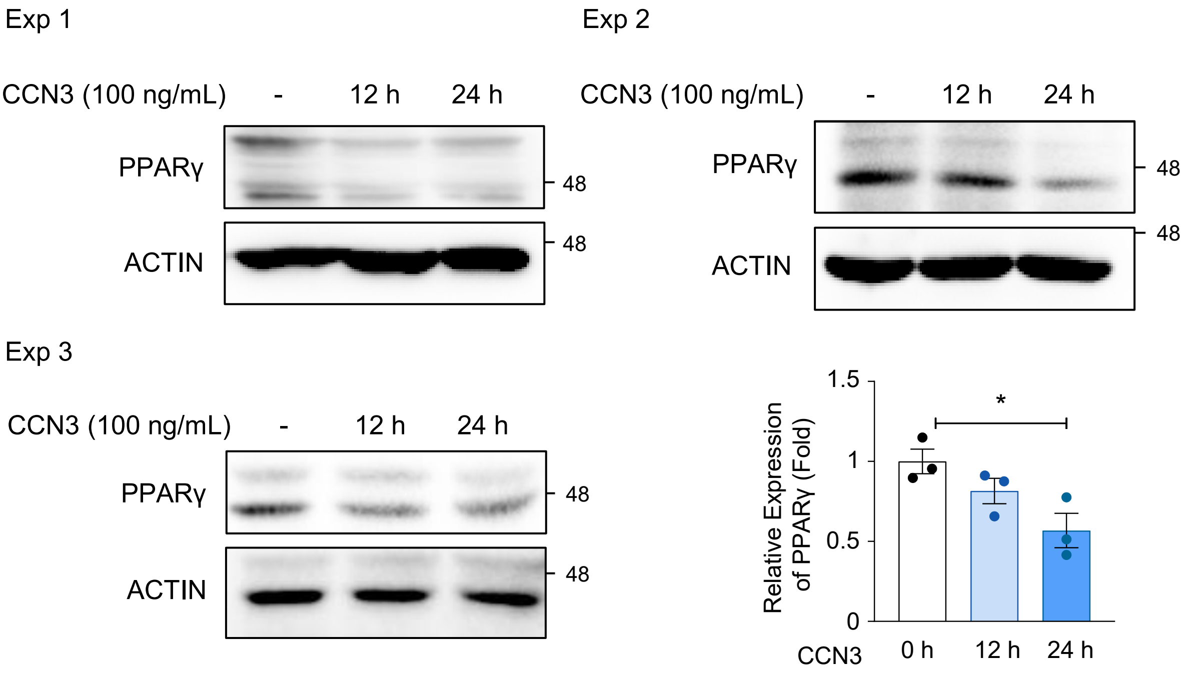

Supplementary Figure S26. Decreased expression of PPARγ by CCN3 in mouse hepatocytes. Primary mouse hepatocytes were treated with recombinant mouse CCN3 protein (100 ng/mL) for 12 h or 24 h. Total cell lysates were subjected to western blot analysis for PPARγ (n=3). The relative expression of PPARγ normalized to β-Actin was quantified. Values represent mean ± SEM from three independent experiments. Statistical evaluation was performed using one-way ANOVA with Tukey's post hoc test for multiple comparisons (**p* < 0.05).

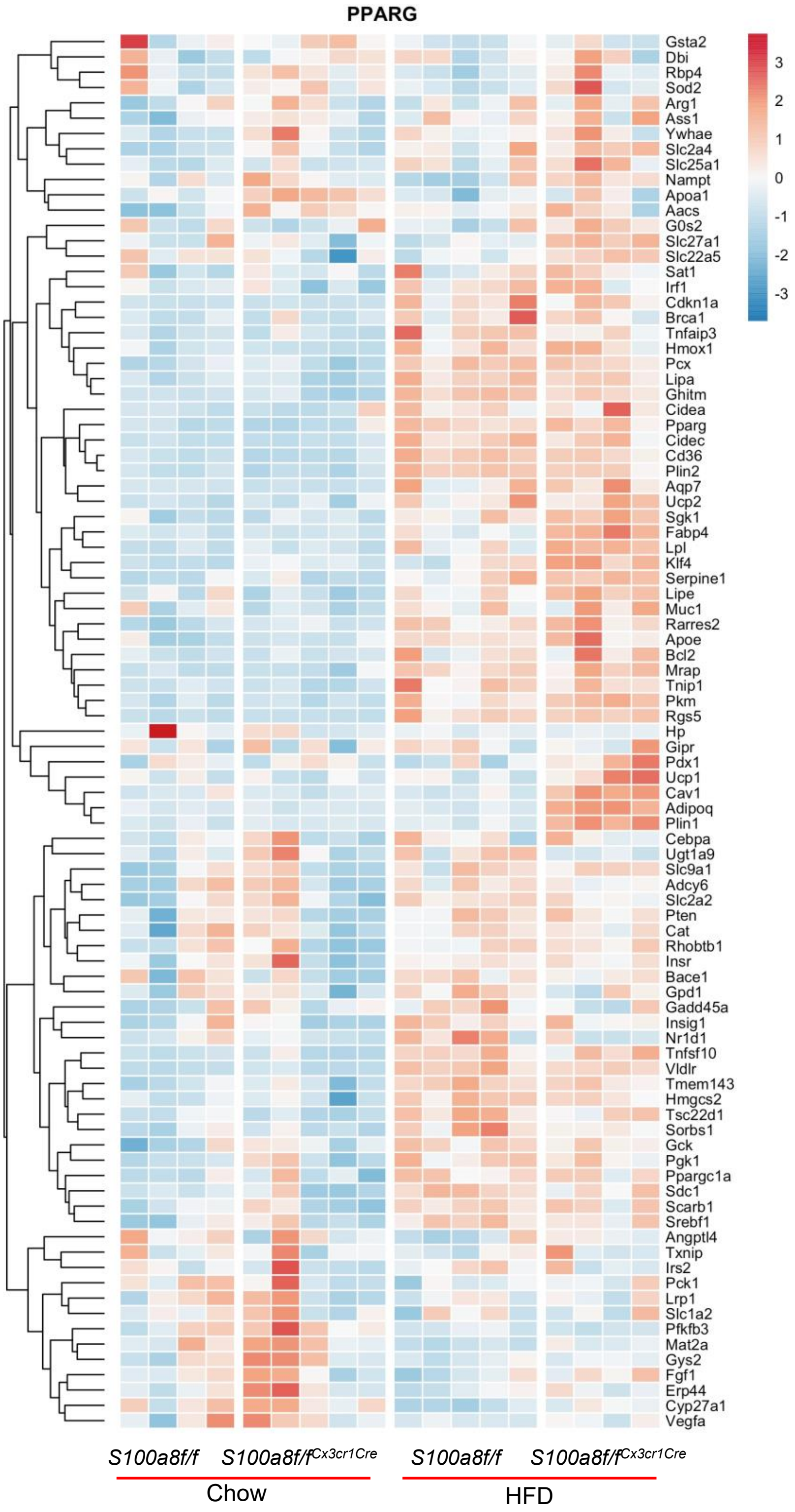

Supplementary Figure S27. Expression of PPARγ target genes in HFD-fed wild-type and *S100a8*-deficient mice. Heatmaps of hepatic PPARγ target genes in the liver of chow diet- and HFD-fed *S100a8f/f*^{Cx3cr1Cre} and *S100a8f/f* littermates.
