# Supplementary figures and images for: Adipocyte death promotes hepatic infiltration of S100A8+ macrophages and steatotic liver disease progression in mice
Source: J Clin Invest. 2025 Nov 3;135(21):e190635. doi: 10.1172/JCI190635 (PMC12578405; doi:10.1172/JCI190635)

## Full unedited blots for Figure 2A

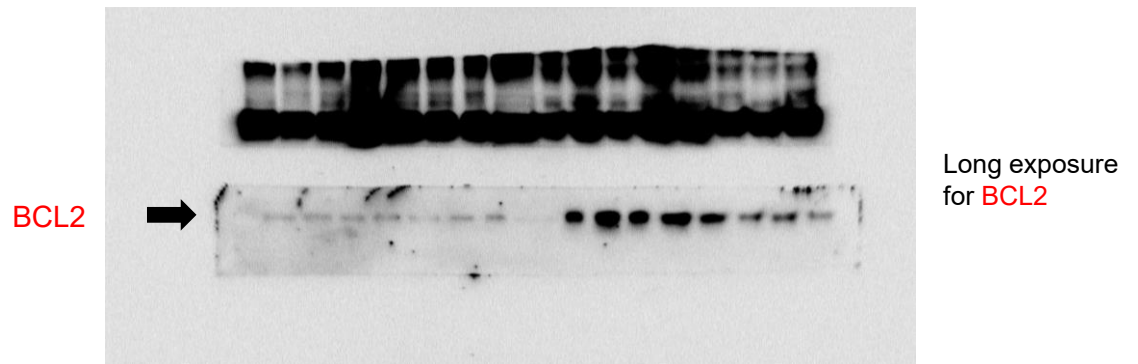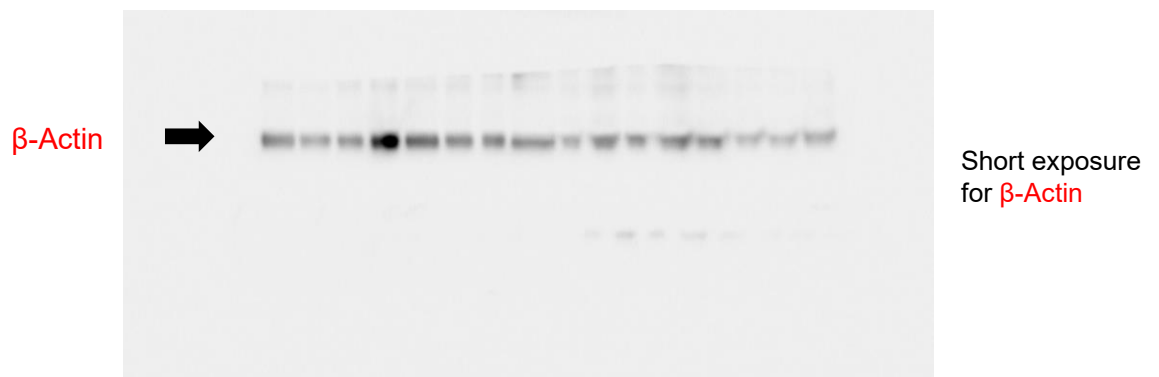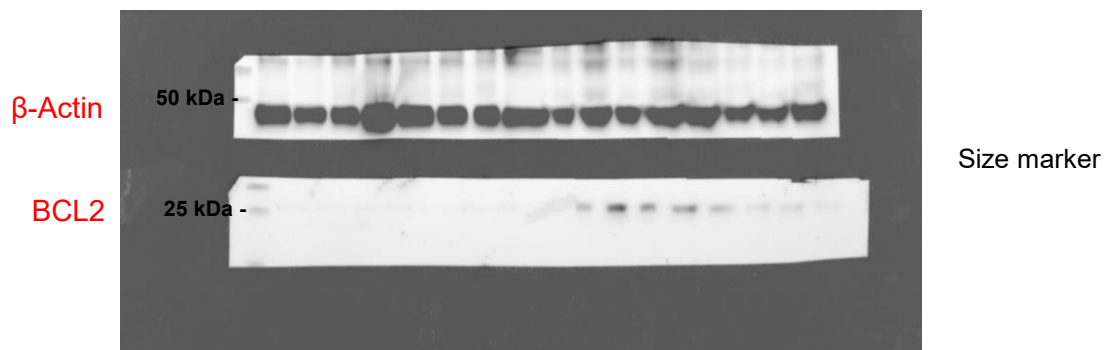

Full unedited blots for Supplementary Figure S27

Blot #1

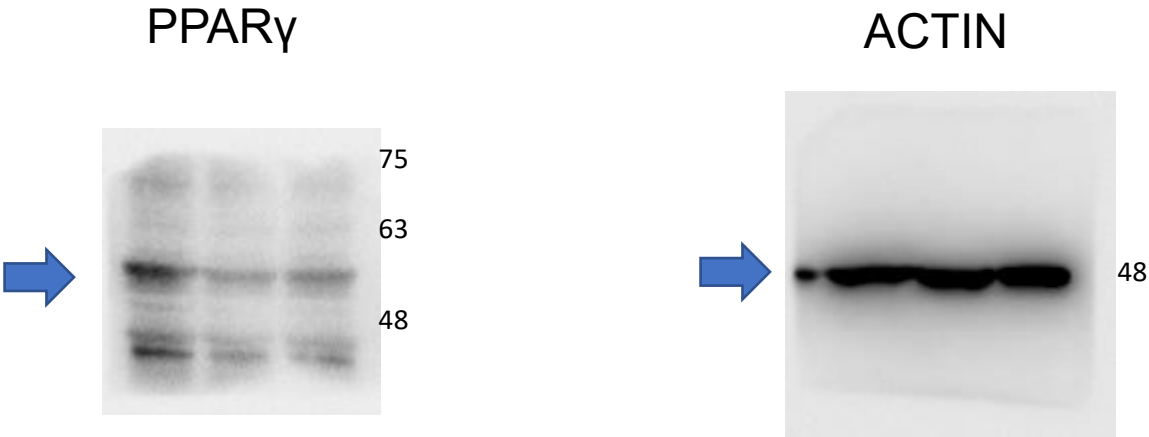

Blot #2

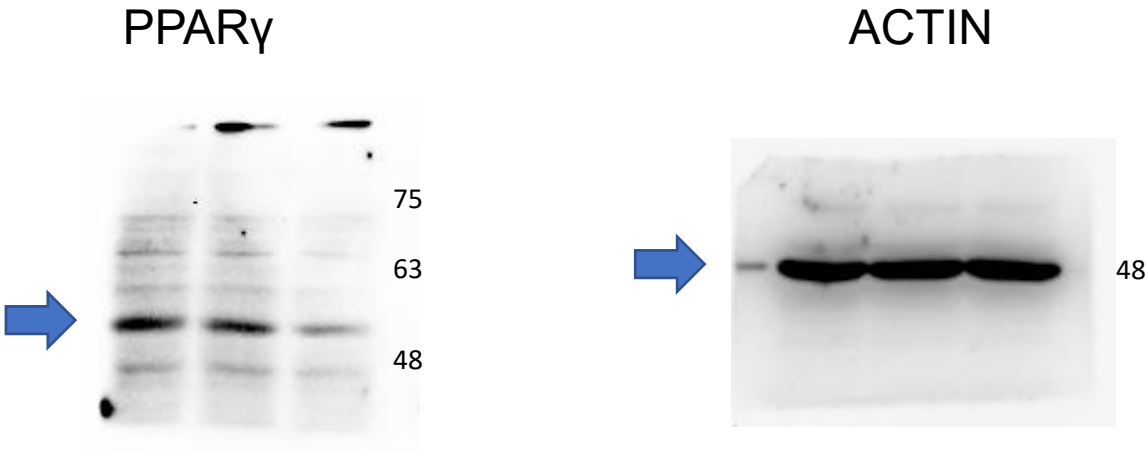

Blot #3

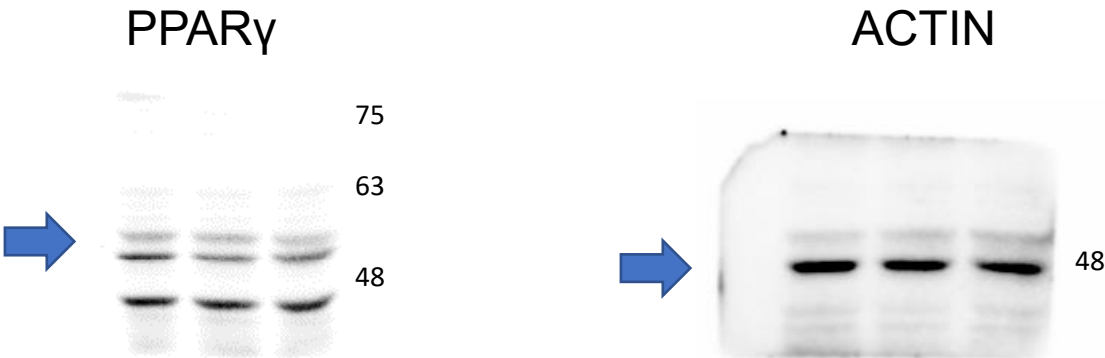

Supplement: Unedited blot and gel images [file jci-135-190635-s213.pdf]
